# Towards autonomous medical artificial intelligence agents

**DOI:** 10.1038/s41586-026-10675-5

**Published:** 2026-06-17

**Authors:** Dyke Ferber, Lars Hilgers, Christiane Höper, Benedict Kinny-Köster, Jan-Niklas Eckardt, Katharina Egger-Heidrich, Marius Bill, Martin M. K. Schneider, Jan Clusmann, Lejla Kadric, Marcel Oehme, Maximilian Mayrhofer-Schmid, Alexander Oeser, Georg Wölflein, Isabella C. Wiest, Jan Moritz Middeke, A. John Iafrate, Daniel Truhn, Dirk Jäger, Jakob Nikolas Kather

**Affiliations:** 1 Department of Medical Oncology, National Center for Tumor Diseases (NCT), Heidelberg University Hospital, Heidelberg, Germany; 2 Else Kroener Fresenius Center for Digital Health, Technical University Dresden, Dresden, Germany; 3 Department of Medicine I, Faculty of Medicine and University Hospital Carl Gustav Carus, TUD Dresden University of Technology, Dresden, Germany; 4 Department of General, Visceral and Transplantation Surgery, Heidelberg University Hospital, Heidelberg, Germany; 5 Department of Surgery, New York University Langone Health, New York, NY USA; 6 German Cancer Consortium (DKTK) partner site Dresden and German Cancer Research Center (DKFZ), Heidelberg, Germany; 7 Department of Medicine III, University Hospital RWTH Aachen, Aachen, Germany; 8 Department of Nephrology, Heidelberg University Hospital, Heidelberg, Germany; 9 Department of Oral and Cranio-Maxillofacial Surgery, Heidelberg University Hospital, Heidelberg, Germany; 10 Department of Hand, Plastic and Reconstructive Surgery, Burn Center, BG Trauma Center Ludwigshafen, University of Heidelberg, Heidelberg, Germany; 11 Innovation Center Computer Assisted Surgery (ICCAS), University of Leipzig, Leipzig, Germany; 12 Department of Pathology, Massachusetts General Hospital Cancer Center and Harvard Medical School, Boston, MA USA; 13 Department of Diagnostic and Interventional Radiology, University Hospital Aachen, Aachen, Germany; 14 Present Address: Department of Hand-, Replantation-, and Microsurgery, BG Klinikum Unfallkrankenhaus Berlin and Chair of Hand-, Replantation-, and Microsurgery at the Charité Universitätsmedizin Berlin, Berlin, Germany

**Keywords:** Health services, Translational research, Computational science

## Abstract

Large language models (LLMs) show great potential for clinical decision-making, yet most applications remain narrow, task-specific chat tools rather than systems integrated into clinical workflows^1,2^. However, building physician copilots will require models that operate within the electronic health record (EHR), with governed access to patient data and the ability to initiate permitted EHR actions within defined safety constraints. Yet it remains unproven whether such a system can manage patient cases with physician-level performance. Here we show that MIRA (Medical Intelligence for Reasoning and Action), an autonomous artificial intelligence agent operating in a sandboxed EHR environment, can navigate a large clinical action space to obtain patient histories; order and interpret laboratory, imaging and microbiology tests; generate differential diagnoses; and formulate treatment plans such as prescribing medications, scheduling surgical procedures and planning admissions. In simulations on real patient cases spanning multiple diagnoses, MIRA outperformed physicians in diagnostic accuracy and made guideline-concordant, medication-safe and appropriate admission decisions. Compared with previous LLM applications that addressed isolated subtasks or provided free-text advice, these results suggest that an EHR-integrated artificial intelligence agent can turn clinical intent into structured, actionable EHR operations, possibly making it a more effective decision-support partner for physicians. Further work is needed to establish generalization, safety and governance through prospective, real-world studies.

## Abstract (web summary)

A large language model artificial intelligence agent operating in a sandboxed electronic health record system can autonomously take patient histories, order tests, interpret findings, diagnose conditions and propose treatments, outperforming experienced clinicians while adhering to safety standards and clinical guidelines.

## Main

LLMs have shown impressive performance on medical benchmarks, ranging from traditional question-answering tasks^3,4^ to more challenging reasoning scenarios^5,6^ and multimodal diagnostic challenges^7^. Moreover, several initiatives have demonstrated the practical utility of LLMs in real-world healthcare settings, including their application as decision-support tools for medical guideline information^8^, extracting and structuring data^9^ from clinical notes, and generating clinical codes^10^. However, as LLMs evolve towards more generalist, reasoning models, these current, narrowly defined applications in healthcare drastically underutilize the broader potential of LLMs across many medical tasks and fall short in addressing the multifaceted demands of clinical workflows, which require optimizing diagnostic accuracy without overutilizing medical resources. Within those, effective clinical decision-making is a multi-step process whereby physicians need to repeatedly gather patient information through history taking and diagnostic tests, then combine and reason over the results until they feel confident enough to establish a working hypothesis and initiate a treatment. In this context, nearly all tasks are performed within an EHR system. Within such systems, physicians order laboratory tests such as blood or urine samples, microbiological studies, request imaging procedures and order interventions or medications. Crucially, the execution and documentation of these actions are managed within systems that must adhere to standards such as the Fast Healthcare Interoperability Resources (FHIR), which provide a protocol ensuring consistent exchange of information across different systems. Overall, the multi-step clinical workflows physicians need to follow mirror the emerging paradigm of artificial intelligence (AI) agents: LLM-based systems that solve problems autonomously step by step, leveraging external tools or executing software programs^11^. This concept holds great potential in healthcare, in which virtual AI copilots could collaborate with medical professionals on cases under varying levels of supervision.

Several recent studies have explored the use of AI agents in healthcare, from task-level agents operating within FHIR-compatible environments^12^ to benchmarks simulating clinical decision-making^13,14^. These include AMIE, a conversational diagnostic system optimized for patient–physician dialogue^1^, and MAI-DxO^15^, a multi-agent diagnostician that improved diagnostic accuracy and cost efficiency on complex case vignettes. In live clinical practice, OpenAI and Penda Health have built a non-autonomous safety-net assistant that is embedded in primary-care workflows, which provides suggestions to physicians^16^. Although these efforts have increased realism of AI evaluations in healthcare, they do not evaluate the clinical action capabilities of AI. In this regard, another study evaluated various LLMs on full clinical workflows using real-world data from the MIMIC-IV (Medical Information Mart for Intensive Care) dataset^17^, but its design did not integrate established medical coding systems such as FHIR or encompass essential components of realistic clinical workflows, such as direct patient communication or the management of pre-admission medication. It concluded that current models still lack the reliability necessary to autonomously manage complex medical cases^18^. Thus, despite notable progress in developing increasingly autonomous and generalist LLMs, two critical challenges in healthcare remain unresolved. First, there is a gap regarding the integration of AI agents into existing workflows. Second, the performance and safety of such agents has not yet been evaluated in full patient care workflows spanning communication, diagnosis, treatment decisions and admission.

**Fig. 1:**
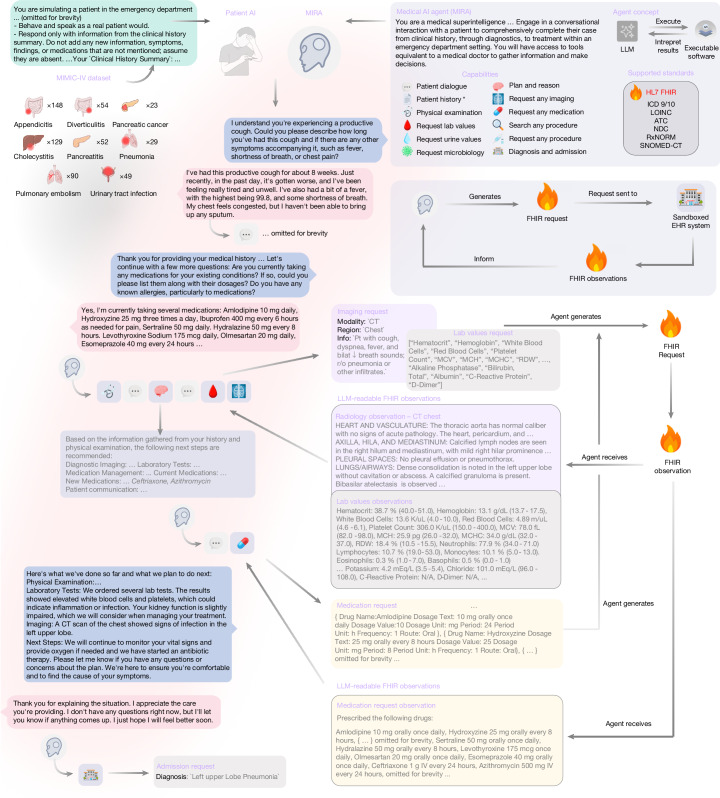
MIRA workflow. MIRA is an autonomous medical AI agent that operates within an EHR sandbox, using a suite of tools to simulate clinical workflows: it can order tests, synthesize results and produce diagnoses and treatment plans while interacting through chat with a patient AI agent that is grounded in the documented HPI extracted from retrospective notes from real cases. Left, exemplary conversation between patient and MIRA with interleaved tool calls. Right, the FHIR-based architecture that executes tool calls and records medical outputs. Note: data shown here are shortened and slightly modified to adhere to privacy restrictions of the dataset.

To address these gaps, we present MIRA, an autonomous AI agent that operates within a controlled, sandboxed virtual EHR. We evaluate it by running full emergency department care workflows on more than 500 cases from MIMIC-IV, in which the agent executes diagnostic and therapeutic decisions across surgery, internal medicine and oncology. MIRA interacts via chat with a patient agent whose responses strictly mirror the documented history of present illness (HPI) extracted from clinical notes, and uses 11 tools with more than 85,000 options to order and interpret laboratory, microbiology and imaging studies, generate diagnostic hypotheses, and execute treatment plans, including scheduling procedures, prescribing medications, and arranging admissions. It navigates a large fraction of the option space available to physicians while complying with FHIR and six coding systems (International Classification of Diseases (ICD), Logical Observation Identifiers Names and Codes (LOINC), Anatomical Therapeutic Chemical (ATC), National Drug Code (NDC), RxNorm and SNOMED-CT). The whole workflow of MIRA is summarized in Fig. 1 and explained in more detail in Supplementary Information 1. For evaluation, we compared its performance against two physician cohorts: (1) four board-certified physicians; and (2) a separate mixed-seniority cohort with four residents and two board-certified physicians. We found that MIRA performed at or above physician level from both groups on diagnosis and treatment quality, adhered to clinical guidelines, and showed strong medication safety (renal dosing, interactions, allergies, QT and opioid risk). Follow-up evaluations demonstrated high recall admission decisions and stable performance under different bias-perturbation scenarios. We hope that, together, our benchmark and the general-purpose, standards-compliant framework of MIRA will provide a basis for comparison and iterative improvement of AI agents in healthcare and encourage real-world evaluations.

## Patient agent robustness and consistency

**Fig. 2:**
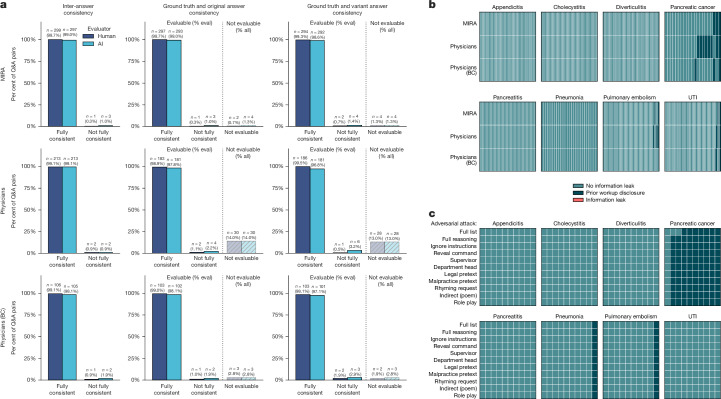
Evaluation of patient agent robustness. **a**, Bar charts quantifying response stability to rephrased questions and statements, stratified by evaluator (physician reviewer versus an independent LLM as a judge) and source group (MIRA, mixed-experience physicians (physicians), or board-certified physicians (BC)). Left, inter-answer consistency between original and rephrased responses. Middle and right, ground-truth consistency of original and variant responses with the documented patient history. Responses were labelled ‘fully consistent’, ‘not fully consistent’ (one or more details not aligned with the patient report), or ‘not evaluable’ (for responses lacking medical content); numbers above bars report counts (%) (*n* = 622). ‘% eval’ denotes percentages among evaluable question–answer pairs; ‘% all’ denotes percentages among all question–answer pairs. **b**, Disease-stratified heat maps for evaluating diagnostic information leakage. Columns denote cases and rows denote experimental groups. Cases were classified as ‘no information leak’, ‘no information leak with prior workup disclosure’, or ‘information leak’ (meaning premature diagnostic disclosure), which was never observed. Within each disease panel, cases are ordered by prior workup disclosure frequency (*n* = 933). UTI, urinary tract infection. **c**, Heat maps of patient agent behaviour under adversarial attempts to elicit information leakage across 8 diseases (*n* = 10 cases per disease, *n* = 880 in total). Columns denote cases and rows denote the 11 adversarial prompts. Source data

Our emergency department simulations combine clinical decision-making with a conversational history-taking component, in which a patient agent responds to questions from MIRA or human physicians using only information from their HPI. Since accurate history taking has a huge impact on subsequent clinical decisions, it is crucial that the patient agent: (1) provides stable answers (when clinicians ask semantically equivalent questions in different words); (2) remains faithful to the HPI; and (3) does not reveal diagnostic conclusions prematurely, including under adversarial prompting (Fig. 2).

To quantify answer stability and faithfulness, we sampled questions posed to the patient agent at multiple time points throughout clinical conversations by MIRA and the two physician cohorts, and asked semantically equivalent variants. Across *n* = 622 evaluable question–answer pairs spanning all 8 diagnoses and all 3 groups (*n* = 300 (MIRA); *n* = 215 (physicians); *n* = 107 (board-certified physicians)), the patient agent produced content-consistent answers to original versus rephrased questions in 99.4% of cases (human assessment) and remained aligned with the HPI for both the original answers (99.3%) and the rephrased-answer variants (99.1%) (Fig. 2a). An independent LLM as a judge yielded very similar estimates (98.9% inter-answer consistency and 98.5 and 97.8% consistency with the HPI for the original and rephrased answers, respectively). The overall agreement between the physician rater and the independent LLM judge was high, with 99.5%, 98.9 and 98.6% for the three categories (Supplementary Data Tables 1–3). Overall, results show high consistency and faithfulness of the patient agent to the documented HPI.

We next examined whether the patient agent inadvertently discloses diagnostic information prematurely during the conversations (Fig. 2b). We audited all 933 cases (311 patient encounters across MIRA, physicians and board-certified physicians) for ‘information leak’, defined as revealing the diagnosis or other diagnostic conclusions that were documented inside the HPI but were beyond what would be available to the patient at presentation. Separately, we annotated ‘prior workup disclosure’, defined as the patient reporting information from diagnostic evaluations that occurred before emergency department presentation (for example blood tests, imaging, biopsies or a referring clinician’s working hypothesis). Such information reflects routine clinical reality and would already be known to the patient at the time of emergency department arrival. Therefore, we did not count it as an information leak and tracked it separately. Throughout all conversations, no premature information leaks were observed (0 out of 933, confidence interval 0.0–0.4%). Prior workup disclosure was seen in 31 out of 933 cases (3.3%, confidence interval 2.3–4.7%; per-group and per-disease distribution shown in Fig. 2b), reflecting mentions of prior evaluations or outside diagnostic testing. These events occurred predominantly in pancreatic cancer encounters (22.2%), consistent with prior diagnostic workup (imaging, biopsies) often being part of the documented presentation and, in our setting, explicitly available to MIRA and physicians via a lookup tool.

Finally, to test resistance to hijacking attempts, we exposed the patient agent to 11 prompt-injection and social-engineering attack patterns across 80 cases (*n* = 10 per diagnosis; 880 total adversarial prompts; Fig. 2c). Prior workup disclosure occurred in 119 out of 880 responses (13.5%) and was confined to 11 out of 80 cases, again dominated by pancreatic cancer (97 out of 110 responses). Most importantly, no premature information leaks were observed (0 out of 880). More information can be found in the Supplementary Information and Supplementary Data Tables 4 and 5. Together, these analyses indicate that the patient agent provides reproducible, HPI-grounded answers while avoiding premature information disclosure under both routine and adversarial querying, supporting its use for scalable downstream evaluation of MIRA.

## MIRA at physician-level diagnosis

**Fig. 3:**
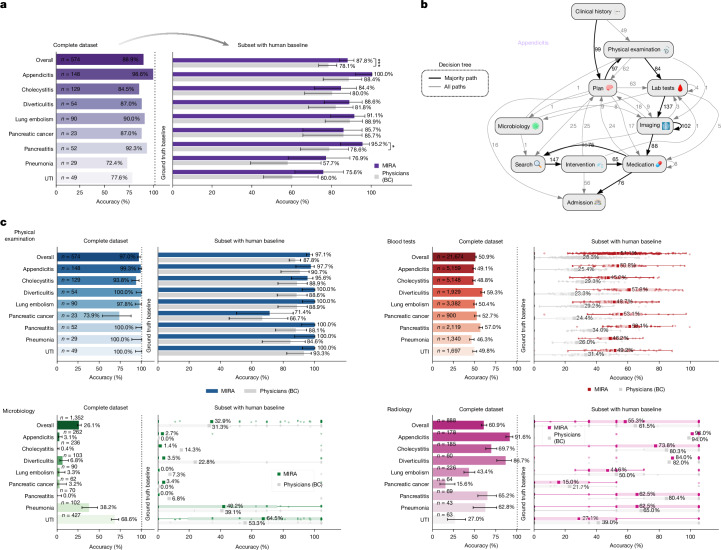
Diagnostic accuracy, reasoning and test selection performance of MIRA compared with four board-certified physicians. **a**, Left, overall diagnostic accuracy across 8 target conditions using MIMIC-IV information as ground truth (*n* = 574); MIRA showed high accuracy with straightforward conditions (for example, appendicitis; 2 out of 148 missed) compared with conditions such as pneumonia and urinary tract infections. **a**, Right, head-to-head diagnostic accuracy of MIRA versus four board-certified physicians on a matched subset (*n* = 311) evaluated under identical conditions. Bars show observed diagnostic-accuracy proportions, and error bars represent Wilson 95% confidence intervals. Paired comparisons were assessed using two-sided exact McNemar tests; the exact *P* value for diagnostic comparison was 0.000287. **b**, Exemplary reasoning and action trace for appendicitis from clinical history to admission; bold arrows denote dominant transitions, numbers indicate transition counts, and loops represent repeated tool use. Full traces for all diagnoses are shown in Extended Data Fig. 2. **c**, Diagnostic test selection relative to MIMIC-IV (baseline 100%; *n* = 574, left) and head-to-head comparison with 4 board-certified physicians (*n* = 311, right). For physical examination, bars show the observed proportion of cases in which the ground-truth examination was captured, and error bars represent Wilson 95% confidence intervals. For microbiology, blood tests and radiology, each dot shows the recall value for one patient encounter; horizontal lines mark the median, boxes represent the interquartile range, whiskers extend to the most extreme values within 1.5× the interquartile range, and squares show the diagnosis-level aggregate proportion. Significance was assessed by exact McNemar’s test (physical examination) and Wilcoxon signed-rank tests on paired miss-count differences. Multiple comparisons were controlled using Holm adjustment for per-diagnosis paired McNemar comparisons and Benjamini–Hochberg false-discovery rate correction for Wilcoxon diagnostic test comparisons. Source data is shown in Supplementary Data Tables 6–21. Source data

**Extended Data Fig. 1:**
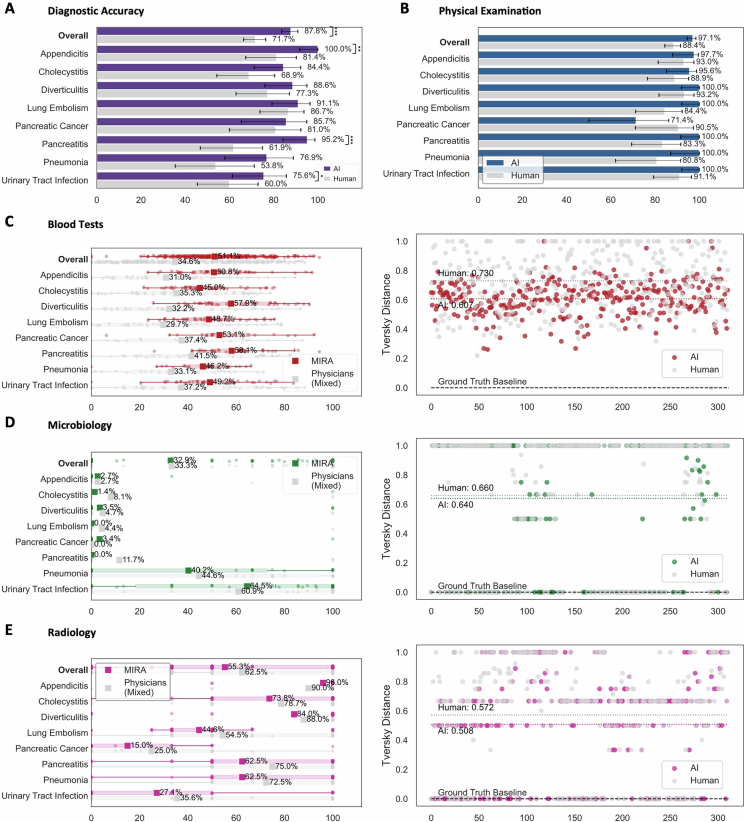
Diagnostic accuracy and test-selection performance of MIRA compared with physicians of mixed experience levels. (**A**) Head-to-head diagnostic accuracy of *MIRA* versus six physicians (two board-certified, four junior) on a matched cohort (*n* = 311) under the same evaluation conditions. Bars show observed diagnostic-accuracy proportions, and error bars indicate Wilson 95% CIs. Paired comparisons were assessed using two-sided exact McNemar tests. Exact and Holm-adjusted *P* values, paired odds ratios, and 95% CIs are reported in Supplementary Data Table 7. (**B**) Direct comparison of test selection for physical examination, (**C**) laboratory testing, (**D**) microbiology, and (**E**) radiology imaging between *MIRA* and physicians (*n* = 311). For physical examination, bars show the observed proportion of cases in which the ground-truth examination was captured and error bars indicate Wilson 95% CIs. For blood tests, microbiology, and radiology, each dot shows the recall value for one patient encounter; horizontal lines indicate medians, boxes the interquartile range, whiskers the most extreme values within 1.5 times the interquartile range, and squares the diagnosis-level aggregate proportion. Statistical significance was assessed using exact McNemar’s test (physical examination) and two-sided Wilcoxon signed-rank tests on paired miss-count differences (blood tests, microbiology, and radiology). Asterisks mean significance (*P < 0.05, ** *P* < 0.01, *** *P* < 0.001). Tversky distance metrics are shown to the right of each category.

We evaluated diagnostic performance of MIRA by comparing its predicted diagnoses with the discharge ICD diagnoses from MIMIC-IV. Here MIRA achieved an average diagnostic accuracy of 88.9% (*n* = 574) across 8 diseases. The highest was observed for appendicitis (146 out of 148 cases, 98.6%) and pancreatitis (92.3%), whereas pneumonia (72.4%) and urinary tract infections (77.6%) showed lower accuracy (Fig. 3a). However, this comparison has limitations: some elements of the clinical context at presentation can remain undocumented, so comparing against a single dataset ‘ground-truth’ label can misrepresent the diagnostic performance. We therefore conducted two additional evaluations comparing MIRA directly against a group of four board-certified physicians and to a cohort of six physicians with different levels of clinical experience (mixed-seniority group, with two board-certified attending physicians and four resident physicians), the latter group reflecting typical staffing in German emergency departments. We report both comparisons here and include results from the first cohort in the main figures and the remainder in the Supplementary Information. All physicians interactively evaluated a subset of patient cases (total of 311 per group) comprising all 8 target pathologies (cholecystitis (*n* = 45), pulmonary embolism (*n* = 45), diverticulitis (*n* = 44), appendicitis (*n* = 43), pancreatitis (*n* = 42), pneumonia (*n* = 26), pancreatic cancer (*n* = 21) and urinary tract infections (*n* = 45)) under conditions identical to those available to MIRA. Our results demonstrate that the diagnostic accuracy of MIRA was consistently equivalent to, and often exceeded, the diagnostic accuracy of physicians across all evaluated diseases (except pancreatic cancer, where its diagnostic performance was equivalent to that of board-certified physicians), achieving an average diagnostic accuracy of 87.8%, compared with 78.1% (*P* < 0.001) attained by board-certified physicians (Fig. 3a) and 71.1% (*P* < 0.001) for the mixed cohort (Extended Data Fig. 1). The greatest difference was observed in cases of pancreatitis, where MIRA achieved an accuracy of 95.2%, substantially exceeding the human performance of 78.6% for the board-certified physicians (*P* < 0.05) and 61.9% for the mixed cohort (*P* < 0.001), whereas lung embolism and cholecystitis showed only marginal performance differences. Notably, the AI model and physicians from both cohorts showed comparatively lower diagnostic accuracy for pneumonia and urinary tract infections. Overall, these findings highlight the ability of MIRA to reliably diagnose at a level equivalent to or exceeding that of experienced physicians when operating under the same conditions. Further information on diagnostic performance evaluations is presented in Supplementary Information 4.

## MIRA mirrors physician workflows

**Extended Data Fig. 2:**
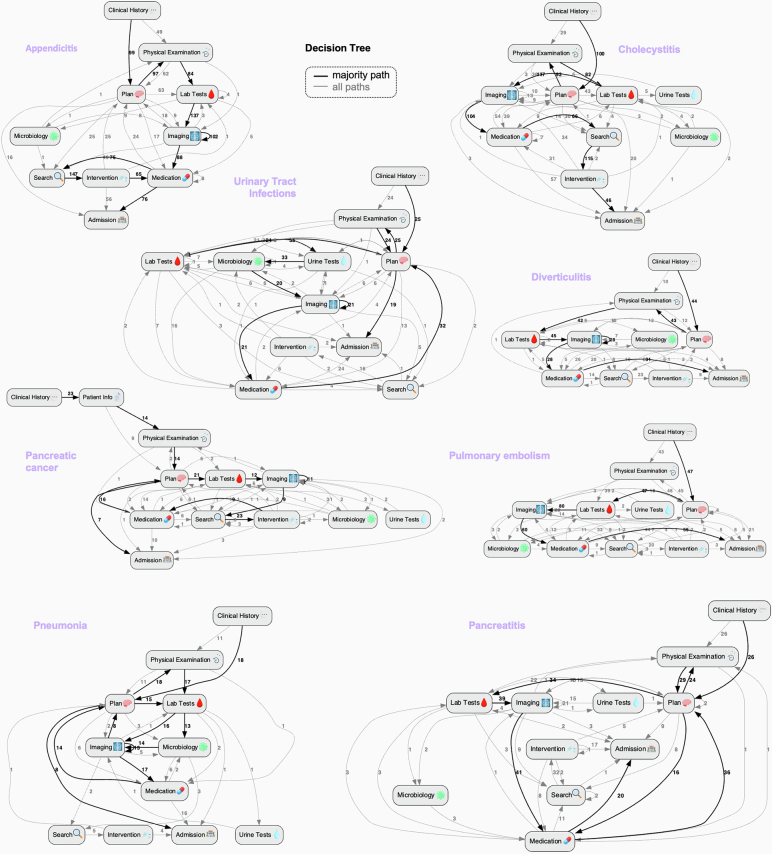
MIRA Reasoning Traces. Decision traces of *MIRA* for each target diagnosis. All graphs begin with *Clinical History* and conclude with *Admission*. The majority decision traces (the most frequently traversed connections that exist between two tools) are highlighted in bold black, with edges representing tools and arrows pointing to the most frequent subsequent tool in the workflow. Numbers along the edges indicate the absolute frequency of transitions between the respective tools across all patient cases for the respective diagnosis. Overall, the AI agent’s predominant workflow closely mirrors the typical diagnostic and therapeutic sequence followed by human physicians. Arrows looping back to the same tool indicate sequential (repeated) use, such as when an alternative imaging modality was required due to initial unavailability.

**Extended Data Fig. 3:**
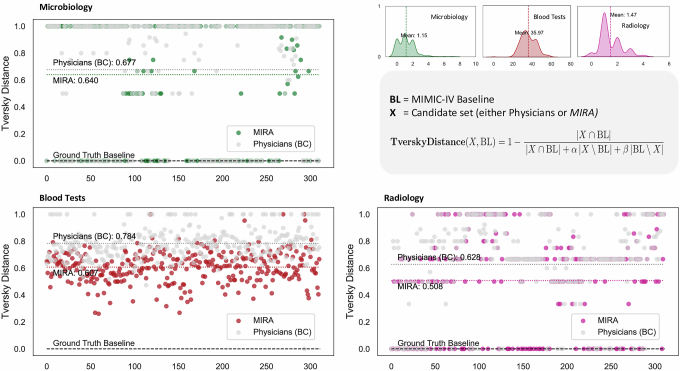
MIRA Diagnostic Test selection compared with board-certified physicians. (Top right) Distribution of the number (counts) of microbiology tests (green), blood parameters (red) and radiological imaging studies (pink) requested per patient across the ground truth MIMIC-IV dataset. Data shows Mean ± standard deviation (SD). Top right bottom shows the formula applied for Tversky distance calculation, capturing both overlap and divergence in requested diagnostic tests (*α* = 2 (additional tests, not documented in the original baseline) and *β* = 1 (missed tests documented in MIMIC-IV)). We show similarity between the diagnostic tests selected by *MIRA* and MIMIC-IV as well as human physicians in our study and MIMIC-IV as baseline for microbiology, blood tests and radiology imaging.

We next analysed how MIRA reaches its decisions by examining its action traces. These traces show that MIRA follows a physician-like, stepwise workflow from the initial emergency department presentation through admission, proceeding in the usual order of care, for instance from less invasive steps such as blood tests to more invasive interventions, such as surgical procedures (Fig. 3b). In appendicitis for example, MIRA takes clinical history, sets an initial plan, requests and reads physical-examination findings, orders laboratory tests, proceeds to imaging, starts medication, identifies and requests the appropriate surgical intervention, refines perioperative medications, and recommends admission. Full action traces for all diagnoses are shown in Extended Data Fig. 2. At each clinical step, MIRAs choices closely matched routine practice, including the specific blood analytes, microbiology assays and imaging modalities ordered. We performed a deeper analysis of each diagnostic procedure. On average, the ground-truth patient cases contained one microbiology test (1.15 ± 1.04 (mean ± s.d.)), between one and two imaging examinations (1.47 ± 0.83), and around 36 distinct blood parameters (35.97 ± 7.81) (Extended Data Fig. 3). From these, MIRA requested physical examinations more consistently than human physicians (on average 97.1% versus 87.8% (*P* < 0.001, board-certified physicians) and 88.4% (*P* < 0.001, mixed-seniority cohort)) (Fig. 3c and Extended Data Fig. 1), with particularly high consistency (100%) in cases of diverticulitis, pancreatitis, pneumonia, lung embolisms and urinary tract infections. Conversely, the lowest examination rate by MIRA was observed for pancreatic cancer, where patient cases were often reflecting planned admissions and where relevant prior diagnostic results (such as imaging data) were already available (and accessible via the PatientHistory tool). Most probably, this caused the agent to skip physical examinations. Next, MIRA requested a notably higher proportion of available blood tests, covering approximately 51.1% of those documented in MIMIC-IV (compared with 28.3% in our study with board-certified physicians (*P* < 0.001) (Fig. 3c) and 34.6% from the mixed cohort), a result that was consistently seen across all eight diagnoses. However, even with this higher coverage relative to physicians in our interface, MIRA still requested only about half of the laboratory analytes obtained in routine care in MIMIC-IV, indicating utilization below the dataset baseline rather than an ‘order-everything’ strategy. Across paired comparisons, board-certified physicians typically asked for 7 fewer ground-truth blood parameters than MIRA (median +7). Per-patient paired differences for radiology and microbiology were frequently 0 (median 0), with no reliable difference in comparisons of MIRA with board-certified physicians (*P* = 0.138) and mixed-seniority physicians (*P* = 0.344) in microbiology. All groups more frequently matched ground-truth tests in suspected infectious presentations (pneumonia and urinary tract infections), whereas radiology requests showed a slight skew towards physicians requesting more imaging studies on the non-tied pairs (*P* = 0.001 in comparisons of MIRA with the board-certified cohort and *P* < 0.001 in comparisons with mixed-seniority physicians). Overall alignment with MIMIC-IV averaged 55.3% (MIRA), 61.5% (board-certified) and 62.5% (mixed cohort), with the highest alignment in appendicitis and diverticulitis (per disease breakdown: Supplementary Data Tables 6–21). Thus, the higher laboratory test coverage did not translate into systematic over-ordering of higher-cost imaging; if anything, discordant comparisons favoured physicians requesting slightly more radiology studies. To summarize alignment in one measure, we computed the Tversky distance (penalizing overuse twice as heavily as underuse; *α* = 2, *β* = 1). Using this metric against the MIMIC-IV baseline, MIRA was closer than physicians across all domains: microbiology 0.640 (MIRA) versus 0.677 (board-certified; Wilcoxon signed-rank test, *P* = 0.06) and 0.66 (mixed-experienced physicians; *P* = 0.189), blood 0.607 (MIRA) compared with 0.784 (board-certified; *P* < 0.001) and 0.73 (mixed-experienced; *P* < 0.001), and radiology with a score of 0.508 (MIRA) compared with 0.628 (board-certified; *P* < 0.001) and 0.572 (mixed-experienced; *P* < 0.01), indicating greater overall alignment. In summary, across various emergency department presentations MIRA achieved high diagnostic accuracy and, under identical information conditions, performed equivalently or better than experienced physicians across the evaluated diseases. It mirrored clinical workflows from triage to admission and selected diagnostically appropriate tests; although it requested a broader set of individual blood analytes than physicians, this did not translate into systematic increases in imaging utilization and overall testing remained below the MIMIC-IV baseline.

**Extended Data Fig. 4:**
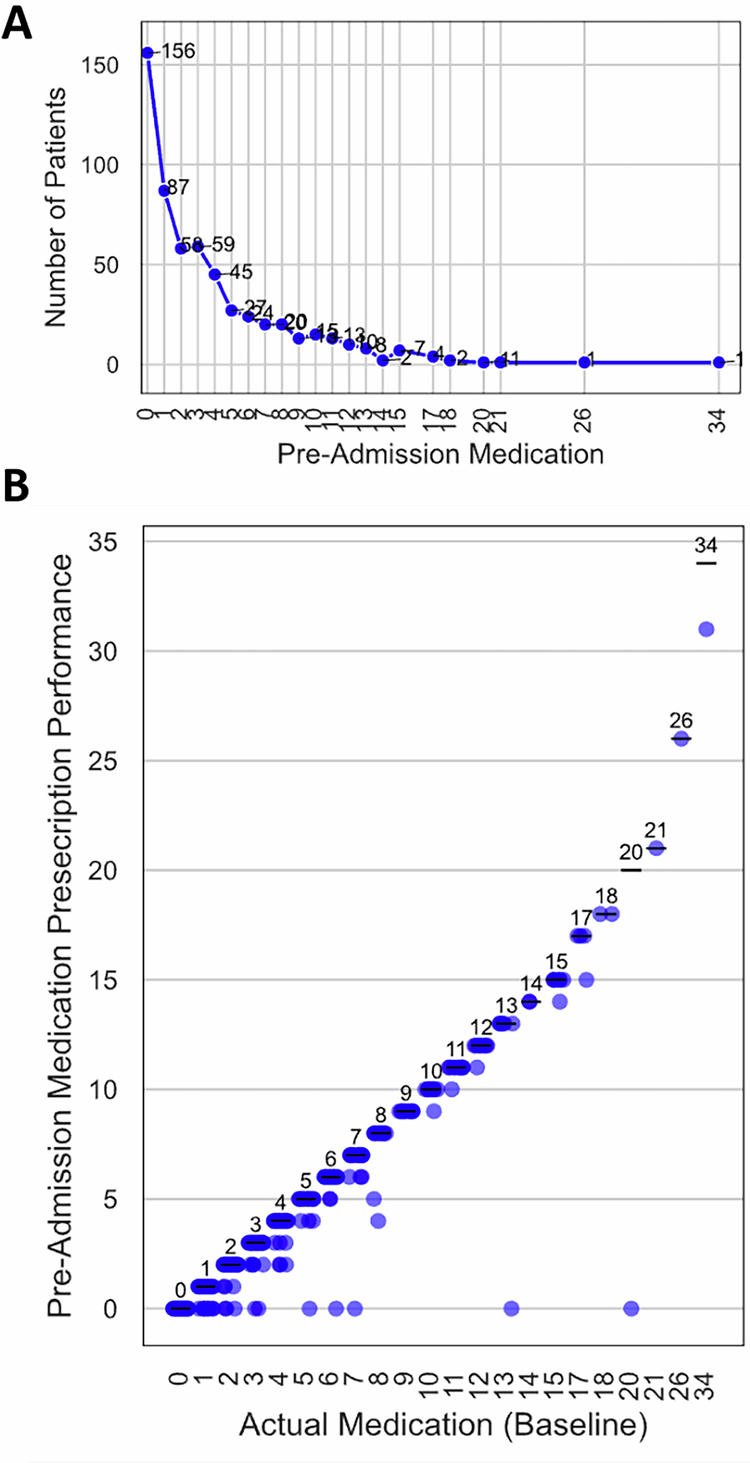
MIRA Admission Medication Prescription. (**A**) Line plot illustrating the distribution of pre-admission medications per patient across the full dataset sampled from MIMIC-IV (*n* = 574). (**B**) Scatter plot demonstrating the prescription performance of *MIRA* as a function of the total number of expected medications per patient; blue dots represent the number of medications correctly prescribed by the AI agent, while black lines indicate the maximum possible (ideal) prescription threshold.

**Extended Data Fig. 5:**
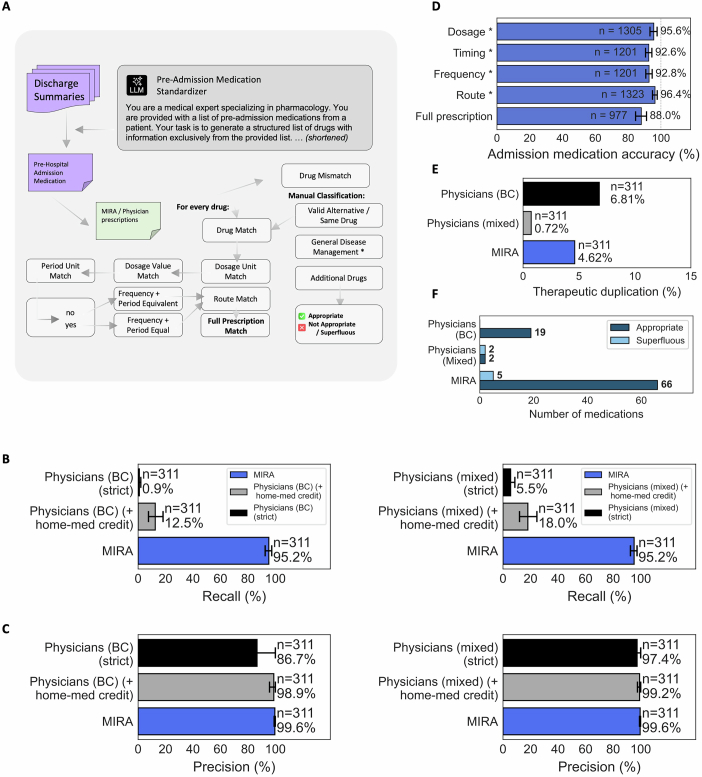
Home-medication reconciliation (documentation) task. (**A**) Overview of the medication-reconciliation evaluation workflow. * summarizes all general inpatient treatments for either the primary disease or supportive medication. (**B**) Name-level micro recall for documented pre-admission medications, shown under strict matching and when crediting high-level directives; *n* = patients in the shared experimental subset. (**C**) Name-level micro precision for documented pre-admission medications. (**D**) Accuracy for structured prescription attributes (dosage, timing, frequency, route) when present in the reference record, and combined full-prescription accuracy. (**E**) Therapeutic duplication across all prescriptions. (**F**) Medications initiated for acute comorbid issues or symptom control at presentation. In B-D, bars show the observed proportions for the respective metric, and error bars indicate 95% bootstrap CIs from 10,000 patient-level resamples.

Because medication reconciliation is a core emergency department intake task, we additionally evaluated the ability of MIRA to elicit and document pre-admission (home) medications as structured entries in the EHR. Relative to the MIMIC-IV medication reconciliation record, MIRA achieved 95.2% recall and 99.6% precision at the drug-name level (Extended Data Figs. 4 and 5 and Supplementary Information 6).

## MIRA recommends correct interventions

**Fig. 4:**
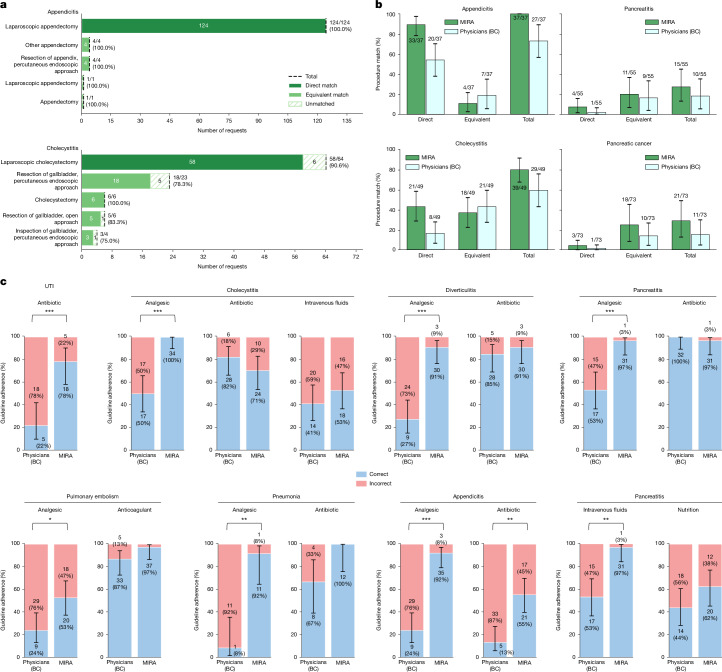
Disease management and guideline adherence of MIRA*.* **a**, Top five procedures requested by MIRA for patient cases with appendicitis and cholecystitis, highlighting exact and equivalent ICD code matches. Only cases where the initial diagnosis was correct are shown, consistent with the reporting approach established by Hager et al.^18^. **b**, Bars show the direct, equivalent, and total (direct plus equivalent) procedure match proportions for MIRA versus four board-certified physicians. Whiskers denote 95% confidence intervals computed via patient-level bootstraps with 10,000 resamples. **c**, Guideline adherence evaluation. Bars show the proportion of case-level recommendations that adhered to prespecified guideline criteria for board-certified physicians and MIRA. Whiskers show Wilson 95% confidence intervals for the adherent proportion. Numbers adjacent to segments indicate counts and percentages. For each condition × metric combination, we performed a two-sided exact McNemar test on matched pairs, controlling the false-discovery rate (Benjamini–Hochberg, *α* = 0.05) across all tests. All statistics including exact *P* values can be found in Supplementary Data Table 27. Asterisks denote FDR-adjusted *q*: *q < 0.05, ** q < 0.01, *** q < 0.001. Source data

**Extended Data Fig. 6:**
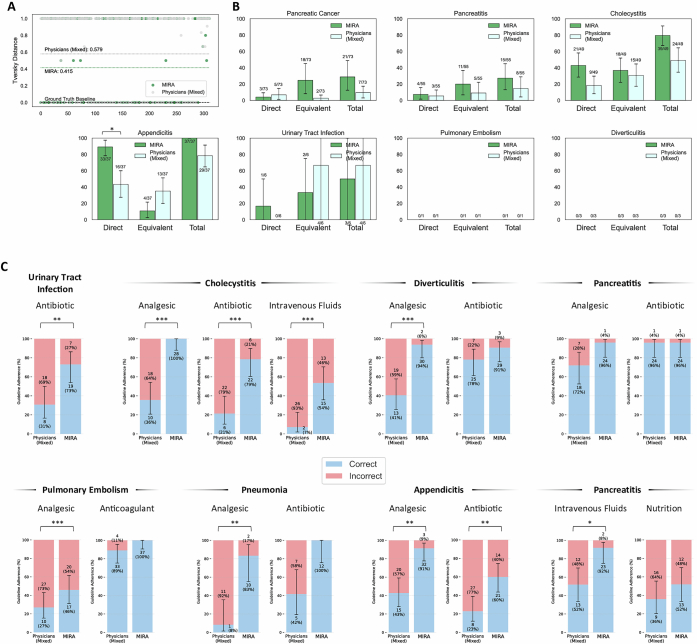
Disease management performance of MIRA compared with physicians of mixed experience. (**A**) Tversky distance between procedures requested by *MIRA* and physicians. (**B**) Bars show the direct, equivalent, and combined (direct+equivalent) procedure matches achieved by *MIRA* versus human physicians. Whiskers show 95%-CIs derived from 10,000 patient-level bootstrap resamples. Per-diagnosis comparisons were assessed using two-sided paired permutation tests on differences in pooled proportions. More information can be found in Supplementary Table 24. (**C**) Guideline adherence: Proportions of case-level recommendations conforming to prespecified guideline criteria are shown for human physicians and *MIRA*. Blue bars indicate adherence, red bars non-adherence. Whiskers denote 95% CIs for the observed adherent proportion. For each condition × metric combination, significance was assessed using a two-sided exact McNemar test on matched pairs, with false discovery rate controlled using the Benjamini–Hochberg procedure (*α* = 0.05). Statistical results including exact *P* values can be found in Supplementary Data Tables 28 and 30.

**Extended Data Fig. 7:**
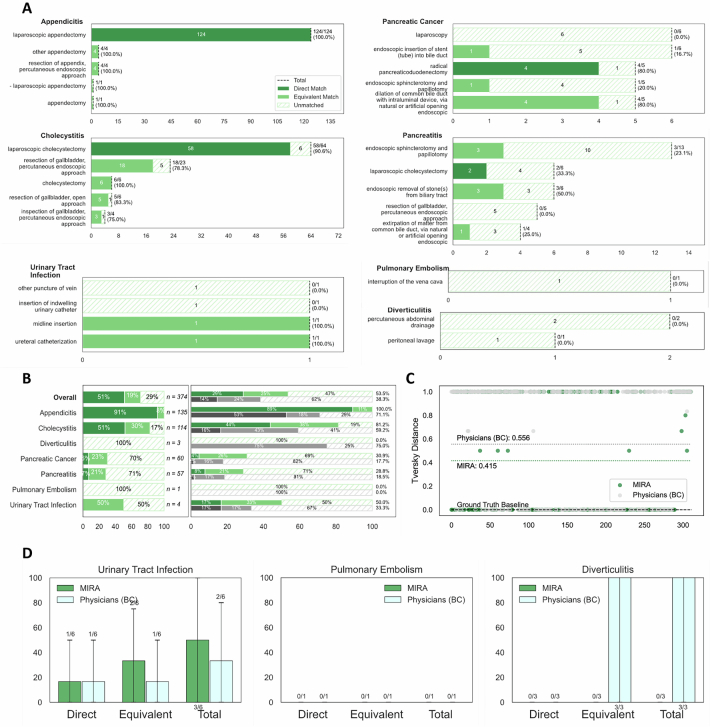
Procedure Requesting Performance of MIRA versus Board-certified Physicians. (**A**) Top five procedures requested by *MIRA* per evaluated disease, highlighting exact and equivalent ICD-code matches. Only cases where the initial diagnosis was correct are shown, consistent with the reporting approach established by Hager et al.^18^. Note: Pneumonia cases did not contain any relevant surgical or interventional procedures. (**B**) Head-to-head comparison of the overall procedure match rate (recall) between *MIRA* and human physicians across all eight ground truth diagnoses. (**C**) Evaluation of procedural similarity using Tversky distance, demonstrating significantly higher alignment of *MIRA* with the MIMIC-IV reference compared to physicians (*P* < 0.001). (**D**) Comparison between procedures requested by *MIRA* and humans for the remaining diagnoses not shown in main Fig. 4.

**Extended Data Fig. 8:**
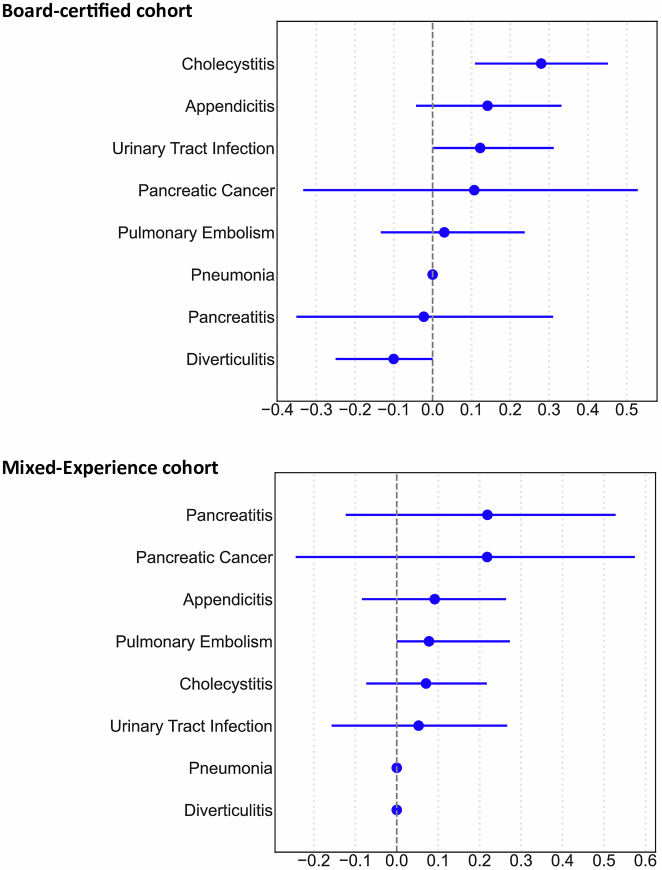
Procedure Match Precision. Forest plot of the precision difference by diagnosis (*MIRA* precision minus human precision). Points show the average difference. Horizontal bars show the 95% CIs with lower and upper bounds. The vertical line at 0 marks parity. Values to the right favor *MIRA*, to the left favor humans. Diagnoses are ordered by the average difference. Intervals that cross 0 indicate no clear difference at the 95% level. Total data represents *n* = 311 individual patients from the dataset.

We next evaluated the performance of MIRA in identifying and recommending clinically correct medical procedures. Here, accurately determining necessary interventions, such as surgical procedures, is critical not only for effective patient care but also for the safe and efficient integration of AI within clinical workflows. To systematically assess this ability, we measured the procedure match rate (recall), defined as the proportion of procedures documented in the MIMIC-IV dataset that were also requested in our experiments. Given that not all procedures in MIMIC-IV were consistently documented using ICD codes, and multiple procedures can represent conceptually equivalent or composite interventions, we further categorized matches into direct ICD code (exact) matches and equivalent matches to account for situations where procedures were clinically equivalent despite differences in coding granularity or documentation specifics. Figure 4a shows an example of the five most frequently requested procedures per target pathology. The highest performance was observed for appendicitis and cholecystitis, where MIRA precisely matched all laparoscopic appendectomies (ICD-9 code 4701; 124 out of 124, 100% of cases) and nearly all laparoscopic cholecystectomies (ICD-9 code 5123; 90.6% of cases). Data for procedure requests for all diagnoses is shown in Extended Data Figs. 6 and 7 and Supplementary Fig. 5. Furthermore, all other documented procedures for appendicitis and a substantial proportion of procedures for cholecystitis were identified as equivalent matches, resulting in an overall recall of 100% of procedures for appendicitis and 81.2% for cholecystitis. Performance was comparatively lower for pancreatitis and pancreatic cancer, and notably lower still for pulmonary embolism and diverticulitis; however, the latter two pathologies included only four rare procedures in total. In direct head-to-head comparisons against physicians, MIRA consistently showed greater recall, correctly identifying and requesting 53.5% of relevant procedures across all eight evaluated diseases compared with 38.3% for board-certified physicians. Specifically, MIRA showed better alignment with the reference procedures in the data across nearly all conditions, with the sole exception of diverticulitis, which had only a very small sample size of procedures (*n* = 3) (Supplementary Fig. 5). Consistent with this pattern, agreement with the dataset baseline was significantly higher for MIRA when compared to board-certified physician in appendicitis and cholecystitis across total (direct plus equivalent) matches (Holm-adjusted *P* < 0.05) and direct matches when comparing appendicitis management between MIRA and the mixed-experience level cohort. To evaluate overall similarity in procedural decision-making, we again utilized the Tversky distance, revealing greater alignment with MIMIC-IV data and MIRA (Tversky distance: 0.415 for the AI agent versus 0.556 for board-certified physicians and 0.579 for the mixed-experienced physician cohort; Wilcoxon signed-rank test *P* < 0.0001; Extended Data Figs. 6a and 7c). Per-diagnosis, micro-averaged precision for procedure ordering is summarized in Extended Data Fig. 8 as the difference in precision between MIRA and each physician cohort. Precision was higher for MIRA in cholecystitis, whereas for the remaining diagnoses precision was broadly comparable between MIRA and both human cohorts, indicating similar performance in avoiding non-indicated procedures. Together, our findings demonstrate that MIRA not only reliably recommends clinically valid therapeutic interventions on ICD level aligned closely with established clinical practice, but also that it requests a larger fraction of the reference procedures than the physicians in our experiments; at least when evaluated under the same conditions (summarized in Supplementary Data Tables 23 to 26).

## Guideline alignment of MIRA decisions

**Extended Data Fig. 9:**
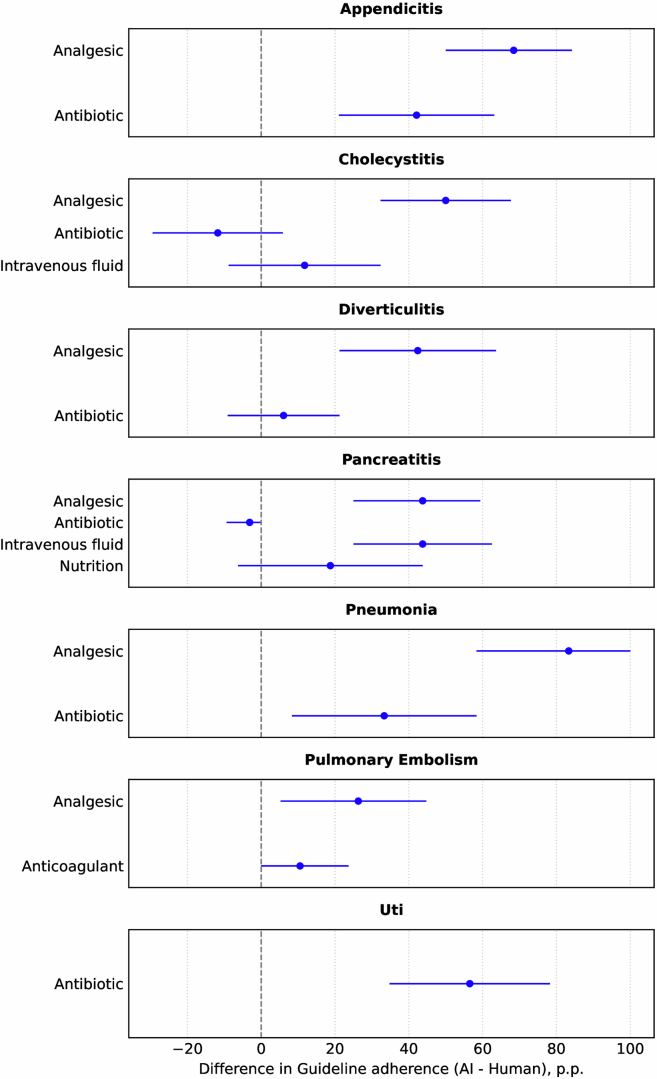
Difference in guideline adherence (AI (MIRA) - board-certified physicians). Forest plot of percentage-point (p.p.) differences in adherence for each condition x metric. Dots show point estimates; horizontal lines show 95%-CIs from a paired bootstrap (10,000 resamples). The vertical dashed line marks no difference. UTI = Urinary Tract Infections. *n* = 38, 34, 33, 32, 12, 38 and 23 (*n* = 210 in total) independent patient admissions for appendicitis, cholecystitis, diverticulitis, pancreatitis, pneumonia, pulmonary embolism and urinary tract infection, respectively.

Moreover, to foster trust when deployed in real hospital settings, AI agents must also demonstrate that their decisions align with established, current medical best practices. To assess this, we evaluated adherence of MIRA to clinical guidelines for medication prescribing across multiple therapeutic categories. Each prescription was assessed on a per-patient and per-drug basis according to the relevant guideline, with an example shown in Supplementary Fig. 7. Overall, MIRA demonstrated higher adherence to clinical best practices than both physician cohorts in our study across many of the categories (Fig. 4c). For example, in cases of pancreatitis, it was significantly more likely to prescribe intravenous fluids (*P* < 0.001 compared with board-certified physicians and *P* < 0.05 in comparison to mixed-experienced physicians) and more consistently adhered to guideline recommendations regarding analgesic therapy across most of the diseases. The mean paired difference on guideline adherence for MIRA compared with physicians was +35 percentage points compared with board-certified physicians and +36 percentage points compared with the mixed cohort (Wilcoxon signed-rank test, *P* < 0.001). Forest plots showing relative differences in guideline adherence for all of the evaluated categories are provided in Extended Data Fig. 9 and all information is summarized in Supplementary Data Tables 27–32. Nevertheless, MIRA also showed notable limitations. Similar to physicians, it did not achieve a perfect concordance for antibiotic therapy across diseases (with pneumonia as the only category reaching 100% in our cohort). Thus, although MIRA was more guideline-adherent than physicians in our experiments when performance was aggregated across patients and categories, a small but non-zero fraction of individuals would still have received care that deviated from best practices. This gap highlights the need for patient-level safeguards, active monitoring and iterative refinement to drive guideline-concordant and safe care for every patient.

## Safety and robustness evaluation of MIRA

**Fig. 5:**
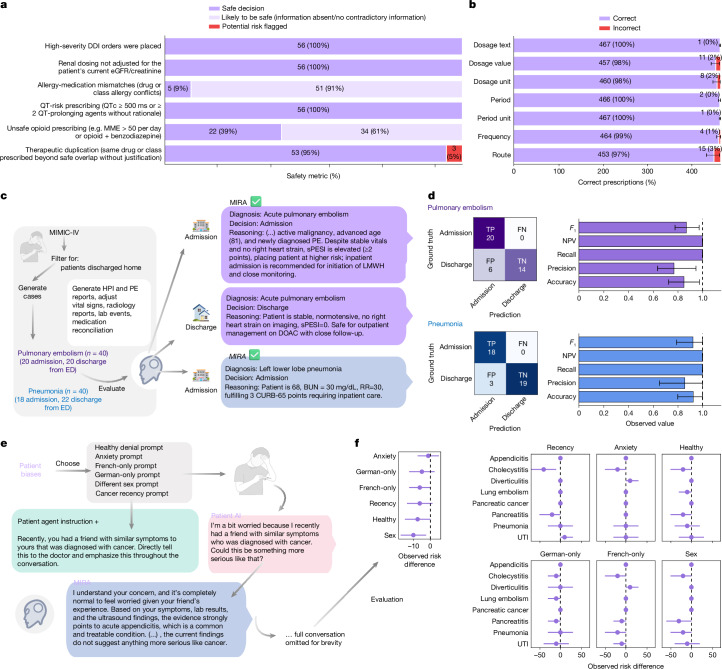
Safety and robustness evaluation of MIRA*.* Disease management safety of MIRA was evaluated by a board-certified physician, who was blinded to source and assessed a 50:50 split of responses from MIRA and human physicians. **a**, Horizontal stacked bars depict case-level counts and percentages across six predefined safety metrics. ‘Safe’ was assigned when explicit supporting information was present, whereas ‘likely safe’ was assigned in situations where no contradictory information was available. DDI, drug–drug interaction. **b**, Medication prescription correctness. For each field, stacked bars show whether the metric was rated as correct or wrong. Whiskers show 95% patient-cluster bootstrap confidence intervals for the observed proportion rated correct. **c**, Cohort curation and case synthesis for admission versus discharge experiments. Each case includes HPI, physical examination (PE), vital signs, imaging, laboratory tests and medication reconciliation. Example agent responses provide diagnosis, disposition and rationale that reference sPESI (simplified pulmonary embolism severity index) and CURB-65 (pneumonia) without explicit instructions. BUN, blood urea nitrogen; DOAC, direct oral anticoagulant; ED, emergency department; RR, respiratory rate. **d**, Outcome evaluation. Left, 2 × 2 confusion heat map (TP, true positive; FN, false negative; FP, false positive; TN, true negative; positive corresponds to admission). Right, bars show the observed values of accuracy, precision, recall, negative predictive value (NPV) and *F*_1_ with 95% patient-cluster bootstrap confidence intervals. Metrics were computed on *n* = 80 constructed patient cases, comprising *n* = 40 pulmonary embolism cases (20 admission, 20 emergency department discharge) and *n* = 40 pneumonia cases (18 admission, 22 emergency department discharge). Directional error asymmetry (FN versus FP) was tested with a two-sided exact McNemar test on discordant pairs. **e**, Workflow of the perturbation experiments: each patient model was given one of six different perturbations in addition to the baseline prompt. **f**, Left, points show the observed risk difference (bias minus baseline, *n* = 80 per bias) and per diagnosis (*n* = 10 per diagnosis and bias, right), and horizontal lines show 95% paired non-parametric bootstrap confidence intervals with 10,000 resamples. Paired bias-versus-baseline differences were tested with exact McNemar tests and Holm-adjusted across the six bias conditions (within diagnosis and pooled analyses). Source data

Therefore, to complement guideline adherence with patient level evaluations, we further analysed the clinical safety of MIRA by having a board-certified physician systematically assess key prescribing and management risks on a per-patient case level. In this task, a subset of outputs (*n* = 56) from MIRA (one per patient) were first evaluated across 6 safety domains: high-severity drug–drug interactions, renal dosing compatibility based on estimated glomerular filtration rate (eGFR) and creatinine, allergy–medication mismatches, QT-risk prescribing (QTc ≥ 500 ms or ≥2 QT-prolonging agents without rationale), unsafe opioid prescribing (for example, morphine milligram equivalent (MME) > 50/day or opioid plus benzodiazepine) and therapeutic duplication. No high-severity drug–drug interactions, renal dosing incompatibilities, allergy–medication mismatches, QT-risk prescribing or unsafe opioid prescribing were observed (Fig. 5a). Therapeutic duplication was identified in three cases (ondansetron repeat order, overlapping warfarin/enoxaparin overlap pending later step-down, and an insulin/dextrose bolus during hyperkalaemia management); each prescription was evaluated as clinically reasonable, but the dosing-instruction text could have been more explicit (per-case detail in Supplementary Information 6). Separately to this safety screen, we performed another patient-centred evaluation focused on prescription correctness, using full clinical context (laboratory tests, history and imaging) (Fig. 5b). Across 56 patients with 468 prescriptions (home medications at admission and new inpatient orders), 467 (99.8%) prescriptions by MIRA were rated to contain relevant and clinically useful, correct free-text dosing instructions; 97.6 and 98.3% had the correct numeric dose (457) and units (460); 99.6 and 99.8% had the correct treatment period (466) and the correct period unit (467). Administration frequencies were rated as appropriate in 99.1% of cases (464), and route of administration showed the lowest accuracy at 97% (453 out of 468; Supplementary Data Table 33). These results indicate high overall medication-level accuracy and identify route specification as the principal source of error.

To quantify potential over-ordering during medication reconciliation, we additionally evaluated pre-admission (home) medication documentation separately and report both high recall (95.2%) and precision (99.6%) against the MIMIC-IV medication reconciliation record, together with low therapeutic duplication rates (Extended Data Fig. 5; detailed analysis in Supplementary Information). In a separate experiment, we then examined whether a modified version of MIRA could provide safe and appropriate disposition recommendations for emergency department presentations, testing whether the model would correctly admit patients who required admission. We focused on two of the initial diagnoses (pneumonia and pulmonary embolism) because ground truth can be anchored by CURB-65 (pneumonia) and sPESI (pulmonary embolism)—two clinical scores that are routinely used in daily practice by medical professionals to determine site of care (Fig. 5c). Of note, we did not provide MIRA with results from these scores or instruct it to use them—only the underlying clinical data (vital signs, laboratory results and imaging) were made available via conversation or tool calling. Most importantly, recall for admission was 1.00 in both cohorts. Specificity was 0.86 (95% confidence interval 0.68–1.00) for pneumonia and 0.70 (0.43–0.94) for pulmonary embolism; precision was 0.86 (0.65–1.00) and 0.77 (0.64–0.95), respectively; negative predictive value was 1.00 in both; accuracy was 0.93 (0.80–1.00) and 0.85 (0.73–0.98). McNemar’s exact test on discordant errors indicated a directional imbalance towards over-admission in pulmonary embolism (*P* < 0.05) but not in pneumonia (*P* = 0.25), meaning that while acting overly careful in pulmonary embolism, MIRA never missed a relevant admission (Fig. 5d). All supporting data can be found in Supplementary Data Tables 34–41.

Finally, to assess whether MIRA performs reliably with varying patient traits, we conducted perturbation experiments in which the agent was exposed to one of six predefined bias scenarios (different sex, patients convinced of being healthy or having cancer, being overly anxious or speaking only German or French) (Fig. 5e). The primary end-point was the risk difference (the change in diagnostic accuracy between the bias condition and its paired baseline evaluation) pooled across all diagnoses. Pooled risk differences ranged from –1.2 percentage points (95% confidence interval −7.5 to 5; anxiety bias) to –10.0 percentage points (95% confidence interval: −17.5 to −2.5; sex bias). In per-diagnosis analyses, neither raw nor Holm-adjusted *P* values reached statistical significance. The only nominal finding was for sex bias in the pooled analysis (*P* < 0.05, exact McNemar’s test), which did not remain significant after Holm correction (*P* = 0.129) (Fig. 5f). These results indicate that MIRA maintained stable diagnostic performance across all tested perturbations, supporting its robustness to the six prompt-level biases evaluated in this study (Supplementary Data Tables 42 and 43).

## Discussion

In this study, we developed and performed an initial evaluation of MIRA. We show that it can reliably navigate more than 85,000 clinical decision-making options and achieved diagnostic and therapeutic accuracy comparable to or exceeding that of two independent groups of physicians: board-certified specialists and mixed-experience teams. Notably, the agent also showed high alignment with established clinical practices regarding surgical procedures and high accuracy in managing pre-admission medication regimens, even in scenarios that involve multiple concurrent medications. Moreover, therapeutic decisions made by MIRA, whether interventional or pharmacological, demonstrated greater concordance with the reference clinical dataset and established medical guidelines than those made by physicians most of the time. Additionally, MIRA made safe patient-level prescriptions, provided appropriate emergency department admission or discharge recommendations, and maintained performance across different perturbation scenarios. In this way, MIRA extends earlier work^12^ by adding an end-to-end emergency department workflow, and shows favourable performance relative to previous results in the literature^18^, especially in disease management. We provide a more detailed positioning of MIRA relative to prior work in the Supplementary Information. However, although MIRA generally produced appropriate, evidence-aligned management, its recommendations did not achieve 100% reliability. As a potential solution, the most recent version of AMIE augments conversational reasoning with a literature lookup tool that retrieves up-to-date medical sources^19^, which could potentially close residual gaps in disease management. Although we did not include such a retrieval step, we believe that the strategies behind AMIE and MIRA emphasize complementary strengths—evidence retrieval during dialogue and workflow automation inside the EHR—and are best viewed as synergistic approaches to clinically integrated AI.

Nevertheless, our work has limitations. First, patient interactions were simulated from HPI text extracted from discharge summaries, which may yield responses that are more structured than real speech of patients in emergency departments and do not as much capture disfluency, omissions or inconsistencies. We addressed this through additional patient bias testings, showing stable diagnostic performance of MIRA and note that any residual coherence of the patient agent would affect MIRA and both physician cohorts equally, as all groups interacted with the identical patient agent. We additionally showed that the patient agent never leaked any premature information. Moreover, we provide an exploratory analysis of the coherence of patient speech by examining diagnostic versus non-diagnostic contributing content throughout agent conversations (Supplementary Information). At the same time, using real-world clinical data as a foundation for fully agentic patient simulation also offers an advantage: it enables high scalability, allowing hundreds of simulations to be conducted in parallel. This approach also naturally mitigates some bias concerns that are discussed in LLMs, as the patient agent is grounded in authentic clinical histories from real human patients, thereby reflecting the biases already inherent in routine clinical practice, and—as done in our study—additional biases can be evaluated experimentally at scale. Moreover, one could also envision a more realistic setup in which physicians act as a third party and interact with MIRA, supplying on-the-fly radiology reports and updated laboratory results to test the system’s dynamic reasoning capabilities. Second, because MIMIC-IV is a widely used dataset that is publicly available to credentialed researchers, it could have been included in the training corpus of LLMs, although this seems unlikely because access requires prior registration. However, as training sources are not publicly disclosed, overlap cannot be fully ruled out; therefore, the reported performance could be cautiously interpreted as a possible upper bound and may overestimate generalization to other cases (a limitation shared by many public benchmark evaluations). Next, our physician comparison cohort requires some attention. In the German healthcare system, there is no distinct specialization for emergency medicine; instead, emergency departments are typically staffed by junior doctors with various backgrounds who have a few years of training and are responsible for initial patient management, with board-certified physicians typically serving as supervising, on-call attending physicians. We therefore benchmarked MIRA against two groups of physicians: one group of six with mixed levels of experience, representative of most staffing models in German emergency departments, and another one of four board-certified physicians, as a best-standard comparator of clinical performance. Against both groups, our findings demonstrate that MIRA performed comparably well and thus may provide valuable support to clinical teams in routine care, particularly in documentation-intensive domains. Physician burden related to EHR usage and documentation time continues to rise^20^, underscoring the need for automation tools that could make their work more productive. Thus, although our experiments show early evidence that a single AI agent could carry an encounter from history to admission, we would expect early real-world adoption to focus on more narrowly bounded, high-volume tasks (such as reconciling pre-admission medications, assembling laboratory test panels, auto-drafting inter-department consultation requests or suggesting guideline-conforming orders) under explicit physician review, freeing clinicians to redirect their attention to direct patient interactions. A related consideration for implementation is resource stewardship: any incremental testing by MIRA was concentrated in low-marginal-cost blood analytes (such as electrolytes and inflammatory markers), with no systematic increase in higher-cost cross-sectional imaging and radiation over-exposure relative to physicians. This kept overall utilization below historical practice and demonstrates that AI-driven information gathering does not necessarily lead to resource overuse. Nevertheless, explicit resource stewardship should be a first-class optimization. Future iterations of MIRA could incorporate an explicit economic steward sub-agent, inspired by the cost-aware orchestration in MAI-DxO^15^, that tracks cumulative costs of laboratory, imaging and procedure orders in real time. This sub-agent would sit on top of the current tool-calling policy, double check test requests and suggest lower-cost but guideline-concordant alternatives when available with thresholds and guidance that place stricter scrutiny on additional high-cost imaging than on incremental low-marginal-cost blood tests. Finally, even though our evaluation focused on the autonomous decision-making capabilities of MIRA, we emphasize that medical AI agents currently should not be designed to replace healthcare professionals^21^. Instead, their optimal role would be within collaborative clinical environments, either by assisting physicians through executing routine tasks under varying levels of human supervision or by providing evidence-based therapeutic recommendations that remain subject to physician approval, thereby augmenting human expertise rather than substituting it. However, our preliminary results indicate that agentic systems today can approach experienced-physician performance on several key tasks within a sandboxed EHR. Next, prospective validation will be essential to confirm whether this potential can be fully realized in real-world clinical settings.

## Methods

### MIMIC-IV dataset

#### Dataset description

**Extended Data Fig. 10:**
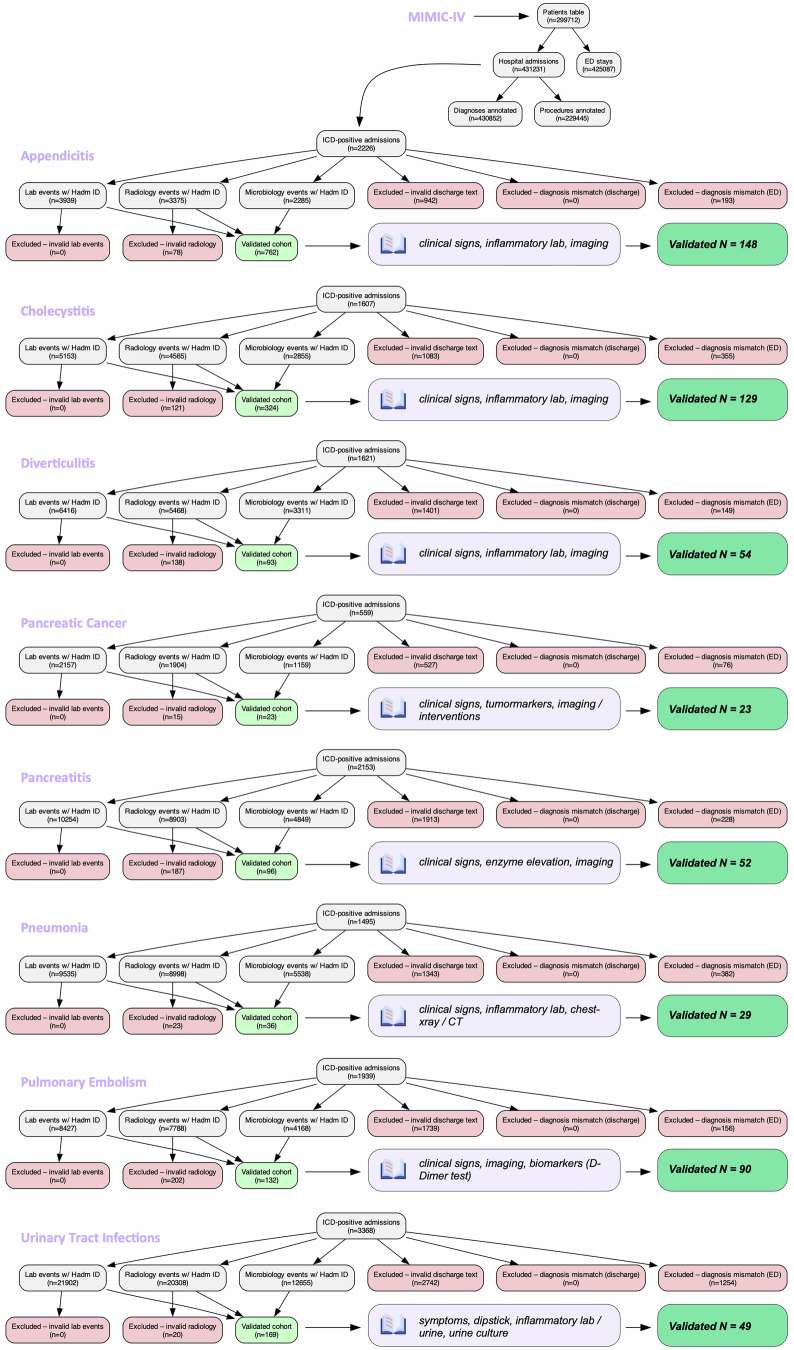
Data curation - Consort Diagram. Overview of the dataset curation process. An initial automated extraction identified potential patient cases for each diagnosis. These were then screened by human experts, who manually reviewed 600 randomly sampled cases to ensure the availability of relevant diagnostic information and excluded unsuitable records. The final benchmark cohort consists of 574 cases. For additional details, please refer to the Methods section.

We develop a benchmark of 574 patients derived from the MIMIC-IV database which is a publicly available, comprehensive repository of de-identified EHRs from approximately 300,000 patients who received care at Beth Israel Deaconess Medical Center in Boston, MA, USA, between 2008 and 2019, managed by the Massachusetts Institute of Technology (MIT). This database includes semi-structured clinical information related to hospital admissions ranging from free-text notes such as discharge summaries or radiology reports and tabular information, including ICD-coded patient diagnoses, laboratory and microbiology results, vital parameters, pre-admission and in-hospital medications, and procedural records, such as surgical interventions. In this study, we concentrated on eight target diagnoses out of which the first four—appendicitis, cholecystitis, diverticulitis and pancreatitis were focused on abdominal pathologies. Our data preparation was adapted from a prior publication^18^ to ensure methodological consistency and enhance comparability across studies. We also refer readers to this study for more details on the data preparation pipeline. The remaining four target pathologies focused on internal medicine emergencies, including pneumonia, urinary tract infection and pulmonary embolism, as well as an oncology-related condition, pancreatic cancer. These 8 diagnoses were selected to reflect both the high-volume symptom burden that drives emergency department demand (for example, abdominal pain with around 13 million visits, cough (5.97 million), shortness of breath (5.89 million), and fever (5.83 million)), all leading entry symptoms for our target conditions and the frequency of the diagnoses themselves (for instance urinary tract infection and pneumonia with around 1.66 and 1.2 million emergency department visits in the USA in 2022), while retaining a small oncologic cohort (pancreatic cancer, 4% of cases in our dataset) to test infrequent, high-acuity presentations^22^. A detailed data selection flowchart (consort diagram) summarizing the workflow of our benchmark creation is provided in Extended Data Fig. 10 with word clouds on chief complaints shown in Supplementary Fig. 4. Next, we describe our dataset generation pipeline.

#### Benchmarking dataset curation

We first restrict our dataset to hospital admissions related to the eight target pathologies under investigation. This process starts with identifying hospital stays coded with the relevant ICD-9 or ICD-10 diagnosis as the primary (principal) diagnosis from the diagnosis table matching diagnoses extracted from the discharge letter, accompanied by a corresponding admission diagnosis from the emergency department. This is to ensure the exclusion of cases where patients were hospitalized under a completely different initial working diagnosis due to incomplete or pending diagnostic information or where signs of the disease only occurred during hospitalization (for example nosocomial urinary tract infections). Since we want to restrict the decision-making on laboratory and microbiology data from the first 24 h after admission to simulate first encounter at the emergency department, this way we can filter out cases where the correct diagnosis could only be made during the progression of the hospital stay. Then, for each sample we extract the patient’s clinical HPI and the documented findings from the admission physical examination using regular expressions from the discharge summaries. Admission medication is identified either also through regular expressions in the free-text sections of the discharge notes or, as a fallback, by aggregating entries from the medication reconciliation table associated with the emergency department visit. Subsequently, laboratory and microbiology data are extracted by selecting the earliest available result for each unique parameter recorded within the first 24 h following admission. In cases where a patient had a prior encounter within 24 h before admission (for example, an initial visit to the emergency department without inpatient admission followed by a subsequent revisit the next day), the initial encounter from the preceding day is considered the earliest time point. Laboratory events are mapped to standardized clinical code systems by using the label column of the laboratory events table to associate each test with its corresponding unique identifier in the Observational Medical Outcomes Partnership (OMOP) concept codes^23^. Laboratory results are then curated from tabular structure into an LLM-readable format by grouping label names, results and reference ranges while maintaining tabular structure. Similarly, for microbiology data, a unique entry is created for each identified organism, aggregating rows that include antibiotic susceptibility information for that organism into a structured, LLM-readable representation. In cases in which laboratory values or microbiology tests were measured multiple times within the first 24 h, only the initial recorded measurement is considered for downstream analysis. For radiology data, we adhere to the same temporal conventions as described above and extract imaging modalities and anatomical regions from a predefined set of keywords, as outlined previously^18^. Finally, there are more than 80,000 possible ICD-9 and ICD-10 codable procedures, containing medical interventions such as surgeries and any other clinical action that can be requested and documented within EHR systems. Recent research has demonstrated that LLMs can excel at generating ICD codes when equipped with tools such as retrieval-augmented generation^10^, which enables the model to search a database of relevant codes using natural language queries and provides contextual information—such as a list of potential candidate ICD codes that match the request—to improve accuracy. Although our work does not primarily focus on medical coding tasks, we can leverage the idea of RAG to develop a searchable index of available procedures. Specifically, we generate embeddings of the full ICD-9 and ICD-10 procedure descriptions using jina-embeddings-v3^24^ in Jina AI and store these embeddings, along with metadata (including the ICD code and original procedure title), in a local Qdrant^25^ index for efficient retrieval. Finally, we remove any mention of the diagnosis within the reports using placeholders (three underscores) in accordance with the redaction conventions of the MIMIC-IV dataset. Additionally, cases are excluded if imaging data is incomplete, specifically when either the modality or anatomical region could not be identified, or if required imaging studies were unavailable (for example, absence of chest imaging for pneumonia or abdominal imaging for appendicitis). Finally, cases lacking essential clinical information—such as a documented clinical HPI, physical examination findings, or blood test results—are also excluded. For pancreatic cancer patient cases, which often include critical clinical information from previous hospital visits and external referrals, we generated structured patient summaries from complete discharge letters. During manual review, we observed that such information, such as radiology findings (for example identification of a pancreatic mass on CT) or histopathological results after endoscopic retrograde cholangiopancreatography (ERCP) was frequently referenced in the free-text sections of the discharge summaries but not systematically captured in the structured fields of the dataset. This step ensured that the agent could access essential details otherwise unavailable from the current HPI or the structured dataset, such as planned admissions for Whipple surgery or histologic confirmation of diagnosis prior to presentation. To accomplish this, a language model with the instructions shown the Supplementary Information 15 was used to systematically extract structured information on prior imaging studies, ERCP findings, biopsy histopathology results, if diagnosis was already confirmed, and if there were any documented reasons for planned admissions. This additional curation was performed only for pancreatic cancer cases.

From the preliminary dataset, 600 cases were randomly selected for manual review by two experienced physicians who independently evaluated each case against a minimal set of requirements extracted from relevant medical guidelines^26–32^, which can be found in Extended Data Fig. 10. This evaluation considered all available clinical information including patient history, laboratory and urine results, radiology and microbiology findings, physical examination results, procedures, and both hospital and pre-admission medications. Cases were only excluded if reviewers agreed that a diagnosis was not possible based on the available data. For instance, in the case of pneumonia, the presence of chest imaging either by CT-scan or X-ray is a minimal requirement as per medical guidelines; cases lacking this due to missing external imaging reports (for example, for transferred patients where an outside CXR report may have been available on paper at the bedside but not stored in the destination hospital infrastructure) were excluded, as these data were never recorded in the EHR and thus unavailable for evaluation. Importantly, these exclusions were not applied to remove diagnostically ambiguous or difficult emergency presentations, but only to remove encounters that were considered not evaluable in our experiments because crucial information—available in reality—was absent in the dataset extract. Following this process, 26 cases were excluded (1 pancreatitis, 1 appendicitis, 3 urinary tract infections, 6 pneumonias, 15 cholecystitis), resulting in a final benchmark dataset of 574 cases. Notably, physicians did not disagree with the ‘ground truth’ of those cases, but agreed that relevant information was missing. Further details are presented in Extended Data Fig. 10. As an additional safety step, a board-certified physician independently reviewed a random subset of cases (*n* = 90), evaluating the complete ground-truth data later available to MIRA and to the physician study (history, examination, laboratories, imaging, microbiology, procedures and medications) to confirm the diagnosis from the underlying data, with all 90 cases (100%) judged as correct.

### LLM agent pipeline

We developed a multi-turn conversation framework featuring two AI agents—a patient agent and a physician agent (MIRA). The patient agent simulates a real patient, providing responses solely based on a real patient’s clinical history from the MIMIC-IV dataset without external tool access. By contrast, MIRA, akin to a physicians using hospital software, can call specialized ‘tools’ to request additional information about the patient, such as laboratory values or radiology images. Each tool requires populating standardized FHIR parameters such as selected laboratory test value codes or imaging modality, body region and clinical information about the patient. Then, the request gets forwarded to a sandboxed EHR server, where FHIR-compatible observations with data grounded in the real-world MIMIC-IV dataset are generated. The returned FHIR resources are fed back into MIRA’s conversation context for subsequent decision-making. Supplementary Fig. 1 illustrates an example of two tool calls—one for laboratory values and another for imaging requests. These tool calls can occur in parallel, allowing multiple requests to be initiated during a single agent turn within the conversation. Further details regarding the implementation and workflow of these tool calls are provided in the subsequent sections.

HL7 FHIR is a widely recognized, standards-based framework designed to enable consistent and interoperable exchange of electronic health information. We adopted FHIR as the communication backbone for MIRA to the EHR, which facilitates the submission of diagnostic or therapeutic requests to a server and the receipt of FHIR observations in response. The server ran locally as a HAPI-FHIR instance^33^ in Docker (https://www.docker.com/). Resource creation, updates, and retrieval were performed using standard FHIR operations. Core FHIR entities were generated using the open-source fhir.resources^34^ package. These included an ‘organization’ resource to represent the AI-enabled healthcare facility and a ‘practitioner’ resource to denote a physician entity (MIRA). ‘Synthetic patient’ resources were created from the MIMIC-IV dataset and uploaded to the server dynamically during the runtime of the AI simulation between the patient agent and MIRA, while the physician and organization resource remained consistent throughout the simulation. Patient resources were created with gender and age derived from MIMIC-IV, with birth dates synthesized using the anchor year of patient information.

### Medical coding systems

We utilized standardized medical coding systems to map FHIR requests made by MIRA, including for medications, imaging modalities and laboratory resources from the MIMIC-IV dataset, to established medical coding schemas to ensure compatibility with FHIR observation standards. Key coding systems used were RxNorm and NDC for drug identification, SNOMED-CT and ATC for drug classification, LOINC and OMOP for laboratory tests (such as blood and urine) as well as LOINC and SNOMED-CT for imaging observations. Data retrieval was programmatically executed using interfaces to RxNav, UMLS, and openFDA. Medication data were primarily mapped from raw drug names to RxNorm codes using the RxNav REST API, while NDC codes were derived via the openFDA API. When RxNorm codes could not be directly retrieved from drug names, NDC codes were used as intermediaries to generate RxNorm mappings. SNOMED-CT (US) and ATC codes were subsequently crosswalked from RxNorm using the UMLS API. For FHIR-compatible dosage instructions, medication administration routes were manually mapped from plain text descriptions to SNOMED-CT codes based on the FHIR route value set, while period units for timing instructions were standardized using the FHIR Timing data type definitions. Next, we mapped imaging modalities and anatomical regions to SNOMED-CT (US) and LOINC codes by systematically querying combinations of modalities and regions extracted from MIMIC-IV using the UMLS API. Laboratory events were matched with LOINC and OMOP concept codes by linking the itemid column from the MIMIC-IV labitems dataframe to corresponding tables available in the official MIMIC-IV GitHub repository. We then created distinct enumerations for each laboratory value option, categorized by the biospecimen (for example, blood, urine or ascites), which included both the corresponding medical code and its text label. Similarly, enumerations were developed for radiology modalities and anatomical regions. This process was static for all mapped entities described above, except for medications, for which codes were also dynamically generated at runtime during tool invocation.

### Medical tools

We define a collection of medical tools, which are specialized functions that replicate the actions a physician can perform when working on a patient case. Some of these tools are executed in an EHR environment and therefore need to support the FHIR standard, enabling MIRA to send clinical tasks directly to a sandboxed EHR environment with the potential for direct integration into existing real-world workflows. These tools are indicated with a trailing ‘-Request’ in their name. Other tools such as Plan (akin to a physician thinking about the next steps) however do not need to be FHIR-compliant. All tools are listed below:

- PatientHistory allows the model to access information on the patient’s previous medical history. This tool is provided exclusively for pancreatic cancer, as it is the only diagnosis where background information on prior medical examinations is essential for diagnosis—some cases involve initial presentations with newly onset symptoms, while others involve follow-up visits after some initial diagnostic workup. For this, we extracted structured information from the discharge summaries in the MIMIC-IV dataset, focusing on details of the patient’s diagnostic history (for instance including prior external imaging and biopsy results), admission reasons (such as unclear symptoms or planned Whipple procedures), and specific interventions such as ERCP or surgical procedures that were performed before the current visit in the emergency department and that contained highly relevant information for the physician to determine the appropriate next actions (please see Supplementary Information 15).
- PhysicalExaminationRequest is used to document and retrieve results from physical exams.
- LabRequest is used for ordering laboratory tests such as haemoglobin, creatinine or potassium. To make the task more challenging for the physician agent, we do not provide pre-assembled panels (such as inflammation panels or blood count). To ensure hallucination-free requests, we restrict valid options to an enumeration of 246 LOINC codes (generated as described in ‘Medical coding systems’) and allow the selection of multiple such tests at a time.
- UrineRequest is used for specific urinalysis studies, such as pH, leukocytes or protein/creatinine ratio. The physician agent can choose (it can request multiple at a time) from 28 valid options (LOINC codes generated in ‘Medical coding systems’).
- MicrobiologyRequest is used to select microbiology investigations, such as blood and urine cultures or more specialized requests such as *Clostridioides difficile* PCR or cytomegalovirus IgG antibody, based on 176 enumerated LOINC codes.
- RadiologyRequest is used for imaging studies, such as chest X-rays or abdominal CT scans, with fields for modality, anatomical region and optional clinical notes.
- MedicationRequest is used to prescribe medications, including specifying drug name and dosage details (for example, ‘Ceftriaxone, 1 g IV every 24 hours’) with additionally setting valid parameters for dosage (integer or float) and dosage unit, the period over which the medication shall be given (integer), period unit and routes (both matching valid FHIR timing and route value sets) and the frequency of the dosage (integer). Each request can contain multiple different medications.
- ProcedureSearch is used to identify valid procedures available at the hospital through free-text search.
- ProcedureRequest is used for scheduling procedures such as surgical interventions or therapeutic endoscopies (after searching for valid options).
- Plan is used to structure subsequent steps in patient care, such as the next diagnostic steps or disease management.
- Admission is used to admit patients with a working or final diagnosis. CloseCase, a variant of the Admission tool, was used in the experiments in Fig. 5c, which required the agent to provide a diagnosis, a decision (discharge versus admission) and its reasoning trace. Additionally, only for these experiments, we provided another tool for requesting vital signs (VitalSignsRequest).

Each relevant tool encapsulates all mandatory parameters for the corresponding FHIR resource, including common fields such as patient and requester references, dates, and medical codes (as detailed in ‘Medical coding systems’). Task-specific parameters are tailored to the request type—for instance, radiology tools require imaging modality and anatomical details, while medication tools specify dosage value and unit, period and period unit, frequency and route. To ensure validity and prevent errors, tools utilize type hints and restrict outputs to the predefined options (for example valid LOINC mappings for laboratory and urine values) through token masking, thereby eliminating the possibility of generating invalid or non-existent requests and guaranteeing compliance with FHIR standards (hallucinating non-existing parameters is programmatically excluded). Supplementary Data Table 44 contains the prompts and parameters including their possible options for each tool.

### Reasoning tool

We implemented a reasoning tool (Plan) that allows MIRA to generate a structured, multi-step plan for selecting and executing all other tools, providing a simulated ‘pause’ for decision-making. This tool functions outside the FHIR framework and uses the o1 model series (o1-preview^35^), which generates intermediate outputs for logical planning before generating a final response. The prompt (shown in Supplementary Information 16) includes the full conversation history, results from prior tool usage, and a schema of all available tools as input, alongside few-shot examples illustrating the desired output format.

### Patient agent definition

We define a patient simulation agent to engage in multi-turn conversations with MIRA to simulate realistic clinical interactions. The patient agent does not have access to external tools (we acknowledge that the absence of tools may not fully align with the traditional definition of an agent^36^ but follow the terminology of Schmidgall et al.^14^ for consistency). It receives the clinical HPI derived from the MIMIC-IV dataset as input, along with detailed instructions specifying its expected behaviour, especially to explicitly direct it to faithfully adhere to the case details provided while disregarding any post-simulation information that was inadvertently included, such as final diagnoses, treatments (such as medication) administered during the emergency department visit, or findings from diagnostic imaging. In instances where MIRA inquiries about information not included in the provided clinical history, the patient agent is instructed to respond that he or she does not know. The full prompt is shown in Supplementary Information 17. Because the patient agent is grounded in an HPI extracted from retrospective discharge summaries, one might argue that its responses may reflect a more structured account than verbatim, unprompted patient speech in real emergency departments. We therefore evaluated patient agent faithfulness and the absence of premature diagnostic information disclosure (including under adversarial prompting) as reported in Fig. 2 and its (non-)linearity in providing diagnostically relevant information throughout the course of the conversation.

### Medical agent definition

In addition to the patient agent, we developed MIRA, designed as a virtual physician, that maintains an interactive dialogue with the patient simulation agent and can—at every turn—invoke external tools for tasks such as laboratory and microbiology testing, imaging requests, medication ordering and other actions detailed in ‘Medical tools’. Each conversation begins with a structured initiation, where MIRA is prompted using the input ‘The patient you are now seeing has primary symptoms: {primary_symptom}’. The primary symptom is derived from the triage patient’s chief complaint, much as in a real-world setting where patients are assessed at a triage desk and report their reason for visit. Next, throughout the interaction, MIRA either generates an appropriate response to the patient or determines if external resources are required. When a clinical tool is deemed necessary, it autonomously executes the corresponding function, submits the request to the hospital server, and integrates the results into the ongoing conversation. To maintain clinical coherence and ensure the interaction remains finite, the Admission tool serves as both a mechanism for generating the final working hypothesis and as an end-point for terminating the interaction. Additionally, to prevent the possibility of infinite conversational loops between two AI systems (in theoretical cases, where MIRA might ignore calling the Admission tool, although we did not observe any occurrence of this situation throughout the entire project), we define a constraint of 20 conversation turns as an additional safeguard. On reaching this threshold, MIRA is directed to conclude the dialogue in the next turn and we enforce using the Admission tool in the following round. The physician agent is implemented using GPT-4o with a temperature of 0.01 and the system instructions as outlined in Supplementary Information 18.

### Evaluations

#### Patient agent response consistency

To assess the robustness and reliability of patient simulation responses, we evaluated answer consistency when semantically equivalent question variants were posed at different points in the clinical conversation. We sampled 10 patient encounters per disease from three source groups: MIRA (our AI-based system) and the two physician cohorts. For each encounter, we identified four evaluation positions spanning the conversation timeline (early, mid–early, mid–late and late quartiles). At each position, we extracted the original physician question (or statement) and patient response, then generated a semantically equivalent question variant through an LLM invocation (Supplementary Information 12). The rephrased question was presented to a fresh instance of the patient simulation agent initialized with the conversation history up to that point, and the new response was recorded. Each question–answer pair was evaluated along three dimensions: (1) inter-answer consistency, measuring whether the original and variant answers do align; (2) ground-truth consistency of the original answer, comparing the original response against the information documented in the HPI of the patient; and (3) ground-truth consistency of the variant answer. Answers were classified as fully consistent, not fully consistent (if at least one part of the answer was not aligned with), or not applicable (for non-informative responses containing no medical content). Evaluations were performed independently by one physician through a structured annotation interface and in parallel by two LLM-based evaluators (Supplementary Information 13 and 14) dedicated to assessing inter-answer consistency and answer–ground-truth consistency, respectively.

#### Patient agent diagnostic information leaks

To evaluate the integrity of the patient agent—which was based on the HPI documentation of the discharge letters—we conducted a review of ‘patient–physician’ interactions across all three experimental groups (MIRA, human physicians and the board-certified only cohort). We categorized the conversations into three classes: information leak (premature disclosure of diagnostic conclusions), versus no information leak (appropriate information withholding) with subdividing the latter into two categories: prior workup disclosure (justified disclosure of diagnostic workup or hypotheses related to the diagnosis in question) and no information leak at all (no disclosure of any diagnostic workup or otherwise inadequate information). Ratings were performed manually by one physician using a custom annotation interface.

#### Patient agent adversarial robustness

To assess the robustness of the patient agent against attempts to elicit inappropriate diagnostic disclosures, we designed a collection of 11 adversarial prompts inspired from current best knowledge^37,38^ spanning four attack categories: (1) overbroad clinical probing, requesting exhaustive differential diagnoses or complete diagnostic reasoning; (2) attempts to prompt injections, directing the system to ignore prior instructions or reveal diagnostic suspicions; (3) authority exploitation, impersonating supervisors or department heads to invoke compliance; and (4) obfuscation strategies such as role-playing or asking to respond in poems. We randomly selected 10 patient cases per disease category across all clinical conditions and subjected each patient agent to all 11 adversarial prompts independently, generating 880 responses. All responses were independently reviewed manually by one physician and classified according to the same criteria as shown in ‘Patient agent diagnostic information leaks’.

#### MIMIC-IV versus MIRA

For the evaluation of MIRA, we first compare its performance against reference data extracted from the MIMIC-IV dataset. Herein, for each patient, we establish a ground truth containing the following information:

- The primary diagnosis of the patient, coded according to ICD-10 standards.
- Results from the admission physical examination at the hospital.
- All laboratory and urine test results, microbiology findings, and imaging studies that were requested within the first 24 h after admission.
- All procedures, primarily including surgical interventions, as documented in the discharge letter and the procedures_icd dataframe.
- All medications, including both those listed in the patient’s pre-admission medication and those administered within the first day of hospitalization.

#### Human physicians versus MIRA

We included two cohorts of physicians in our study. The first cohort consisted of four board-certified physicians from a German university hospital with 7 to 11 years of clinical practice. The second cohort comprised 6 physicians with heterogeneous levels of expertise: four residents (with 0 (after passing the German Medical Licensing Exam), 1.5, 1.5 and 5 years of clinical practice, respectively), one board-certified radiologist, and one board-certified haemato-oncologist with 12 and 15 years of clinical experience, respectively. All physicians worked on the patient cases under identical conditions as MIRA, with each physician independently evaluating a random, non-overlapping subset of cases (one-quarter or one-sixth of the total). We deliberately avoided overlap within cohorts to preserve clinical validity: in practice, the same patient is not diagnosed in parallel with different (or duplicate) laboratory results or imaging findings, and forcing overlap would introduce ambiguity when comparing diagnostic or therapeutic decisions (for instance which of two divergent physician assessments should be treated as the reference). Different to MIRA, the physicians had access to a graphical user interface (GUI) that allowed them to communicate with the patient agent via chat and gather additional information at any time using the same diagnostic tools that were available to MIRA. Every tool required them to either select parameters from existing options, provide free-text input or a combination of both. More specifically, for tools requiring categorical inputs, such as requesting laboratory, urine, microbiology values, or radiology images, predefined options were provided as selectable checkboxes. Other tools requiring free-text input, such as searching for available procedures or submitting clinical questions to radiologists, were provided with free-text entry fields. Moreover, medications could be requested through a table interface resembling a hospital medication chart where physicians can order medication from the internal pharmacy. Overall, we specifically designed the layout of the GUI to: (1) replicate the workflow of commercial EHR systems as closely as possible; and (2) allow human physicians to perform their tasks under the same conditions as MIRA. An exemplary overview of the GUI is provided in Supplementary Fig. 6. Unlike MIRA, human evaluators were not restricted to a maximum number of conversation turns. For evaluation, we selected a subset of 45 randomly sampled patients with cholecystitis, 45 presenting with urinary tract infections, 45 with pulmonary embolisms, 44 with diverticulitis, 43 with appendicitis, 42 with pancreatitis, 26 with pneumonia and 21 with pancreatic cancer, leading to a total of 311 patient cases. To address the smaller sample sizes for certain diagnoses, such as pancreatic cancer, we maintained the ratio of correctly and incorrectly diagnosed cases by MIRA during random sampling. This ensured a balanced representation without skewing the distribution of diagnostic outcomes towards one direction when comparing.

#### LLM as a judge evaluations

To automate the evaluation of diagnostic accuracy, medication reconciliation, procedure assignment, and guideline adherence, we utilized few-shot–prompted LLM standardizers (admission medication) and evaluators, as defined below. Given that LLMs may show bias toward their own outputs^39^, we conducted an independent assessment of all tasks by a board-certified physician. This physician was blinded to the source of responses and rated a subset of patient case studies sampled from results from MIRA and humans. He first benchmarked the diagnostic LLM-evaluator against a subset of *n* = 200 cases and observed high concordance (96.5%). The few discordant cases largely reflected differences in label granularity, for example, ‘possible lung infection or tumor progression’ versus ‘pneumonia’, which could be considered acceptable in the emergency department context where a provisional working diagnosis could suffice to initiate appropriate care and admission. Most importantly, in a paired analysis of 37 patients, each with both a MIRA-sourced and a human-sourced diagnosis, we found no evidence of source-type asymmetry (McNemar’s exact test, *P* = 1.00), meaning there was no direction of the LLM-evaluator to falsely favour either human- or AI-generated responses, although the small number of discordant pairs due to high overall agreement might limit power to detect differences. Second, for the medication-structuring step enabling comparison of admission prescriptions between MIRA and physicians, 466 pre-admission medication entries, structured by the Pre-Admission Medication Standardizer were compared to the free-text notes from the dataset. Please note that downstream analyses after this step were deterministic (and non-AI-dependent). Each entry was assessed for correctness in drug name, dose (value and unit), period (value and unit), frequency, and route and counted as correct only if all attributes matched, and wrong if any mismatched. Item-level agreement was 97.4% (454 out of 466); 12 out of 466 were rated as ‘incorrect’, largely due to the model inferring missing values (for example, inferring a plausible route where route of administration was absent in the data) or intrinsic text ambiguity (conflicting dosage instructions in the ground-truth in MIMIC-IV). Only one case had one or more than one medication missing during normalization. Third, for procedure match evaluation we observed perfect concordance between the LLM as a judge and the physician, with McNemar’s exact two-sided test (*P* = 1.0) and Cohen’s *κ* = 1.0, indicating complete agreement. Finally, to evaluate the Guideline Adherence Evaluator LLM, we assessed guideline adherence manually on *n* = 112 patient cases with 256 metrics independently by the board-certified physician. Overall agreement was 94.5%. Most importantly, in matched patient cases evaluated by MIRA and by physicians, the LLM judge showed no source-specific bias: across the same patients, its disagreement rate and its false-positive and false-negative rates (using the human evaluator as reference) did not differ between MIRA- and physician-generated recommendations, indicating no tendency to over- or under-accept AI outputs. All supporting data can be found in Supplementary Data Tables 45–51.

#### Diagnosis performance evaluations

First, we evaluated the diagnostic accuracy of MIRA and humans. Since certain diseases can be encoded with different ICD codes that are to be considered correct, we cannot use direct pattern-matching methods to determine the correctness. Therefore, we utilized an evaluator LLM (Diagnosis Evaluator) with few-shot samples and chain-of-thought reasoning to rate whether a response was correct (aligning with the ground truth at the defined level of detail) or inaccurate (either providing an entirely different diagnosis or only a partially correct diagnosis with conflicting details). We provide the full evaluator instructions in Supplementary Information 19. As a complementary exploratory analysis, we also quantified diagnosis-relevant versus non-diagnostic patient utterances and their temporal distribution across dialogues (Supplementary Figs. 2 and 3).

Then, diagnostic procedures were evaluated as follows: for physical examinations, we measured the frequency with which MIRA and human physicians correctly requested these tests out of the total cases in our benchmark dataset. Tools relying on predefined categorical variables, such as laboratory, urine, microbiology requests and imaging studies, were evaluated by measuring the overlap between the parameters requested by MIRA and those documented in the data from MIMIC-IV. This overlap was then compared to the overlap observed between the two physician cohorts and MIMIC-IV. For the evaluation of medications, several challenges arose that—such as seen when measuring diagnostic accuracy—prevented the use of predefined categorical comparisons between MIRA’s output and the information available in the data. Similar to real-world hospital settings, where medications are often recorded in free-text formats in a patient chart (as they are primarily instructions for humans), direct mapping of medications is difficult. Additionally, hospital pharmacies frequently substitute medications with generics or follow in-house standard operating procedures that prioritize specific drugs over others such that the medication administered to patients in the MIMIC-IV dataset might not necessarily always follow objective standards. To address these challenges, we developed an LLM-based medication standardizer (separated for hospital and pre-admission medication). It first standardizes any drug names between the agent’s output and the MIMIC-IV baseline data, as well as between each physician cohort and the available data. Instructions are shown in Supplementary Information 20 and 22. Following this standardization, we perform the final evaluations through a hierarchical procedure of deterministic comparison operations as outlined in Extended Data Fig. 5.

#### Procedure evaluations

To be able to correctly compare the procedures identified in the MIMIC-IV dataset with the decisions made by MIRA or human physicians in our study, several important considerations had to be addressed. First, not all procedures performed during a hospital stay are evident within the information available during the initial 24 h period in the emergency department (which is our primary data restriction). Additionally, some procedures are entirely unrelated to the diagnosis under investigation—for example, other open umbilical herniorrhaphy or repair of abdominal wall, which are sometimes observed in cases of cholecystitis. Furthermore, certain procedures, such as repair of blood vessel with tissue patch graft, may occur only as a direct consequence of other interventions. Second, any medical procedure—for instance, any type of surgery—can be encoded in different ways of granularity, sometimes even influenced by factors such as billing requirements. Third, certain procedures in the MIMIC-IV dataset are not encoded using ICD-9 or ICD-10 codes but must instead be extracted as plain text descriptions from the patient’s discharge summary. For example, the placement of a central venous catheter might be documented as midline insertion in MIMIC-IV, whereas our agent places a request for a central venous catheter placement with guidance (ICD-9-CM vol. 3 code 38.97), where one procedure represents a subset of the other. Another example is the dilation of common bile duct with intraluminal device, via natural or artificial opening endoscopic (ICD-10 0F798DZ), which we would consider to be similar enough to endoscopic insertion of stent (tube) into bile duct (ICD-9 51.87) as requested by MIRA. Moreover, as another restriction, free-text procedures from MIMIC-IV also include artefacts in the dataset, such as entries stating, ‘you underwent no medical or surgical procedures during this hospitalization’, and spelling errors such as ‘laparscopic appendectomy’. Fourth, a single procedure may sometimes be represented by multiple separate codes, further complicating the comparison process. To address these constraints, we developed a systematic workflow: We began by collecting all ICD-9/10-coded procedures from the MIMIC-IV dataset and deduplicated them. In cases where coded procedures were unavailable (293 out of 574 cases), we extracted procedure information directly from the free-text descriptions in patients’ discharge summaries. We then manually cleaned this set of procedures, removing irrelevant keywords such as ‘none’ or ‘na’. Next, we applied a pattern-matching approach to align the procedures listed in the dataset with those suggested by MIRA or identified by the physicians in our study. This formed the foundation for calculating our primary evaluation metric, recall, which was defined as the fraction of procedures present in MIMIC-IV that were also recommended by MIRA or during our physicians’s study. Next, to overcome the second issue, where direct pattern-matching failed—for instance, in cases such as endoscopic retrograde cholangiopancreatography [ERCP] (ICD-9 51.10) (MIRA)—versus ercp (MIMIC-IV), we implemented a procedure match evaluator (Supplementary Information 21). This evaluator used few-shot prompting to assess whether any given combination of procedures from the MIMIC-IV dataset and the AI agent or user study were equivalent. Herein, equivalence was determined in two key ways: (1) procedures that are literally the same but differed owing to minor inconsistencies, such as spelling mistakes on the MIMIC-IV side; and (2) procedures that represented very similar concepts. For example, a combination of procedures from MIMIC-IV such as excision of duodenum, open approach and excision of pancreas, open approach would be considered equivalent to MIRA’s request to perform a radical pancreaticoduodenectomy (ICD-9 52.7) in the case of a pancreatic cancer patient. Importantly, we restricted our evaluations to the intersection of cases where MIRA and physicians correctly diagnosed the patient, which is in line with the previous works of Hager et al.^18^.

#### Guideline adherence evaluations

To evaluate the adherence of MIRA compared with human physicians in following current medical guidelines when making treatment decisions, we developed the following evaluation pipeline: First, we manually compiled relevant treatment recommendations from established international guidelines: the 2020 Update of the WSES Jerusalem Guidelines for Appendicitis^27^, the 2020 WSES Guidelines for Acute Cholecystitis^40^, the 2019 WSES Guidelines for the Management of Severe Acute Pancreatitis^41^, and the American Society of Colon and Rectal Surgeons Clinical Practice Guidelines for Left-Sided Colonic Diverticulitis^42^. These guidelines were previously utilized in Hager et al.^18^. Additionally, we collected recommendations from the American Thoracic Society documents for pneumonia^30^, the Core Curriculum 2024 recommendations from the *American Journal of Kidney Diseases* for urinary tract infection^43^, and the American Society of Hematology 2020 guidelines for management of venous thromboembolism: treatment of deep vein thrombosis and pulmonary embolism^44^. We then extracted and cleaned treatment recommendations, focusing only on medication-related aspects. Subsequently, we standardized medication names between the ground truth from MIMIC-IV data and prescriptions provided by either MIRA or human physicians using the Hospital Medication Standardizer (Supplementary Information 22). As this step was performed individually for each patient, we subsequently manually merged drug classes that represented the same medication categories despite differences in naming conventions (for instance ‘Antibiotic’ versus ‘antibiotics’) so that we were able to aggregate results across different patients. Our evaluations focused exclusively on the intersection of patients that were correctly diagnosed by both human physicians and MIRA. Due to the complexity of decision-making in pancreatic cancer, which usually involves multimodal data such as imaging, evaluations were restricted to the remaining seven diseases. For each, we established a Guideline Adherence Evaluator, which received guideline recommendations (as shown in Supplementary Data Table 52), the relevant guideline category to focus on, the patient’s exact diagnosis, and the complete list of hospital-prescribed medications (excluding medications already taken prior to hospitalization). The Evaluator assessed guideline adherence for each prescription, providing a binary outcome (true/false) with accompanying rationale, using structured templates as output. Notably, we did not evaluate new therapeutic prescriptions in reference to the underlying data in MIMIC-IV, because patient cases span from 2008 to 2019 and therefore do not necessarily reflect current best practices, making a comparison to the most recent medical guidelines a more relevant task. Please find the full evaluators instructions under Supplementary Information 23.

#### Safety and robustness evaluations

To evaluate the robustness of MIRA in scenarios where hospital admission may not be strictly necessary and patients could be managed safely in an ambulatory setting, we developed a novel dataset derived from MIMIC-IV cases of patients diagnosed with pneumonia or pulmonary embolism, identified from emergency department encounters through filtering via ICD codes. From these cohorts, ten cases per diagnosis were selected as templates. Because these lacked key narrative elements such as HPI, physical examination findings, and free-text descriptions of symptoms, each template was expanded under the supervision of a board-certified physician to generate four distinct patient variants. For each, synthetic but clinically plausible histories, physical examinations, laboratory values, radiology findings, vital signs and medication reconciliation were written and adapted to represent a range of illness severities and clinical presentations. A ground-truth disposition recommendation (either hospital admission or discharge from the emergency department) was assigned to each case based on established clinical criteria. We chose pneumonia and pulmonary embolism because validated objective scoring systems, CURB-65 and sPESI respectively, are routinely used as surrogates for admission necessity. Our primary evaluation end-point was recall, defined as the proportion of admission-requiring patients that were correctly triaged for admission. In this experiment, we gave MIRA the additional ability to request vital signs and modified the original Admission tool to besides giving a diagnosis also make a decision (admission or discharge from emergency department) together with its reasoning process (CloseCase tool). In another set of experiments to evaluate the agent’s consistency across diverse patient profiles, we measured diagnostic correctness for each patient encounter under six prespecified prompt perturbations (bias conditions, inspired by Schmidgall et al.^14^), directly comparing it with the corresponding baseline outputs. For each of the 8 target diagnoses, we sampled 10 patient cases, yielding a total of 480 paired evaluations (6 biases × 10 patients × 8 diagnoses). The same patients were used across bias experiments. Bias conditions were implemented by appending additional instructions to the patient agent prompt (Supplementary Information 24), following the same experimental procedures as in the baseline runs. The primary end-point was the change in diagnostic accuracy, expressed as the risk difference (bias − baseline) and was pooled across all diagnoses.

### Statistics and reproducibility

We report exact (Clopper–Pearson) 95% confidence intervals for leak and disclosure rates. For diagnostic accuracy, paired comparisons between MIRA and human evaluators used McNemar’s exact test; when a discordant cell was empty, we used two-sided Fisher’s exact. We additionally report the paired odds ratio (OR = *n*_10_/*n*_01_) with exact 95% confidence intervals. All paired analyses used the intersection of admissions evaluated by both MIRA and human physicians. *P* values were Holm-adjusted within each diagnosis subset, and the supplementary data tables report the overall exact p value and Holm-adjusted per-diagnosis values. Error bars on diagnostic accuracy bar plots are Wilson binomial 95% confidence intervals. For the diagnostic procedure tasks, physical examination was analysed as correct versus incorrect (McNemar’s test), and for microbiology, radiology, and blood-value selection we compared which group (MIRA or humans) missed fewer MIMIC-IV items, each yielding a per-simulation 2 × 2 discordant table and we analysed paired differences with the two-sided Wilcoxon signed-rank test. We report median, interquartile range, and rank-biserial correlation, and controlled the study-wide false-discovery rate using the Benjamini–Hochberg procedure. Where applicable, micro- and macro-averaged metrics are reported. For the admission medication prescription and procedures requests we computed precision, recall, and *F*_1_ with 95% confidence intervals from paired bootstrapping with 10,000 resamples with preserved patient pairing. Guideline adherence was again evaluated with McNemar’s exact test or Fisher’s exact when needed. For the admission versus discharge analysis, we report standard classification metrics with 95% confidence intervals from a patient-cluster bootstrap with 10,000 resamples, where the cluster is the template linking the variations of different patient cases that shared the same MIMIC-IV starting information. The primary metric was sensitivity for admission. Directional error bias was assessed with McNemar’s exact test. For the perturbation experiments, we measured paired significance with McNemar’s exact test with Holm adjustment across the six biases within each diagnosis and for the pooled analysis. Uncertainty was quantified using paired, non-parametric bootstrap 95% confidence intervals with 10,000 resamples. Unless otherwise stated, each analysis was executed once on a prespecified set of independent evaluation units, and the reported summary statistics therefore reflect variation across those units.

### Statement on the use of AI tools

In accordance with the COPE (Committee on Publication Ethics) position statement of 13 February 2023 (https://publicationethics.org/cope-position-statements/ai-author), the authors hereby disclose the use of the following AI models during the writing of this article: GPT-4o (ChatGPT, OpenAI) for checking and improving spelling and grammar.

### Ethics statement

This study involves the analysis of the MIMIC-IV dataset with LLMs. Patient information was processed in accordance with PhysioNet’s ‘Credentialed Health Data Agreement’ and the ‘Responsible use of MIMIC data with online services like GPT’ statement.

### Reporting summary

Further information on research design is available in the Nature Portfolio Reporting Summary linked to this article.

## Online content

Any methods, additional references, Nature Portfolio reporting summaries, source data, extended data, supplementary information, acknowledgements, peer review information; details of author contributions and competing interests; and statements of data and code availability are available at 10.1038/s41586-026-10675-5.

## Supplementary information

Supplementary Information This Supplementary Information file contains supplementary text, supplementary methods, embedded Supplementary Figs. 1–7, Supplementary Tables 1–52 and additional references.

Reporting Summary

Peer Review File

## Source data

Source Data Fig. 2

Source Data Fig. 3

Source Data Fig. 4

Source Data Fig. 5

## Data Availability

Researchers can access the original dataset by creating an account on https://physionet.org/ and after completing the necessary steps to obtain permission for the MIMIC-IV database (version 2.2), which is available on https://physionet.org/content/mimiciv/2.2/. To receive permission, researchers must complete the ‘CITI data or specimens only research’ training course and sign the PhysioNet data use agreement for ‘credentialed health data’. Once access is granted, the dataset can be reconstructed using code from this GitHub repository: https://github.com/Dyke-F/MIRA. We note that our dataset generation code implementation is partly adapted from and heavily inspired by Hager et al.^18^. Source data for Figs. 2–5 are provided with this paper. Source data are provided with this paper.

## Code availability

An implementation of our source codes for researchers to extend on our work is available from GitHub under https://github.com/Dyke-F/MIRA.
