# Supplementary material for: Towards autonomous medical artificial intelligence agents
Source: Nature. 2026 Jun 17;655(8125):1282–91. doi: 10.1038/s41586-026-10675-5 (PMC13421332; doi:10.1038/s41586-026-10675-5)
Supplement: Supplementary file 2 — Reporting Summary [file 41586_2026_10675_MOESM2_ESM.pdf]

nature portfolio

Corresponding author(s): Dyke Ferber and Jakob Nikolas Kather

Last updated by author(s): April 8, 2026

Reporting Summary

Nature Portfolio wishes to improve the reproducibility of the work that we publish. This form provides structure for consistency and transparency in reporting. For further information on Nature Portfolio policies, see our Editorial Policies and the Editorial Policy Checklist.

Statistics

For all statistical analyses, confirm that the following items are present in the figure legend, table legend, main text, or Methods section.

n/a	Confirmed	
☐	☒	The exact sample size (*n*) for each experimental group/condition, given as a discrete number and unit of measurement
☐	☒	A statement on whether measurements were taken from distinct samples or whether the same sample was measured repeatedly
☐	☒	The statistical test(s) used AND whether they are one- or two-sided<br>*Only common tests should be described solely by name; describe more complex techniques in the Methods section.*
☐	☒	A description of all covariates tested
☐	☒	A description of any assumptions or corrections, such as tests of normality and adjustment for multiple comparisons
☐	☒	A full description of the statistical parameters including central tendency (e.g. means) or other basic estimates (e.g. regression coefficient) AND variation (e.g. standard deviation) or associated estimates of uncertainty (e.g. confidence intervals)
☐	☒	For null hypothesis testing, the test statistic (e.g. *F*, *t*, *r*) with confidence intervals, effect sizes, degrees of freedom and *P* value noted<br>*Give P values as exact values whenever suitable.*
☒	☐	For Bayesian analysis, information on the choice of priors and Markov chain Monte Carlo settings
☒	☐	For hierarchical and complex designs, identification of the appropriate level for tests and full reporting of outcomes
☒	☐	Estimates of effect sizes (e.g. Cohen's *d*, Pearson's *r*), indicating how they were calculated

Our web collection on statistics for biologists contains articles on many of the points above.

Software and code

Policy information about availability of computer code

Data collection	During data collection, no code was involved as the data was manually downloaded from physionet.org
Data analysis	Repository: https://github.com/Dyke-F/MIRA<br>License: MIT<br>Core study code: src/ (dataset preparation, backend/FHIR integration, simulation runs, evaluation scripts)<br>Language/environment: Python 3.12<br>Core analysis software: pandas==2.2.2, numpy==2.1.1, statsmodels>=0.14.4, matplotlib==3.9.2, seaborn==0.13.2<br>Core runtime/software components: openai==1.44.1, fhir-resources==7.1.0, pydantic>=2.11.4, qdrant-client==1.12.0<br>Full pinned dependency list: src/pyproject.toml and src/uv.lock

For manuscripts utilizing custom algorithms or software that are central to the research but not yet described in published literature, software must be made available to editors and reviewers. We strongly encourage code deposition in a community repository (e.g. GitHub). See the Nature Portfolio guidelines for submitting code & software for further information.

Data

Policy information about availability of data

All manuscripts must include a data availability statement. This statement should provide the following information, where applicable:

- Accession codes, unique identifiers, or web links for publicly available datasets
- A description of any restrictions on data availability
- For clinical datasets or third party data, please ensure that the statement adheres to our policy

Researchers can access the original dataset by creating an account on https://physionet.org/ and after completing the necessary steps to obtain permission for the MIMIC-IV database (version 2.2), which is available on https://physionet.org/content/mimiciv/2.2/. To receive permission, researchers must complete the "CITI data or specimens only research" training course and sign the PhysioNet data use agreement for "credentialed health data." Once access is granted, the dataset can be reconstructed using code from this GitHub repository: https://github.com/Dyke-F/MIRA. Source data for Figures 2-5 are provided with this paper.

Research involving human participants, their data, or biological material

Policy information about studies with human participants or human data. See also policy information about sex, gender (identity/presentation), and sexual orientation and race, ethnicity and racism.

Reporting on sex and gender	Data was de-identified in the MIMIC-IV dataset and only "administrative gender" was reported. Therefore no further gender based analysis was performed in this study. For the perturbation experiments and the admission/discharge from ED experiments, we have synthetically modified patient cases by flipping the stored information in MIMIC-IV or adding novel synthetic information.
Reporting on race, ethnicity, or other socially relevant groupings	As data was de-identified by the authors of the MIMIC-IV dataset, no analysis on race, ethnicity or other socially relevant groupings was performed in this study.
Population characteristics	Given the de-identification of the data, no population-based characteristics were analysed.
Recruitment	There was no recruitment of participants in this study.
Ethics oversight	Data was obtained from physionet.org: Following the MIMIC-IV Website: https://physionet.org/content/mimic-iv-note/2.1/:<br><br>Ethics<br>The collection of patient information and creation of the research resource was reviewed by the Institutional Review Board at the Beth Israel Deaconess Medical Center, who granted a waiver of informed consent and approved the data sharing initiative.

Note that full information on the approval of the study protocol must also be provided in the manuscript.

Field-specific reporting

Please select the one below that is the best fit for your research. If you are not sure, read the appropriate sections before making your selection.

☒ Life sciences ☐ Behavioural & social sciences ☐ Ecological, evolutionary & environmental sciences

For a reference copy of the document with all sections, see nature.com/documents/nr-reporting-summary-flat.pdf

Life sciences study design

All studies must disclose on these points even when the disclosure is negative.

Sample size	The final sample size contained 574 independent patients. No formal a priori sample-size calculation was performed because this was a retrospective study based on a fixed source population. Instead, we included all available patients who met prespecified eligibility, diagnostic, and data-completeness criteria; these criteria were defined before experimental data use.
Data exclusions	Data exclusions (all criteria were defined before data extraction and applied by two board-certified clinicians):<br><br>Diagnosis mismatch or late diagnosis – Hospital stays in which the target ICD-9/10 code was not the principal discharge diagnosis and the same diagnosis was absent from the emergency-department admission note were discarded to avoid cases where the correct diagnosis emerged only later in the stay.<br><br>Incomplete or missing imaging – Records were excluded when required imaging was lacking or could not be parsed (e.g., no chest study for pneumonia; no abdominal study for appendicitis; modality or anatomical region not identifiable; transfers relying solely on outside imaging).<br><br>Insufficient first-24-h data for the agent – Cases without laboratory or microbiology results within the first 24 h, or without a documented history of present illness, physical-examination findings, or blood tests, were removed.

Other critical gaps – Encounters with incomplete radiology metadata, absent procedure information, or redacted content that eliminated essential clinical context were excluded.

After applying these a-priori rules, 574 fully evaluable patient stays from MIMIC-IV constituted the final benchmark used in all subsequent analyses.

Replication	Results can be replicated with the code that will be made publically available.
Randomization	No patient allocation to experimental groups was performed because this was a retrospective benchmark evaluation of a fixed cohort and did not involve model training or treatment assignment. Formal covariate adjustment was therefore not applicable for MIRA-only analyses. In head-to-head comparisons, covariates were controlled by design because MIRA and physicians evaluated the same patient cases under identical information conditions, and analyses were restricted to paired cases assessed by both groups.
Blinding	There was no blinding as there was no different groups. Humans and MIRA were blinded in a sense, that they could only use chat and tools to obtain further information on the patients.

Reporting for specific materials, systems and methods

We require information from authors about some types of materials, experimental systems and methods used in many studies. Here, indicate whether each material, system or method listed is relevant to your study. If you are not sure if a list item applies to your research, read the appropriate section before selecting a response.

Materials & experimental systems

n/a	Involved in the study
☒	☐ Antibodies
☒	☐ Eukaryotic cell lines
☒	☐ Palaeontology and archaeology
☒	☐ Animals and other organisms
☒	☐ Clinical data
☒	☐ Dual use research of concern
☒	☐ Plants

Methods

n/a	Involved in the study
☒	☐ ChIP-seq
☒	☐ Flow cytometry
☒	☐ MRI-based neuroimaging

Plants

Seed stocks	*Report on the source of all seed stocks or other plant material used. If applicable, state the seed stock centre and catalogue number. If plant specimens were collected from the field, describe the collection location, date and sampling procedures.*
Novel plant genotypes	*Describe the methods by which all novel plant genotypes were produced. This includes those generated by transgenic approaches, gene editing, chemical/radiation-based mutagenesis and hybridization. For transgenic lines, describe the transformation method, the number of independent lines analyzed and the generation upon which experiments were performed. For gene-edited lines, describe the editor used, the endogenous sequence targeted for editing, the targeting guide RNA sequence (if applicable) and how the editor was applied.*
Authentication	*Describe any authentication procedures for each seed stock used or novel genotype generated. Describe any experiments used to assess the effect of a mutation and, where applicable, how potential secondary effects (e.g. second site T-DNA insertions, mosiacism, off-target gene editing) were examined.*
