# Supplementary material for: Towards autonomous medical artificial intelligence agents
Source: Nature. 2026 Jun 17;655(8125):1282–91. doi: 10.1038/s41586-026-10675-5 (PMC13421332; doi:10.1038/s41586-026-10675-5)
Supplement: Supplementary file 3 — Peer Review File [file 41586_2026_10675_MOESM3_ESM.pdf]

nature portfolio

Peer Review File

Towards Autonomous Medical Artificial Intelligence Agents

Corresponding Author: Professor Jakob Kather

Any redactions in this file are there to maintain patient confidentiality, the confidentiality of unpublished data, or to remove third-party material.

This file contains all reviewer reports in order by version, followed by all author rebuttals in order by version.

Version 0:

Reviewer comments:

Referee #1

(Remarks to the Author)
This study presents AIDOC, an ambitious AI agent designed to operate within an EHR-like environment, performing a full spectrum of clinical tasks from patient dialogue to diagnosis and treatment planning. The work is a notable step forward in the field of medical AI, with a commendable focus on building within an interoperable FHIR framework and evaluating a complete, end-to-end clinical workflow.

However, while the paper's aspirations are high, its central claims of "fully autonomous" operation and superior performance over human physicians are not yet supported by the evidence presented. The study is limited by a lack of transparency, an opaque and potentially biased evaluation pipeline, performance metrics that systematically favor the AI, an unrepresentative human comparator group, and an idealized evaluation environment.

Strengths of the Study

The authors should be recognized for several significant contributions that advance the field:
FHIR-Based Architecture: The decision to build the agent within a framework compliant with Fast Healthcare Interoperability Resources (FHIR) is a major strength. It demonstrates a clear and practical path toward real-world integration with existing Electronic Health Record (EHR) systems.

End-to-End Agentic Workflow: The study evaluates a multi-step, agentic process that mimics a clinical workflow—from taking a history and ordering diagnostics to planning therapeutics and admission. This holistic approach is a significant advance over prior models that perform only narrow, isolated tasks.

Critical Flaws and Actionable Suggestions

Despite its strengths, the study suffers from several severe flaws that undermine the validity and generalizability of its conclusions. These are organized below, with specific, fixable suggestions for the authors.

1. Lack of Transparency and Verifiability During Review
The Flaw: The most significant barrier to the credibility of this work is that its core components were unavailable for review. The authors state they "plan to release" the code, but at the time of review, the AIDOC agent, the patient simulator, and the evaluation framework remain a "black box."
The Actionable Suggestion: For the results to be considered verifiable, the authors must provide access to the full source code, evaluation prompts, and data processing scripts.

2. The Evaluation Pipeline is Opaque and Potentially Biased
The Flaw: The study's headline metrics are scored by multiple (≥5) proprietary GPT-4o-based "Evaluators." Since the AIDOC agent itself is powered by the same model family, this creates a significant risk of "style-matching bias," where an LLM may rate outputs more favorably if they mirror its own phrasing and reasoning style. Only the Diagnosis Evaluator is reported to have undergone a human audit; no parallel check is described for the medication, procedure, or guideline judges.

The Actionable Suggestion: To ensure fairness and transparency, the authors should:
Re-score a random 10-20% of cases for the Medication, Procedure, and Guideline-Adherence tasks with board-certified clinicians to validate the LLM judges' accuracy.
Alternatively, use a model from a different vendor (e.g., Anthropic Claude) as a neutral adjudicator to show that the conclusions hold.

3. Performance Metrics Systematically Favor the AI's Verbosity over Clinical Competence
The Flaw: The metrics for medication and procedure matching are designed in a way that creates an illusion of superior AI performance.
Medications (PMMR): The metric only rewards explicit, line-item prescriptions. AIDOC is prompted to list every drug, while human physicians can use the clinically appropriate shorthand "Continue home meds." As the authors explicitly note, this is incorrectly scored as zero matches. The resulting 94.6% vs. 5.1% gap therefore measures compliance with a system prompt, not clinical competence.
Procedures (PMR): For procedures, there is no penalty for over-ordering, unlike for diagnostic tests where a Tversky distance metric was used. The AI is given credit for both exact and "equivalent" codes identified by its sibling LLM evaluator, which inflates its recall score by counting near-duplicates without assessing precision.
Crucially, none of the headline metrics assess safety elements such as drug-drug interactions, renal dosing adjustments, or cumulative radiation exposure.
The Actionable Suggestion: To create a fair comparison, the authors must revise their metrics.
Medications: Treat "continue home meds" as a full match or score at the drug-class level. Report precision to penalize unnecessary medication changes.
Procedures: Report both precision and recall, introducing a penalty for superfluous requests.

4. The Human Physician Comparator Group is Not Representative and Untapped for Crucial Feedback
The Flaw: The human comparator cohort—composed of four residents and two subspecialists—is not representative of the expert clinicians who typically manage these acute conditions. Critically, none were board-certified emergency medicine physicians. The finding that AIDOC outperformed this mixed-experience group does not sufficiently support the broader claim of outperforming "human physicians" in this domain. Furthermore, the study misses a key opportunity to gather feedback from clinicians on the simulation's fidelity.
The Actionable Suggestion: A more rigorous evaluation would involve two steps:
Repeat the human evaluation with a cohort of board-certified emergency medicine physicians to provide an appropriate performance benchmark.
Following the evaluation, survey these physicians to assess the simulation's realism. This should include quantitative ratings (e.g., a 1-10 scale on the realism of the patient dialogue, tool availability, and information flow) and qualitative feedback on the most significant differences between the simulation and actual clinical practice. This would provide crucial context for interpreting the performance data.

5. The Evaluation Environment Lacks Clinical Realism
The Flaw: The study's framework, while applied symmetrically, lacks key elements of clinical reality. When a test is requested that was not performed in the original case, the system returns "N/A." Furthermore, the simulation operates in a static, sanitized environment that ignores temporal dynamics, resource constraints, and data ambiguity. Finally, all encounters map to one of eight single-diagnosis templates, omitting the multimorbid or nonspecific presentations common in emergency medicine.
The Actionable Suggestion: To test the agent's resilience, the authors should conduct a "Wizard of Oz" experiment. A human clinician would "play" the role of the sandboxed EHR, intentionally introducing realistic delays, resource constraints, and actual results for counterfactual requests to assess AIDOC's adaptive reasoning.

6. Dataset Limitations: Filtering and Ground Truth Definition Threaten Generalizability
The Flaw: The study's conclusions are built on a narrow and potentially biased data foundation. First, the process of manually curating 574 encounters from the larger MIMIC-IV corpus (exact denominator not specified) raises concerns about selection bias. Second, the study relies solely on the final MIMIC-IV discharge diagnosis as its ground truth. This is a potential limitation, as this diagnosis may be informed by data unavailable during the initial ED encounter, making it an imperfect benchmark for real-time clinical judgment.
The Actionable Suggestion: To address these data limitations, the authors should:
Provide a detailed data selection flowchart (e.g., a CONSORT diagram) and test AIDOC on a sample of excluded cases to evaluate its failure modes.
Validate the ground truth itself by performing a sensitivity analysis: have a panel of independent emergency physicians establish a consensus diagnosis for a subset of cases based only on the initial presentation data. Comparing AIDOC's accuracy against both the MIMIC-IV diagnosis and this expert consensus would demonstrate the robustness of its performance.

7. The Statistical Approach Could Be Strengthened
The Flaw: For comparing diagnostic test selection, the authors correctly use McNemar's test on a binary outcome. While statistically valid, this approach reduces a richer "count" of missed tests to a simple win/loss/tie, resulting in a loss of information about the magnitude of the difference. Additionally, given the multiple outcomes assessed across the study, the risk of a false-positive finding due to multiplicity is not discussed.
The Actionable Suggestion: To demonstrate robustness, the authors should perform a sensitivity analysis using a non-parametric test on the paired count differences, such as the Wilcoxon signed-rank test. A discussion of potential family-wise error or a sensitivity analysis using a correction (e.g., FDR) would also increase confidence in the reported p-values.

(Remarks on code availability)
Code was not provided.

Referee #2

(Remarks to the Author)
Summary:
The work introduces AIDOC, an autonomous medical AI agent designed to operate within EHRs. The system performs a wide range of medical tasks from within the sandbox context of a virtual EHR environment - from taking patient medical histories, to ordering tests, to interpreting them and prescribing medications among others. The authors evaluate against retrospective patient cases from MIMIC-IV and AIDOC achieves superior diagnostic accuracy compared to physicians. The authors stress on the performance of medication prescriptions and adherence to medical guidelines.

Clarity and context:
The paper is generally well-written and easy to follow.

Originality and significance:
The main strengths of the paper are technical and simulation of the end-to-end clinical workflow integration. The interesting parts are the agent setup with tools and mimicking a realistic clinical setup requiring navigating of the many options available to practicing physicians.This could be a potential important benchmark / environment towards building AI agents with real world clinical applicability. There is no code available at this time to inspect and review this part.

At the same time, the actual clinical performance of AIDOC appears to be somewhat unclear and lacking compared to deep, nuanced evaluations of AI healthcare agents like OpenAI healthbench and Google AMIE. There are several potential overclaims and grounds for more comprehensive and thoughtful evaluation and analysis. The most important concern is the human baseline used to make the claims - the cohort is very small (N=6) and of varied experience (four residents, two specialists). Residents especially are in their early career and are unsurprisingly going to have variations in performance. It is not representative of the emergency physicians or experienced general internists who would typically handle such cases in actual hospitals.

This methodological choice, while providing a "challenging comparative standard", does not allow for generalizable claims about surpassing "human physicians." The abstract should be reworded to "outperformed a cohort of six physicians in a simulated EHR environment" instead of "significantly outperforming six human physicians." The work is also missing some important references.

More detailed feedback, opportunities for improvements and additional questions below.

Detailed comments:

“Crucially, these physician actions must adhere to standards such as the Fast Healthcare Interoperability Resources” - this statement seems to imply physicians need to comply with FHIR, however thats the responsibility of health systems / care organizations. Physicians' job is primarily to provide care and they do not directly interact with or need to understand the technical specifications of FHIR. Suggest rewording this.

“First, there is a substantial gap regarding the integration of AI agents into existing workflows of healthcare professionals within real-world hospital environments. ” - The recently released HealthBench dataset also aims to address this. It would be good to include a comparison and benchmarking with AIDOC on this dataset.

“Second, AI agents so far have not been evaluated through comprehensive, end-to-end patient care scenarios encompassing patient communication, diagnostics, therapeutic decision-making, and admission processes. Our study addresses these gaps by evaluating an LLM agent in realistic, longitudinal clinical scenarios that incorporate system integration, therapeutic decision-making, and prior patient context” - The paper is an important citation over here which addresses many of these challenges too. It would be good for the authors to cite and compare and contrast their effort with this - Towards Conversational AI for disease management - https://arxiv.org/pdf/2503.06074

“ AIDOC is built to navigate all options available to practicing physicians while maintaining compliance with industry standards, including FHIR (Fast Healthcare Interoperability Resources) and six medical coding systems” - this is neat!

“We simulate patient interactions through a patient agent, whose responses strictly reflect the documented history of present illness from the dataset.” - While LLM based patient agents are scalable, they are also limited. This makes the task more of an information extraction one rather than truly exploring information acquisition under uncertainty as clinicians have to do. Use of validated patient actors like the AMIE studies maybe a better stress test of the agent capabilities in history taking. I would recommend authors considering this. Similarly, another important missing evaluation is introducing perturbations into the patient agent like new languages, changes in patient personality etc and seeing how robust AIDOC is to this.

“For pancreatic cancer patient cases, AIDOC additionally has the option to read through documents from previous encounters.” - why is this only restricted to pancreatic cancer cases only?

“These physicians interactively reassessed a subset of 311 patient cases comprising all eight target pathologies (cholecystitis (n = 45), pulmonary embolism (n = 45), diverticulitis (n = 45), 114 appendicitis (n = 43 cases), pancreatitis (n = 42), pneumonia (n = 26), and pancreatic cancer (n = 115 21) under conditions identical to those available to the AI agent.” - Did the 6 physicians also interact with the LLM patient simulator agent? Did all the 6 physicians evaluate all the cases?
“it typically begins by assessing patient history, generates a plan, continues with performing a physical 134 examination, adjusts its plan based on new findings” - it maybe important to clarify the AIDOC does not perform an actual multimodal physical exam but rather just simulating it and getting results from the EHR record. Please reword this accurately.

“Decision traces of AIDOC for each target diagnosis. All graphs begin with Clinical History and conclude with Admission.” - The reasoning traces are helpful but I think an important missing part of evaluation is scenarios where no admission is actually needed. This is important to understand and evaluate too.

“To address this limitation, we applied the Tversky distance, which provides an asymmetric measure of similarity that additionally accounts for tests requested beyond those documented in MIMIC-IV” - this procedural alignment metric is interesting!

“Next, AIDOC requested a notably higher proportion of available blood tests, covering approximately 51.1% of those documented in the MIMIC-IV dataset (compared to only 34.6% in our physician study, $P < 0.001$), a result that was consistently seen across all eight diagnoses.” - this is interesting. While good to see, an alternative interpretation is that AIDOC has a lower threshold for testing, which could lead to resource overuse and incidental findings in a real-world setting. This trade-off between thoroughness and efficiency warrants more discussion.

“These results robustly demonstrate that AIDOC matches or surpasses human physicians in selecting clinically relevant diagnostic tests, achieving greater precision and efficiency under identical conditions. Thus, AIDOC effectively mirrors established clinical practice while minimizing unnecessary diagnostics.” - As discussed earlier, these claims will need to be toned or significantly substantiated with additional experiments and results.

“Pre-admission medication prescription however could be an effective measure of this capability, as physicians typically try to avoid altering a patient’s existing medications” - This is interesting. However, if I understood correctly, this is again an a information extraction task from the patient LLM rather than an actual management reasoning and new therapeutic prescription task - which requires substantially complex reasoning. The authors should refer to the AMIE paper linked in bullet point 3 for this.

“When extending our evaluation to these prescription parameters, we observed consistently high performance for AIDOC: dosages matched in 95.6% of times, timing of administration in 92.5%, frequency in 92.8%, and administration route in 96.3%” - The authors should consider reporting error bars for all these results and analysis.

“In direct head-to-head comparisons against human physicians, AIDOC consistently demonstrated superior performance, correctly identifying and requesting 52.8% of relevant procedures across all eight evaluated diseases compared to only 37.0% for human physicians” - similar to comment in bullet point 11, while good to see, it warrants further analysis as again AIDOC may have lower threshold for prescribing procedures or human physicians may actually have failed to interact and elicit full history from the patient agent leading to the cascading failures. Its important to add additional analysis and tone down the claims.

“Our research extends previous work beyond a simple conversational diagnostic process towards autonomously performing a full diagnostic cascade, including laboratory testing and imaging” - The authors should refer and discuss the latest AMIE papers for disease management and multimodal understanding which lead into these aspects.

“who typically conduct initial evaluations and coordinate specialist consultations in emergency department scenarios, this methodological choice provided a particularly challenging comparative standard, demonstrating that AIDOC can perform robustly even against specialist-level expertise.” - As discussed earlier, given the makeup of the human physicians used as comparators in the study, this is an over claim. The authors need to benchmark against more experienced and specialist clinicians and in a panel to make defensible and robust claims.

“This way, our work also advances beyond AMIE, a framework that was primarily designed to support diagnostic decision making and reasoning through direct patient interaction ” - Its important to discuss the limitations particularly of the evaluations of AIDOC. In contrast both HealthBench (as an evaluation of o1) and AMIE are more nuanced and rigorous in terms of the axes and rubrics. Its important to acknowledge that each benchmark tests different facets of medical AI (workflow integration vs. conversational nuance vs. safety rubrics) and position AIDOC's contribution within that context. Similarly there are limited evaluations and discussions of safety and bias of the agent’s responses.

(Remarks on code availability)
Code is not provided at this time and the github URL is unavailable.

Referee #3

(Remarks to the Author)
In this paper, the authors have demonstrated a method by which an LLM-based AI agent can use patient data and routines

from electronic health records to diagnose and order appropriate interventions for a patient. They measure the AI agent's performance against a benchmark (the actual treatment decisions in the MIMIC-IV dataset) and compare the AI agent's performance to the performance of human physicians on the same task. They also evaluated performance with respect to guideline adherence for the relevant diagnoses.

As far as I know, the work is original in its application to clinical workflows, especially medication and lab ordering. It is great to see an LLM applied to clinical tasks beyond diagnosis. This is an activity that can be productized to help clinicians.

My primary critiques of the paper are as follows:

Limitations of the benchmark dataset:
- The authors used a dataset that consists of inpatient admissions at a tertiary care center / academic medical center (AMC). We should be aware that this is not representative of the general population, for example there are a number of patients in the dataset who underwent Whipple procedure for pancreas cancer, and very few centers in the country perform this volume of Whipple procedures.
- The diagnoses for all the patients in the benchmark dataset are known. The authors intentionally removed patients in the MIMIC-IV dataset for whom the diagnosis is ambiguous. However, in real world medicine, the diagnosis is often ambiguous. This omission limits the applicability of these findings to real world medicine.
- All patients in the dataset required admission to the hospital from the emergency department. This introduces bias (for example, not all patients with these diagnoses require admission) and raises the question of how the model would perform on patients who do not require admission.

Definition of ground truth:
- The model, and the physician evaluators, were graded against a ground truth from the MIMIC-IV dataset, which is what actually happened in the AMC. While it's likely the patients in MIMIC-IV received generally good care in the AMC, we don't know whether the ground truth was truly the ideal care. For example, how does the ground truth perform against guidelines?

Physician comparison:
- The task that the physicians performed was not representative of actual medicine. I understand the desire to do an apples-to-apples comparison of AI agent performance vs human performance on the same task, but this was a task that was optimized for an AI agent not a human. We should not conclude from this that the AI agent would perform better given real-world human tasks, which are more than just an EHR interface.
- The physicians recruited for the task have unknown or questionable qualifications. 4 of the 6 are residents (are these trainees in the German context?) and some of these have very short tenure. The methodology does not include any other determination of their quality.

True usefulness of the end-to-end simulation:
- I question why the authors invested effort into developing a conversation simulator and especially the step that requests a physical exam. As a person focused on building AI products with real-world applications, it's important to focus on problems AI can solve well, which means recognizing areas where AI will continue to rely on humans for the foreseeable future. This is more a personal philosophy than a methodology critique, but the AI agent built here would not be productized as an end-to-end solution -- however there are some useful components. The most useful components to me are: medication ordering, lab ordering, and a "diagnosis assist" capability that can help a doctor think through the patient's presentation and make the best plan. As the authors pointed out, doing a reconciliation of pre-admission medications and ordering these is a tedious task for physicians and an area where the AI can excel. I found the procedure ordering capability more questionable, since as a surgeon, this is a major step that I need to have conviction on, and I would be performing these procedures hands-on -- but I would love for an AI agent to help me with the case request!

One thing that is a truly useful direction in the methodology described here is that when an LLM is confined to a discrete set of actions, for example it can only order abc medications or xyz labs, this mitigates the tendency of an LLM to hallucinate and (likely) makes the application of AI in clinical medicine a safer proposition.

Lastly, regarding the order of the manuscript, I went to the Methods section before reading the Results. I would recommend re-ordering these, and moving some of the methods-related statements from Results into Methods.

Based on the above critiques, I do believe the abstract and the conclusions over-state some of the findings. I also do not consider this to be a complete "validation" exercise due to issues with ground truth, so I would ask the authors to state their claims about what has been accomplished here a bit more modestly. But overall, I found this paper useful and interesting to read.

(Remarks on code availability)
The prompts are provided, which is appreciated. I did not review them in detail.

Referee #4

(Remarks to the Author)
I co-reviewed this manuscript with one of the reviewers who provided the listed reports.

(Remarks on code availability)
I have reviewed the attachment. The attached code is however not enough to support reproducibility:
Only prompts are shared in the attachment, and the code used to replicate the experiments are not available.

Referee #5

(Remarks to the Author)
I co-reviewed this manuscript with one of the reviewers who provided the listed reports.

Version 1:

Reviewer comments:

Referee #1

(Remarks to the Author)
The authors have made substantial improvements to the manuscript, particularly in expanding the physician cohort to ten evaluators, adding comprehensive safety evaluations, and validating their LLM judges. These additions significantly strengthen the work. However, a few concerns remain that would benefit from additional analysis.

1. Patient Agent Validation and Benchmark Scalability. The authors justify their AI patient simulator over human actors based on the goal of creating a scalable and reproducible benchmark, which is reasonable. However, this design choice requires rigorous validation of the simulator itself. At minimum, the authors should demonstrate that the patient agent (1) does not leak diagnostic information through improper prompts or adversarial queries, and (2) returns consistent, accurate information when queried with semantically similar but differently phrased questions. Without this validation, the claimed advantages of scalability and reproducibility cannot be fully realized. Additionally, the 574-case dataset (311 in human comparisons) remains modest for establishing generalizability, particularly given Referee #3's concern about MIMIC-IV representing a single academic medical center. The authors could leverage their validated LLM-as-a-judge approach—which showed high agreement with physicians—to evaluate AIDOC on several thousand additional cases without requiring more physician time. This would address whether AIDOC's performance generalizes across more diverse presentations, especially those outside MIMIC-IV's distribution and potentially unavailable to GPT-4o during training, strengthening claims about patient-specific care capabilities.

2. Medication Precision and Therapeutic Duplication. The authors argue that recall is the relevant performance target for admission medications because precision would "conflate necessary therapeutic additions with over-ordering." However, recall by definition has no penalty for false positives, and the authors acknowledge that AIDOC exhibits "rare duplicate prescriptions" and "rare instruction errors." Given these documented instances of therapeutic duplication, precision becomes an essential complementary metric to quantify over-ordering systematically. Is there a reason the tradeoff is not of concern here?

3. Ground Truth Ambiguity and Multiple Acceptable Strategies. A critical finding appears in the medication reconciliation results: even after crediting "continue home medications," the board-certified cohort diverged from MIMIC-IV ground truth in 11.5% of decisions. This substantial divergence rate among expert physicians raises two competing interpretations. First, multiple clinically acceptable management strategies may exist for many cases—the authors themselves note that "local formularies and hospital SOPs often influence drug choice and dosing." If this is true, then treating MIMIC-IV as a singular ground truth may inappropriately penalize defensible alternatives and artificially inflate AIDOC's "superior" performance when it simply matches historical documentation patterns. Second, the simulation may have provided inadequate information for physicians to make optimal decisions, which would undermine claims about the benchmark's fidelity despite physicians successfully completing the tasks. At minimum, the authors should explicitly discuss which interpretation they believe explains this 11.5% divergence and how it affects the validity of their performance comparisons.

4. System Naming Conflict. "AIDOC" conflicts with AIDOC, an established commercial radiology AI company with FDA-cleared products. This creates potential reader confusion. The authors should rename the system before publication to avoid these issues.

These concerns do not diminish the technical contributions but highlight gaps between the stated goals of creating a scalable, reproducible benchmark and the current validation. Addressing at least the minimum recommendations would substantially strengthen the manuscript's validity and impact.

Referee #2

(Remarks to the Author)
Thanks a lot to the authors for carefully reviewing my comments and addressing them. I appreciate the effort involved. They have performed significant additional work in response to my comments.

As a summary, the major areas of improvement are:

1. Improving the human baseline representativeness by adding a new cohort of 4 board-certified physicians (7-11 years experience) and re-running the benchmarks. The new data showing AIDOC still outperforms the board-certified group significantly strengthens the work.

2. Reducing over claiming and toning down language appropriately throughout the text.

3. Adding a new dataset of 80 cases (Pneumonia/PE) specifically to test Admission vs. Discharge decisions. The performance of AIDOC in this setting is impressive.

4.Added statistical analysis and error bars to key figures and providing code.

5. The updated references are clear and capture the latest advancements in the field.

6. Added a new experiment simulating pertubations and bias in the patient agent

Overall, this is strong work and I applaud the authors for it. However, my major concerns or point of view on the work remains largely unchanged from the original submission.

1. The study relies entirely on a patient agent constructed from retrospective discharge summaries. Discharge summaries are synthesized, coherent narratives written after the fact. A patient agent grounded in this data effectively acts as a perfect historian — it likely presents symptoms more clearly, linearly, and logically than a real patient would. The agent's performance may be inflated by this leakage of coherence from the retrospective summary.

The field of Health AI has moved to more realistic real world simulations like the AMIE study or even better towards real world deployments like OpenAI's Penda Health work.

In a similar vein, real-world emergency medicine is defined by ambiguity and missing data. Cases which represent that have been excluded in the study.

2. The data reveals that AIDOC orders significantly more diagnostic tests than humans (51.1% of available tests vs. ~28-35% for physicians). While the authors frame this as comprehensiveness, in a real-world health system, this behavior represents scenarios which leads to ballooning costs, incidental findings and harm from unnecessary workups. The paper does not sufficiently penalize the agent for this efficiency failure. An agent that achieves higher accuracy simply by ordering more tests is not necessarily intelligent — it is just expensive. I would have expected more experiments towards this direction including attempts to rigorously analyze, explain and improve the behavior.

3. For an autonomous agent, the safety testing involving ~56 manual cases is limited.

While these would require major changes, there are also some short term simpler improvements,

1. Adding a a brief expanded discussion on how an economic steward sub-agent could be integrated into the AIDOC framework would strengthen the future work section. This can take inspiration from MAI-DxO. While the diagnostic accuracy is high, the costs and efficiency are a concern.

2. Better positioning the work in the spectrum between MAI-DXO, AMIE and OpenAI Penda Health in the intro.

3. More expanded safety testing

I wish the authors the best of luck with the work and applaud them on a fanstastic paper

(Remarks on code availability)
I briefly skimmed the code and it seems to be in order but I have not reviewed in detail.

Referee #4

(Remarks to the Author)
I co-reviewed this manuscript with one of the reviewers who provided the listed reports.

Referee #5

(Remarks to the Author)
I co-reviewed this manuscript with one of the reviewers who provided the listed reports.

(Remarks on code availability)

Version 2:

Reviewer comments:

Referee #1

(Remarks to the Author)
Overall Assessment:

The authors have undertaken substantial additional work that significantly strengthens the manuscript. The patient agent validation experiments are thorough and well-designed, the expanded medication reconciliation analysis appropriately addresses precision concerns, and the thoughtful discussion of ground truth ambiguity is well-supported by literature. The system renaming resolves the naming conflict. I commend the authors for their responsiveness and the rigor of these additions.

Training Data Transparency:
In my previous review, I suggested scaling evaluation to several thousand cases to better assess generalization. The authors have declined this expansion, noting that their current sample size already exceeds related work. This is a reasonable position.

However, this decision makes transparent disclosure of a potential limitation more important: MIMIC-IV is a publicly available dataset (2008-2019) that could plausibly have been included in GPT-4o's training data. While OpenAI does not disclose training sources, readers should be made aware of this possibility when interpreting the performance results.

Suggested addition:

The authors should add a brief acknowledgment in the limitations section that MIMIC-IV is a publicly available dataset that may have been included in GPT-4o's training data, and that training data contamination cannot be ruled out. While this limitation affects benchmark validity broadly (not just this study), readers should be aware that the reported performance metrics may represent an upper bound rather than true generalization to unseen cases.

(Remarks on code availability)

Referee #2

(Remarks to the Author)
The authors have provided a comprehensive and scientifically sound rebuttal. Thanks again for the dilligent work.

1. They addressed the primary concern (the perfect historian bias) with extensive new validation experiments (n=933 audits, adversarial testing) that suggest the agent is robust, even if the simulacrum limitation cannot be fully removed without a prospective trial.

2. The argument on the efficiency critique is addressed by showing the over-ordering is restricted to low-harm blood tests, not radiology.

3. They significantly expanded the safety evaluation to the full cohort, moving beyond the initial small sample.

4. The statistical treatment is appropriate. The use of paired comparisons (McNemar's test) for diagnostic accuracy and Wilcoxon signed-rank tests for resource utilization is correct, with proper adjustments for multiple comparisons (Benjamini-Hochberg).

The manuscript is now strong. I have only minor suggestions for the final version:

1. The discussion regarding the higher lab order rate (51% vs ~30%) is well-handled, but could be strengthened by explicitly linking the proposed economic steward sub-agent to specific high-cost examples from the data (e.g., explicitly contrasting the low marginal cost of extra blood labs vs. the high cost/harm of unnecessary imaging).

2. Ensure the renaming from AIDOC to MIRA is thoroughly applied across all supplementary figures and tables to avoid reader confusion.

While the limitation of using retrospective data remains, the authors have done everything possible in silico to validate their approach. The paper represents a substantial benchmark for the field.

Further demands for real patient validation would likely constitute a new study entirely.

(Remarks on code availability)
I have skimmed over the code but not in detail.

Referee #4

(Remarks to the Author)
I co-reviewed this manuscript with one of the reviewers who provided the listed reports.

(Remarks on code availability)
I have reviewed the code and there is no obvious mistake.

Version 3:

Reviewer comments:

Referee #1

(Remarks to the Author)
No further comments. I thank the authors for their responses over multiple rounds, which has led to a much strengthened manuscript.

(Remarks on code availability)

Referee #2

(Remarks to the Author)
The authors have been very responsive throughout the peer review process and have addressed my remaining minor comments in this final revision.

Specifically, the addition of the explicit contrasting examples in the Discussion highlighting the low marginal cost of incremental blood labs versus the high cost and potential harm of unnecessary cross-sectional imaging helps contextualizes the proposed economic steward sub-agent. This provides a much clearer framework for how resource stewardship should be evaluated in future iterations of medical AI agents.

Furthermore, I appreciate the transparent acknowledgment regarding the potential for GPT-4o training data overlap with the MIMIC-IV dataset (as suggested by Reviewer 1).

While the reliance on retrospective data from a single academic center remains an inherent limitation of this work, the authors have done everything possible in silico to validate their approach, bound their claims and transparently discuss these caveats.

The resulting study establishes a much-needed, reproducible benchmark for evaluating autonomous EHR-integrated clinical AI.

I commend the authors on an excellent and significant contribution to the field. I have no further requests.

(Remarks on code availability)
I briefly skimmed and dont see any major issues

Referee #4

(Remarks to the Author)
I co-reviewed this manuscript with one of the reviewers who provided the listed reports.

(Remarks on code availability)

Open Access This Peer Review File is licensed under a Creative Commons Attribution 4.0 International License, which permits use, sharing, adaptation, distribution and reproduction in any medium or format, as long as you give appropriate credit to the original author(s) and the source, provide a link to the Creative Commons license, and indicate if changes were made.
In cases where reviewers are anonymous, credit should be given to 'Anonymous Referee' and the source.
The images or other third party material in this Peer Review File are included in the article's Creative Commons license, unless indicated otherwise in a credit line to the material. If material is not included in the article's Creative Commons license and your intended use is not permitted by statutory regulation or exceeds the permitted use, you will need to obtain permission directly from the copyright holder.
To view a copy of this license, visit https://creativecommons.org/licenses/by/4.0/

Rebuttal Letter for “*Towards Autonomous Medical Artificial Intelligence Agents*”

Author Responses to the Reviewers:

Referee #1 (Remarks to the Author):

This study presents AIDOC, an ambitious AI agent designed to operate within an EHR-like environment, performing a full spectrum of clinical tasks from patient dialogue to diagnosis and treatment planning. The work is a notable step forward in the field of medical AI, with a commendable focus on building within an interoperable FHIR framework and evaluating a complete, end-to-end clinical workflow.

Author Response: Thank you very much for your summary and positive assessment of our research. We greatly appreciate your recognition of the scope and ambition of *AIDOC*, as well as your insightful feedback and your specific recommendations for improvement. We have carefully addressed all of your points in our revision, which have helped us to further strengthen the manuscript; please see below.

However, while the paper’s aspirations are high, its central claims of "fully autonomous" operation and superior performance over human physicians are not yet supported by the evidence presented. The study is limited by a lack of transparency, an opaque and potentially biased evaluation pipeline, performance metrics that systematically favor the AI, an unrepresentative human comparator group, and an idealized evaluation environment.

Author Response: Thank you for highlighting these important concerns regarding the evidence and evaluation behind our claims that we presented in our study. We have carefully addressed all of your points by incorporating additional evaluations and making adaptations to the manuscript as suggested, in order to strengthen the validity and clarity of our findings.

Strengths of the Study

The authors should be recognized for several significant contributions that advance the field:

FHIR-Based Architecture: The decision to build the agent within a framework compliant with Fast Healthcare Interoperability Resources (FHIR) is a major strength. It demonstrates a clear and practical path toward real-world integration with existing Electronic Health Record (EHR) systems.

End-to-End Agentic Workflow: The study evaluates a multi-step, agentic process that mimics a clinical workflow—from taking a history and ordering diagnostics to planning therapeutics and admission. This holistic approach is a significant advance over prior models that perform only narrow, isolated tasks.

Author Response: Thank you very much for your positive feedback and for highlighting the importance of both our FHIR-based architecture and the end-to-end, agentic workflow. One of our central ambitions was to design an agent that operates within the same technical, procedural, and regulatory architecture as human physicians, specifically to enable realistic benchmarking and future deployment as a virtual AI copilot in clinical practice. Therefore, we deliberately built and evaluated our system under conditions that closely mimic real-world hospital environments. We have addressed all your concerns, please see below.

Critical Flaws and Actionable Suggestions

Despite its strengths, the study suffers from several severe flaws that undermine the validity and generalizability of its conclusions. These are organized below, with specific, fixable suggestions for the authors.

1. Lack of Transparency and Verifiability During Review

The Flaw: The most significant barrier to the credibility of this work is that its core components were unavailable for review. The authors state they "plan to release" the code, but at the time of review, the AIDOC agent, the patient simulator, and the evaluation framework remain a "black box."

The Actionable Suggestion: For the results to be considered verifiable, the authors must provide access to the full source code, evaluation prompts, and data processing scripts.

Author Response: Thank you very much for this important and constructive feedback. We sincerely apologize for the lack of transparency you have experienced during the review process. At the time of submission, together with the manuscript we made available the complete codebase (including the full FHIR environment, the patient agent and AIDOC, data preparation and clinical code mapping scripts, as well as all evaluation, statistical, and visualization files along with comprehensive documentation, troubleshooting instructions, and a patient case example (lung embolism) for reproducing our workflow). We regret that these materials were not accessible to you and we have since communicated with the editorial team regarding this issue and hope that, upon resubmission of the revised manuscript, you will be given full access to all code and materials. Of course we plan to make the code publicly available upon publication. However, at the time of initial submission we chose to submit it alongside the manuscript without open access, as the repository contains README files and documentation detailing our target diagnosis selection, which could compromise blinding and potentially introduce bias if it was accessed by physicians that we now have additionally recruited during the revision process.

We have uploaded a .zip with the exact source code from the initial submission. It contains everything needed to run the AIDOC-Patient agent interaction and to reproduce the evaluations and plots. In parallel we are packaging the visualization scripts for the new and refactored figures from this revision. We will release these, together with the full repository, under the MIT License on GitHub. These visualization scripts do not alter any core methods, data, analyses, or results. The submitted code base remains unchanged. Please feel free to contact the editors in case you do not receive access to the source code.

2. The Evaluation Pipeline is Opaque and Potentially Biased

The Flaw: The study's headline metrics are scored by multiple (≥5) proprietary GPT-4o-based "Evaluators." Since the AIDOC agent itself is powered by the same model family, this creates a significant risk of "style-matching bias," where an LLM may rate outputs more favorably if they mirror its own phrasing and reasoning style. Only the Diagnosis Evaluator is reported to have undergone a human audit; no parallel check is described for the medication, procedure, or guideline judges.

The Actionable Suggestion: To ensure fairness and transparency, the authors should:

Re-score a random 10-20% of cases for the Medication, Procedure, and Guideline-Adherence tasks with board-certified clinicians to validate the LLM judges' accuracy. Alternatively, use a model from a different vendor (e.g., Anthropic Claude) as a neutral adjudicator to show that the conclusions hold.

Author Response: Thank you very much for raising this important point: In response, we have validated every evaluation step that used an LLM-judge (diagnostic accuracy, guideline adherence, procedure matching and admission-medication standardization) with an additional review by a board-certified physician. Across all tasks we observed very high objective physician-LLM agreement and more importantly we saw no directional bias in errors, meaning there was no systematic false favoring or penalizing of AIDOC or human responses among "misclassified" cases (when using the human review as reference). All results and statistical tests are provided in the Supplementary Data Tables and in the new section describing our LLM-as-a-judge evaluation.

Separately, many core metrics did not involve an LLM at all. We used deterministic, rule-based matching wherever possible, including exact matches for laboratory, microbiology, imaging, and medication names/doses (after standardization), with predefined unit and timing conversions applied before comparison. Thank you once again.

3. Performance Metrics Systematically Favor the AI's Verbosity over Clinical Competence

The Flaw: The metrics for medication and procedure matching are designed in a way that creates an illusion of superior AI performance.

Medications (PMMR): The metric only rewards explicit, line-item prescriptions. AIDOC is prompted to list every drug, while human physicians can use the clinically appropriate shorthand "Continue home meds." As the authors explicitly note, this is incorrectly scored as zero matches. The resulting 94.6% vs. 5.1% gap therefore measures compliance with a system prompt, not clinical competence.

Author Response: Thank you for your valuable feedback. In fact, human participants were asked to prescribe the relevant medications rather than simply defer the task with "continue home medications." However, we recognize that in everyday clinical practice particularly in the emergency department it is both common and efficient for physicians to use this shorthand, with the detailed medication reconciliation and prescription typically delegated to the admitting physician or resident upon transfer to the inpatient department. As a result, some participants in our study naturally opted for this approach. We acknowledge this limitation and agree that it should be taken into account when interpreting the results. In response to your suggestions, we have now made the requested changes as indicated below and have adjusted our conclusions accordingly in the manuscript. Thank you again for your helpful input.

Procedures (PMR): For procedures, there is no penalty for over-ordering, unlike for diagnostic tests where a Tversky distance metric was used. The AI is given credit for both exact and "equivalent" codes identified by its sibling LLM evaluator, which inflates its recall score by counting near-duplicates without assessing precision.

Author Response: Thank you for this helpful critique. We addressed it in three ways:

1. Equivalence handling. We have performed an independent evaluation of the ProcedureMatch LLM Evaluator with a board certified surgeon, who showed full agreement (100%) with its "Direct" vs "Equivalent" decisions. One issue is that the same surgical procedure can be coded in many different ways: We have added a deeper rationale and explanations on this aspect in the manuscript (please see "Section 4.8.6 Procedure Evaluations").
2. Penalty for over-ordering. We added a procedure-level Tversky-distance analysis for AIDOC and both human cohorts. False positives are weighted twice as heavily as false negatives, explicitly penalizing over-ordering. AIDOC shows closer adherence to the chart reference than either human cohort (Extended Data Figures 5C and 7C and Supplementary Data Tables).
3. Precision reporting. We now report precision for procedures (per-patient and micro-averaged), alongside recall, to directly quantify any over-ordering.

We hope that these additions fully address your concerns.

Crucially, none of the headline metrics assess safety elements such as drug-drug interactions, renal dosing adjustments, or cumulative radiation exposure.

Author Response: Thank you for emphasizing the importance of explicit safety measures. In response, we have incorporated manual safety evaluations addressing high-severity drug–drug interactions, renal dose adjustments based on current eGFR or creatinine values, allergy–medication mismatches, QT-risk prescribing without documented rationale, potentially unsafe opioid prescribing (e.g., MME/day > 50 or concomitant benzodiazepine use), and therapeutic duplication. Additionally, we assessed whether any of AIDOC's medication prescriptions were considered incorrect / potentially dangerous on a per-patient evaluation level. All evaluations were performed by a board-certified physician with access to the full underlying patient data and the results from AIDOC's interaction with the patient. Within the scope of the safety measures assessed, AIDOC's performance was considered safe and we have added the evaluations together with other measures of safety and robustness (new admission vs no admission experiments and bias mitigation tests) to Figure 5. Please also see the updated Results section. Regarding radiation stewardship, our evaluations show that across the 574 patients, none of the patients had a CT scan ordered twice for the same region by AIDOC. Together, we hope that our new safety evaluations directly address your concerns and demonstrate that, within the assessed domains, AIDOC's clinical decisions were safe across the cohort.

The Actionable Suggestion: To create a fair comparison, the authors must revise their metrics.

Medications: Treat "continue home meds" as a full match or score at the drug-class level. Report precision to penalize unnecessary medication changes.

Author Response: Thank you for this valuable suggestion. In response, we have implemented two changes and clarified why we believe that medication precision is a slightly problematic metric for this task.

First, as you proposed, we updated Figure 3 (and Extended Data Figure 5A) to credit "continue medications" (and similar instructions identified by manual investigation) as a full match for human evaluation. While this does not resolve the separate real-world requirement that another clinician later has to document all pre-admission medications, our results remain largely unaffected, and AIDOC remains more reliable on this task.

Second, to strengthen our results, we now report admission-medication-level micro- and macro-recall for AIDOC and both human cohorts (the initial mixed-experience cohort and the board-certified-only cohort), each under two specifications: strict matching and matching that credits "continue home medications" and synonyms as a full hit. We provide 95%-CIs via bootstrap resampling (k=10,000). Results are presented in Figure 3 and detailed in the Supplementary Tables, with corresponding updates to the Results text.

Regarding your request to report precision to penalize unnecessary medication changes, we believe that precision is not the most appropriate metric as a primary endpoint here. AIDOC is tasked with full ED management and admission medication reconciliation is only one component. The agent must also initiate acute therapies for the primary condition and manage clinically relevant comorbidities (for instance, electrolyte derangements, oxygen supply, hypoglycemia, pain management). Precision would therefore conflate necessary therapeutic additions with over-ordering and mischaracterize correct

patient-level care as error. For this admission-medication reconciliation task, we therefore believe recall is the relevant performance target.

However, to address the core safety concern you have directly, we added a manual safety review by a board-certified physician who evaluated over 450 medications across a subset of AIDOC treatment plans with full access to the underlying patient data (labs, medication lists, imaging, HPI). This review assessed each individual prescription for correctness and key safety metrics (kidney dosage, drug allergies, drug-drug interactions etc.). We have highlighted some of the weaknesses including rare duplicate prescriptions or rare instruction errors in the results section and discussed a possible solution such as drug-checking agents or even deterministic measures such as timeouts or warnings in case of attempts for duplicate orders. These are crucial safety benefits that are only possible when agents operate directly inside the software.

We trust these additions and clarifications address your concern.

Procedures: Report both precision and recall, introducing a penalty for superfluous requests.

Author Response: Thank you for your comment. In response, we have expanded our evaluation to include recall and micro-averaged precision - thus directly penalizing superfluous procedure requests. We report both micro-averaged metrics for AIDOC and human clinicians, and use paired bootstrap (k=10,000) confidence intervals for statistical comparison. These results are presented in new supplementary tables and an Extended Data Figure 8 detailing per-diagnosis performance with 95% CIs for both comparator groups. We believe these additions fully address your concerns.

4. The Human Physician Comparator Group is Not Representative and Untapped for Crucial Feedback

The Flaw: The human comparator cohort—composed of four residents and two subspecialists—is not representative of the expert clinicians who typically manage these acute conditions. Critically, none were board-certified emergency medicine physicians. The finding that AIDOC outperformed this mixed-experience group does not sufficiently support the broader claim of outperforming "human physicians" in this domain. Furthermore, the study misses a key opportunity to gather feedback from clinicians on the simulation’s fidelity.

The Actionable Suggestion: A more rigorous evaluation would involve two steps:

Repeat the human evaluation with a cohort of board-certified emergency medicine physicians to provide an appropriate performance benchmark.

Author Response: Thank you for raising the issue of our physician benchmark. We agree that a benchmark with board-certified physicians is important. Accordingly, we added a second independent cohort of four board-certified physicians (7–11 years’ experience with experience in ED management) and repeated the full evaluation. Their results and comparisons with AIDOC are now emphasized throughout the Results and main figures; the original cohort has been moved to the Supplementary Materials but is included in the main text, so that our evaluations now span a total of 10 physicians. The enlarged analysis confirms our original conclusion: AIDOC’s performance matches, and on several measures surpasses that of board-certified physicians. For context, Germany has no formal board certification in Emergency Medicine. Day-to-day ED care is delivered primarily by residents 2 to 4 years into training, with oversight from board-certified physicians (our novel cohort) in Internal Medicine, Surgery, or Anaesthesiology, etc. Within this context, the initial mix of four residents and two specialists

used in our initial study reflects the typical workforce caring for acutely ill patients in German EDs. We are confident that the additional evaluations that now contain a total of 10 physicians with a cohort of board-certified physicians only address your concerns about representativeness and further substantiate our findings.

Following the evaluation, survey these physicians to assess the simulation's realism. This should include quantitative ratings (e.g., a 1-10 scale on the realism of the patient dialogue, tool availability, and information flow) and qualitative feedback on the most significant differences between the simulation and actual clinical practice. This would provide crucial context for interpreting the performance data.

Author Response: Thank you for the valuable suggestion to survey our physician evaluators on the simulation's realism. We agree that gathering this feedback is important for contextualizing performance data. Unfortunately, retrospectively administering a validated survey to our expanded cohort is not feasible for this revision due to the high risk of recall bias. To address the underlying goal of ensuring our findings are grounded in a clinically plausible environment, we have instead focused our efforts on substantially expanding the manual human evaluation itself, which was the key demand from the reviewers. Our benchmark has now been successfully used by an enlarged cohort of a total of ten physicians, including a new cohort of four board-certified only specialists and one board-certified physician blindly evaluating AIDOCs outputs (medication management, safety, guideline adherence etc) manually. We believe the successful completion of these tasks by a larger and more experienced group of medical experts serves as a strong implicit validation of the simulation's fidelity. This extensive human engagement confirms the environment is realistic enough for physicians to apply their clinical judgment effectively. We have added a note to our limitations acknowledging the absence of a formal survey and plan to incorporate one prospectively in future studies.

5. The Evaluation Environment Lacks Clinical Realism

The Flaw: The study's framework, while applied symmetrically, lacks key elements of clinical reality. When a test is requested that was not performed in the original case, the system returns "N/A." Furthermore, the simulation operates in a static, sanitized environment that ignores temporal dynamics, resource constraints, and data ambiguity. Finally, all encounters map to one of eight single-diagnosis templates, omitting the multimorbid or nonspecific presentations common in emergency medicine.

Author Response: Thank you for raising these points.
We agree that clinical realism is a central requirement for any benchmark and appreciate the opportunity to clarify why the design choices you highlight are, in fact in our opinion, deliberate safeguards rather than oversimplifications. First, we consider returning *N/A* when a test was not obtained in the source encounter as a safety measure: A growing body of work, including Microsoft's recent MAI-DxO framework on NEJM CPC cases tries to let an LLM hallucinate *plausible* results, but in situations that go beyond straight-forward uni-diagnostic patient descriptions, this requires a second-order model that understands medication effects and interactions, comorbid physiology, and it introduces an entirely new evaluation surface that is extremely hard to vet. In a patient who already takes twenty-six drugs (please see Extended Data Figure 4A) even small pharmacodynamic inaccuracies would propagate through the cascade of agent decisions. By surfacing an explicit *N/A* we instead force the agent either to proceed under genuine uncertainty or to choose an alternative diagnostic path, a behaviour that more closely resembles what happens at the bedside when a laboratory machine is down, a sample is hemolyzed or an ultrasound is not available (which implicitly also represents some sort of resource constraints). One important dimension we acknowledge is cost-benefit modeling as a resource constraint. While the integration of explicit budget or cost limits, such as the stewardship sub-agent recently introduced in MAI-DxO, remains an attractive direction for future work, our current focus is different. Given the urgent, global shortage of physicians (often driven by burnout and administrative overload), our primary aim was to demonstrate the patient care benefits that AI agents can bring to strained healthcare systems, rather

than to address the separate, complex question of whether AI can reduce costs compared to human providers.

Also, the scenarios themselves are not single-issue vignettes, but contain multimorbid or nonspecific presentations: Except for the mostly healthy appendicitis cohort, every patient sample can contain the full comorbidity burden the real patient brought to the ED, directly visible in the extensive polypharmacy the agent must reconcile (in some cases 30+ outpatient drugs, please see Extended Data Figure 4). The eight diagnostic labels are used only offline for sampling and later for evaluation and ultimately every hospital admission requires some sort of working diagnosis. However, the interaction environment preserved each patient's full clinical context, including multimorbidity and polypharmacy. Thus, AIDOC was required to reconcile outpatient medications and manage clinically consequential comorbidities when they affected immediate safety or disposition. For example, in one case the agent identified and treated severe hyperkalemia in a patient with diabetes alongside the index condition. While management of concomitant problems was not a predefined primary endpoint, it was captured through manual, patient-level safety evaluation by a board-certified physician, with results reported in Figure 5.

Additionally, temporal dependencies are also preserved: labs, microbiology cultures and imaging return strictly after the agent orders them, allowing the agent to reproduce the history → exam → labs → imaging → therapy cascade highlighted in Supplementary Figure.

We hope that these explanations clarify your concerns.

The Actionable Suggestion: To test the agent's resilience, the authors should conduct a "Wizard of Oz" experiment. A human clinician would "play" the role of the sandboxed EHR, intentionally introducing realistic delays, resource constraints, and actual results for counterfactual requests to assess AIDOC's adaptive reasoning.

Author Response: Thank you for this excellent suggestion. We agree that a "Wizard of Oz" experiment is a powerful method for assessing an agent's adaptive reasoning. However, our primary goal was to create a scalable and reproducible benchmark for end-to-end workflow execution. This methodological choice is consistent with other recent large-scale agent evaluations that rely on controlled simulations to ensure reproducibility across hundreds of cases. A real-time "Wizard of Oz" evaluation would be a significant undertaking, requiring resources and a protocol that would constitute a separate study beyond the scope of this work.

To address the core concern for more rigorous, human-in-the-loop validation, which we agree was the key demand, we have substantially expanded the manual evaluation in our study. We have enlarged our physician comparator cohort to a total of ten clinicians, now including a cohort of board-certified-only physicians, and introduced a new, comprehensive safety evaluation conducted by an independent board-certified physician. We believe these additions directly address your central demand for greater robustness and provide a strong, human-led validation of our agent's performance. Therefore, we have positioned the "Wizard of Oz" experiment as an excellent direction for future research in our Discussion.

Nevertheless, several of the aspects you have requested are naturally already included in our setup, for example natural delays from FHIR resource creation, allocation, result generation, and observation posting (each may take several seconds) and the resource constraints inherent to the dataset.

6. Dataset Limitations: Filtering and Ground Truth Definition Threaten Generalizability

The Flaw: The study's conclusions are built on a narrow and potentially biased data foundation. First, the process of manually curating 574 encounters from the larger MIMIC-IV corpus (exact denominator not specified) raises concerns about selection bias. Second, the study relies solely on the final MIMIC-IV discharge diagnosis as its ground truth. This is a potential limitation, as this diagnosis may be informed by data unavailable during the initial ED encounter, making it an imperfect benchmark for real-time clinical judgment.

The Actionable Suggestion: To address these data limitations, the authors should:

Provide a detailed data selection flowchart (e.g., a CONSORT diagram) and test AIDOC on a sample of excluded cases to evaluate its failure modes.

Author Response: Thank you very much for your thoughtful feedback and for highlighting these important considerations. To minimize selection bias and strengthen the validity of our ground truth, we based our case selection on *both* the initial emergency department (ED) diagnosis and the final discharge diagnosis. This approach ensured that sufficient clinical evidence for the primary condition was available early on. We chose not to rely solely on the discharge diagnosis because as you said this can introduce its biases, as discharge coding may be influenced by administrative or billing priorities rather than true clinical relevance and the patient might have presented to the hospital with a totally different initial diagnosis. Conversely, exclusive reliance on the initial ED diagnosis would also lead to issues, as these early hypotheses sometimes prove incorrect (due to missing information, pending lab or imaging tests), thereby limiting the accuracy of any subsequent evaluation. We have stated this in the manuscript under "Methods: Benchmarking Dataset Curation" (*"This is to ensure the exclusion of cases where patients were hospitalized under a completely different initial working diagnosis due to incomplete or pending diagnostic information or where signs of the disease only occurred during hospitalisation."*). We would also like to explain that the data selection process was only partially manual. The initial step was fully automated, extracting all patients with the relevant ICD diagnoses and merging the necessary tables based on patient IDs (please refer to the Consort Diagram in Extended Data Figure 10, which we have now included). Only following this step, expert physicians conducted an in-depth review of each case, evaluating the available clinical information against the minimum diagnostic requirements as defined by official medical guidelines / peer reviewed articles (as now detailed in the revised manuscript). This process was supported by a custom Graphical User Interface that enabled comprehensive access to all patient data (please see Appendix screenshot). Of the 600 cases sampled for manual review, 574 were deemed evaluable by our reviewers, while 26 were excluded due to incomplete data. For example, some pneumonia cases were excluded when external chest X-rays, available to the treating clinicians (on disk or as written report), were never incorporated into the EHR (the MIMIC-dataset only contains results for in-house procedures etc) - thus failing to meet the required diagnostic criteria, as imaging is essential for a definitive diagnosis of pneumonia as per medical guidelines. This screening ensured that only cases with complete clinical information were retained in the final benchmark. Accordingly, exclusions were strictly due to data-completeness constraints; meaning that evaluating AIDOC or the physician cohorts on cases missing essential clinical evidence would be methodologically problematic.

We have revised the manuscript to clarify these methods and have added a detailed CONSORT diagram (Extended Data Figure 10) to further illustrate the selection workflow. Thank you again for your valuable feedback, which has helped us enhance the clarity and rigor of our study.

Validate the ground truth itself by performing a sensitivity analysis: have a panel of independent emergency physicians establish a consensus diagnosis for a subset of cases based only on the initial presentation data. Comparing AIDOC's accuracy against both the MIMIC-IV diagnosis and this expert consensus would demonstrate the robustness of its performance.

Author Response:
Thank you very much. We have included a board-certified, ED-experienced physician to evaluate around 30% of patient cases from our shared AIDOC-human benchmark for this purpose. This physician had access to the full, initial presentation patient data that was available during our experiments (patient history of present illness, diagnostic test results, pre-admission medication, etc) and evaluated the underlying diagnoses. Overall agreement with the diagnoses recorded in MIMIC-IV was 100% (n=90). Thank you for pointing at this issue, as we believe that this additional post-validation adds even more confidence into our approach and hope that it addresses your concerns. We have updated the relevant sections from the Methods and Results chapters accordingly.

7. The Statistical Approach Could Be Strengthened

The Flaw: For comparing diagnostic test selection, the authors correctly use McNemar's test on a binary outcome. While statistically valid, this approach reduces a richer "count" of missed tests to a simple win/loss/tie, resulting in a loss of information about the magnitude of the difference. Additionally, given the multiple outcomes assessed across the study, the risk of a false-positive finding due to multiplicity is not discussed.

The Actionable Suggestion: To demonstrate robustness, the authors should perform a sensitivity analysis using a non-parametric test on the paired count differences, such as the Wilcoxon signed-rank test. A discussion of potential family-wise error or a sensitivity analysis using a correction (e.g., FDR) would also increase confidence in the reported p-values.

Author Response: Thank you very much for this helpful and detailed suggestion. You are absolutely right that McNemar's test does not capture the magnitude of the difference in missed tests between AIDOC and human physicians, and that multiple testing correction is important for robust inference.

To address your concerns we have added a sensitivity analysis that keeps the full paired count information by applying a two-sided Wilcoxon signed-rank test to the difference in missed items for microbiology, radiology and blood-value selection (physical examination was a binary task so missed / not missed would converge into a count = 1 or count = 0 task). For each task we now report the median difference, the Wilcoxon p-value, and the matched-pairs correlation as an effect-size measure. In addition, we control the study-wide false-discovery rate with the Benjamini Hochberg procedure. You can find all results in the main manuscript and we include all tests in the Supplementary Data Tables (especially Tables 11-14). To improve trust in our results, we have also added error bars (95%CIs) to each relevant plot. Overall, these tests have helped strengthen our results - thank you very much!

Referee #1 (Remarks on code availability):

Code was not provided.

Author Response: Thank you for your comment regarding code availability and we apologize for this. As noted above, we made the complete codebase and all accompanying materials available at the time of submission and have communicated with the editorial team to ensure full access to you upon resubmission.

Referee #2 (Remarks to the Author):

Summary:

The work introduces AIDOC, an autonomous medical AI agent designed to operate within EHRs. The system performs a wide range of medical tasks from within the sandbox context of a virtual EHR

environment - from taking patient medical histories, to ordering tests, to interpreting them and prescribing medications among others. The authors evaluate against retrospective patient cases from MIMIC-IV and AIDOC achieves superior diagnostic accuracy compared to physicians. The authors stress on the performance of medication prescriptions and adherence to medical guidelines.

Clarity and context:

The paper is generally well-written and easy to follow.

Author Response: Thank you very much for your thoughtful summary and for the time and effort you have dedicated to reviewing our manuscript. Below, we have carefully addressed all of your concerns and look forward to your further feedback.

Originality and significance:

The main strengths of the paper are technical and simulation of the end-to-end clinical workflow integration. The interesting parts are the agent setup with tools and mimicking a realistic clinical setup requiring navigating of the many options available to practicing physicians.This could be a potential important benchmark / environment towards building AI agents with real world clinical applicability. There is no code available at this time to inspect and review this part.

Author Response: Thank you very much for highlighting the strengths of our work and for recognizing the value of simulating realistic, end-to-end clinical workflows. We believe that to build truly useful AI copilots for physicians, it is essential to evaluate their performance under the most realistic conditions possible. Regarding the code, we submitted all materials necessary to run experiments and fully reproduce all statistics and visualizations in the manuscript, but unfortunately these were not made available to you during the review process. We have clarified this issue with the editors to ensure that you will have full access to all code and materials for this revision, while we are actively working on making the entire codebase including the latest version of our visualisation scripts publicly available on GitHub.

At the same time, the actual clinical performance of AIDOC appears to be somewhat unclear and lacking compared to deep, nuanced evaluations of AI healthcare agents like OpenAI healthbench and Google AMIE. There are several potential overclaims and grounds for more comprehensive and thoughtful evaluation and analysis. The most important concern is the human baseline used to make the claims - the cohort is very small (N=6) and of varied experience (four residents, two specialists). Residents especially are in their early career and are unsurprisingly going to have variations in performance. It is not representative of the emergency physicians or experienced general internists who would typically handle such cases in actual hospitals.

Author Response:
Thank you very much for your feedback. Below we have addressed all the points you have made.

1) Thank you for highlighting OpenAI HealthBench and Google AMIE. We have now added a dedicated section to the discussion comparing AIDOC to both. As a short note, HealthBench evaluates single/multi turn conversational safety, but is not addressed at agentic diagnostic/therapeutic decision making. Regarding AMIE, there are currently many different variants, the most similar to ours being "*Towards Conversational Disease Management*". While this version of AMIE is intended for clinical decision making, it remains conversational, while in contrast, our intention was to evaluate a system that is specifically engineered for autonomous execution of end-to-end clinical workflows inside a sandboxed EHR, focusing on correct downstream actions (orders, prescriptions, guideline adherence) and patient interaction, because

we are convinced that this is actually the place where AI assistants while finally have to operate in. As such, the two approaches are complementary but take different approaches.

In response to your concerns regarding more nuanced evaluations and taking inspirations from both projects, we have carefully revised our manuscript to now contain a dedicated section on safety and robustness that includes both new experiments (robustness against six different biases; analysis of safety concerns regarding admission versus no-admission recommendations) and an additional evaluation of six safety metrics (for instance kidney dosage adjustments, drug-interactions and individual per-patient-level manual evaluations several hundred medication placements) that were performed by a board certified clinician.

2) We have carefully revised the results- and discussion section, adjusting the wording to avoid over-statement and ensure that every claim we made is strictly limited to the evidence from our experiments. Please see the adjusted manuscript.

3) Thank you for highlighting the issue of clinician benchmarking. For context, in German emergency departments, daily care is primarily provided by residents (often one to three years into specialty training) working autonomously, with attending physicians (certified in Internal Medicine, Surgery, or Anaesthesiology) available for supervision. As Germany lacks formal board certification in "Emergency Medicine," our initial study's mix of four residents and two specialists quite accurately actually reflects real-world situations, though we recognize this may differ internationally and was a limitation of our study so far. Therefore, to address your concerns, we have recruited a panel of 4 board-certified physicians only, each with clinical experience in ED management who re-evaluated the entire dataset independently (Please note that these physicians were still blinded to the data), meaning that we now have a total of 10 physicians to compare AIDOC to. We have carefully updated the Results section accordingly with our new analysis confirming our original finding: AIDOC performs on par with, and in some aspects surpasses, both physician groups. We hope this additional context and the now larger comparator groups including more robust statistical evaluations (please see all Supplementary Data Tables) address your concerns.

This methodological choice, while providing a "challenging comparative standard", does not allow for generalizable claims about surpassing "human physicians." The abstract should be reworded to "outperformed a cohort of six physicians in a simulated EHR environment" instead of "significantly outperforming six human physicians." The work is also missing some important references.

Author Response: Thank you very much for this helpful suggestion. We have revised the manuscript to clarify our conclusions and adjusted the wording in the abstract and throughout the paper. Additionally, we have updated the references to include the most recent publications on agentic decision making (for instance Microsoft's MAI-DxO (https://arxiv.org/abs/2506.22405) and all relevant Google AMIE publications including the two peer-reviewed articles in Nature and their latest preprints). We appreciate your input, which has helped us to further strengthen the discussion of our manuscript.

More detailed feedback, opportunities for improvements and additional questions below.

Detailed comments:

“Crucially, these physician actions must adhere to standards such as the Fast Healthcare Interoperability Resources” - this statement seems to imply physicians need to comply with FHIR, however thats the responsibility of health systems / care organizations. Physicians' job is primarily to provide care and they do not directly interact with or need to understand the technical specifications of FHIR. Suggest rewording this.

Author Response: Thank you very much for pointing out this important nuance. We have adjusted the wording in our manuscript to clarify this distinction - specifically, that it is healthcare systems and organizations, rather than individual physicians, that ensure compliance with FHIR standards. Please see the updated manuscript for the revised text.

“First, there is a substantial gap regarding the integration of AI agents into existing workflows of healthcare professionals within real-world hospital environments. ” - The recently released HealthBench dataset also aims to address this. It would be good to include a comparison and benchmarking with AIDOC on this dataset.

Author Response: Thank you for highlighting the importance of HealthBench. We fully agree that it is an important milestone of LLM evaluation in healthcare. However, we respectfully note that HealthBench does not attempt to evaluate the technical or workflow integration of an agent inside a live EHR / inside a hospital environment at all. Instead, HealthBench is a valuable benchmark that aims to assess conversational safety and helpfulness across multiple axes related to a broad set of general medical questions (not full patient cases). More importantly, HealthBench does not cover agentic tasks (it is conversation only). In this dataset, every conversation ends after a single textual reply, with no facility for ordering labs, placing prescriptions, or updating longitudinal patient state. For your reference we have added one representative emergency-referral item (filtered with a focus on abdominal emergencies - as this was a major focus of our manuscript) from HealthBench so you can see how its format and purpose differs largely from the EHR-centric patient cases used in our study (please see the Appendix at the end of this letter). We have included and discussed HealthBench inside the discussion section of our updated manuscript.

“Second, AI agents so far have not been evaluated through comprehensive, end-to-end patient care scenarios encompassing patient communication, diagnostics, therapeutic decision-making, and admission processes. Our study addresses these gaps by evaluating an LLM agent in realistic, longitudinal clinical scenarios that incorporate system integration, therapeutic decision-making, and prior patient context” - The paper is an important citation over here which addresses many of these challenges too. It would be good for the authors to cite and compare and contrast their effort with this - Towards Conversational AI for disease management - https://arxiv.org/pdf/2503.06074

Author Response: Thank you for this reference. We deeply appreciate the suggestion to address the recent progress of the AMIE system, including both Nature articles and the preprint “*Towards Conversational AI for Disease Management*.” In our opinion, AMIE marks a great progress in advancing longitudinal disease management and bridging diagnostic and therapeutic reasoning. Our approach however builds on these advances by demonstrating not only diagnostic and management reasoning, but also the autonomous *execution* of therapeutic interventions and clinical workflows within the EHR itself. We believe that together, these parallel developments are highly complementary and collectively will shape the future of agentic AI in clinical care. Thank you for highlighting this important context and we have updated the discussion accordingly. Please see our updated manuscript.

“AIDOC is built to navigate all options available to practicing physicians while maintaining compliance with industry standards, including FHIR (Fast Healthcare Interoperability Resources) and six medical coding systems” - this is neat!

Author Response: Thank you very much for highlighting and appreciating this aspect of our work! We have addressed all your remaining concerns, please see below.

"We simulate patient interactions through a patient agent, whose responses strictly reflect the documented history of present illness from the dataset." - While LLM based patient agents are scalable, they are also limited. This makes the task more of an information extraction one rather than truly exploring information acquisition under uncertainty as clinicians have to do. Use of validated patient actors like the AMIE studies maybe a better stress test of the agent capabilities in history taking. I would recommend authors considering this. Similarly, another important missing evaluation is introducing perturbations into the patient agent like new languages, changes in patient personality etc and seeing how robust AIDOC is to this.

Author Response: We thank the reviewer for highlighting the value of using validated patient actors, which represents a gold standard for evaluating conversational history-taking. Our choice of an AI patient simulator was a deliberate methodological decision to enable a scalable and reproducible evaluation of the entire clinical workflow across hundreds of cases. This approach is consistent with other foundational work in building large-scale benchmarks for medical AI agents where consistency is paramount (*AgentClinic*, https://arxiv.org/pdf/2405.07960). A study with human actors would introduce logistical constraints that would limit the scale and statistical power needed to evaluate the downstream agentic tasks, which are the core focus of our paper. This also allowed us to include a larger dataset (574 cases) compared to previous works using patient actors (for instance AMIE used 159 cases only).

To address the key demand for a more rigorous stress test, we have implemented two substantial enhancements. First, as suggested, we conducted extensive perturbation experiments, testing AIDOC against six distinct patient biases to assess its robustness. Among these are biases that mirror standard patient-actor scenarios: healthy denial despite symptoms, catastrophizing with conviction of a far more serious illness (for instance cancer), marked anxiety, and language barriers. Please find the results from these experiments in Figure 5 and the updated results section. Second, and most importantly, we have greatly increased the human oversight in our evaluation pipeline. By expanding our physician cohorts to a total of 10 physicians and adding a detailed, manual safety review by a board-certified clinician, we have strengthened the human-led validation of the agent's overall performance. We believe these additions provide the rigorous stress test that you are seeking for this stage of research, and we have noted the use of human actors as a vital next step for future work in our Discussion.

"For pancreatic cancer patient cases, AIDOC additionally has the option to read through documents from previous encounters." - why is this only restricted to pancreatic cancer cases only?

Author Response: Thank you for this question. In the case of pancreatic cancer, we saw during our manual data review that many patients were referred to the emergency department with written reports from external institutions, such as radiology findings (e.g., identification of a pancreatic mass on CT) or histopathological results following procedures like ERCP. These documents were consistently referenced and included within the final discharge summaries, particularly in the sections describing previous medical history. However, this information was not systematically coded within the structured fields of the EHR, and thus was not generally accessible through the primary dataset tables. To ensure that all relevant clinical context was available for each case, we curated structured summaries from the free-text portions of the discharge letters for the 23 pancreatic cancer cases, consolidating any relevant information that predated admission including external findings and interventions, pathology reports etc. This curation was necessary because such details were not always available in the structured anamnesis or from the patient interview, as patients were sometimes unaware of results communicated directly between institutions. The exact extraction procedure is described in detail in "PatientHistory Tool

Information Generation." This approach was specific to pancreatic cancer due to the unique referral patterns and documentation practices observed during our manual review of these cases. We hope that this clarifies your concerns. We have also addressed this issue in the discussion, referencing the recent AMIE preprint, which also examined the handling of data from previous patient visits.

"These physicians interactively reassessed a subset of 311 patient cases comprising all eight target pathologies (cholecystitis (n = 45), pulmonary embolism (n = 45), diverticulitis (n = 45), 114 appendicitis (n = 43 cases), pancreatitis (n = 42), pneumonia (n = 26), and pancreatic cancer (n = 115 21) under conditions identical to those available to the AI agent." - Did the 6 physicians also interact with the LLM patient simulator agent? Did all the 6 physicians evaluate all the cases?

Author Response: Thank you very much for these questions. Yes, all physicians evaluated the patient cases under exactly the same conditions as AIDOC using the same patient simulator, except that humans used a graphical user interface (GUI, as shown in Figure 13), while AIDOC did not need this and accessed the underlying execution logic of the GUI components directly. Each case was assigned to a single physician and each physician reviewed a disjoint subset, both in the initial six-physician cohort and in the expanded cohort. Pooling outcomes from multiple physicians on the same patient is not realistic in ED practice and would confound downstream metrics, so we preserved a one-to-one mapping between clinician and case. To ensure validity, we increased the physician pool to ten across two independent cohorts and used a much larger dataset than usually common (our evaluation spans 311 cases, nearly twice the AMIE actor-based cohort (159)). We also strengthened the statistical evaluation (Please find all relevant test results in the Supplementary Data Tables). We have updated the Materials and Methods to clarify this and added the new data to the Results section. Thank you again for your helpful questions and for giving us the opportunity to further improve the transparency of our study.

"it typically begins by assessing patient history, generates a plan, continues with performing a physical 134 examination, adjusts its plan based on new findings" - it maybe important to clarify the AIDOC does not perform an actual multimodal physical exam but rather just simulating it and getting results from the EHR record. Please reword this accurately.

Author Response: Thank you very much for paying attention to this. We fully agree and we have rephrased the relevant part in the revised manuscript accordingly.

"Decision traces of AIDOC for each target diagnosis. All graphs begin with Clinical History and conclude with Admission." - The reasoning traces are helpful but I think an important missing part of evaluation is scenarios where no admission is actually needed. This is important to understand and evaluate too.

Author Response: Thank you for raising this important point, which has also been flagged by Reviewer 3. We agree that not all patients with one of the eight diagnoses we tested necessarily require admission (though the individual patients from MIMIC-IV did). Unfortunately however, the MIMIC-IV dataset does not capture relevant information for patients who were discharged home directly from the ED department (for instance, there is no documentation of clinical history, physical examination, etc. which is a purely technical limitation of the dataset). To address your concern, we added a robustness analysis using an additional clinician-constructed dataset of 80 cases (40 pneumonia, 40 pulmonary embolism) derived from MIMIC-IV templates, where full histories, examinations, vitals, labs, imaging, and medications were created / adjusted under board-certified physician supervision and a variant-level ground truth admission vs discharge was assigned. We focused on pneumonia and pulmonary embolism because for both diagnoses exist quantifiable criteria (CURB-65 and sPESI) that are routinely used in the clinical setting as an estimate for whether a patient can safely be sent home or requires in-hospital care. In our experiments, AIDOC achieved perfect recall for required admissions (0 missed admissions) and as

shown in Figure 5C was able to justify its decisions accordingly - with*out* explicit instructions to use either CURB-65 or sPESI scores. The revised manuscript includes these methods and results in our new section on safety and robustness. Please see the updated manuscript, especially Figure 5. Thank you very much - we believe this additional experiment materially strengthens confidence in AIDOC's safety and clinical validity.

"To address this limitation, we applied the Tversky distance, which provides an asymmetric measure of similarity that additionally accounts for tests requested beyond those documented in MIMIC-IV" - this procedural alignment metric is interesting!

Author Response: Thank you very much for your positive comment on our use of the Tversky distance. We believe that this metric offers a more nuanced and clinically relevant assessment of diagnostic decision-making, as it captures both overuse and underuse of tests rather than simply measuring overlap and can penalize excessive testing. We appreciate your recognition of this approach.

"Next, AIDOC requested a notably higher proportion of available blood tests, covering approximately 51.1% of those documented in the MIMIC-IV dataset (compared to only 34.6% in our physician study, $P < 0.001$), a result that was consistently seen across all eight diagnoses." - this is interesting. While good to see, an alternative interpretation is that AIDOC has a lower threshold for testing, which could lead to resource overuse and incidental findings in a real-world setting. This trade-off between thoroughness and efficiency warrants more discussion.

Author Response: Thank you for raising the important trade-off between thoroughness and efficiency. In our study, AIDOC queried 51.1 % of available labs, compared with 28.3 and 34.6 % for physicians, yet still fewer than the near-complete lab panels present in the ground-truth charts. Consequently, AIDOC's absolute test burden remained lower than historical practice while delivering the highest diagnostic accuracy. Recent external work by Microsoft supports this pattern: their MAI-DxO agent reduced diagnostic costs by around 20 % versus physicians while improving accuracy (https://arxiv.org/pdf/2506.22405v1). MAI-DxO achieved this by adding a "Stewardship" role and real-time budget tracking to veto low-value tests. These findings show that a higher lab-coverage rate from an LLM need not equate to indiscriminate ordering and can coexist with cost-conscious care. We have inserted a paragraph in the Discussion clarifying AIDOC's utilisation profile and citing MAI-DxO as evidence that cost optimisation by LLM-Agents while maintaining high diagnostic accuracy is feasible. Introducing a multi-agent concept including cost coverage is a logical next step for AIDOC and is now noted as future work. Thank you again for the helpful feedback, which has sharpened our interpretation of AIDOC's testing behaviour.

"These results robustly demonstrate that AIDOC matches or surpasses human physicians in selecting clinically relevant diagnostic tests, achieving greater precision and efficiency under identical conditions. Thus, AIDOC effectively mirrors established clinical practice while minimizing unnecessary diagnostics." - As discussed earlier, these claims will need to be toned or significantly substantiated with additional experiments and results.

Author Response: Thank you very much for this important aspect. We have carefully adjusted the wording and phrasing of our conclusions throughout the manuscript as you have suggested, and we also expanded our human comparator group to include additional physicians with emergency medicine experience and board-certified specialists (total of 10 physicians; 2 groups - one board-certified only and one-mixed experience group - the former as gold standard, the latter as representative of most German ED situations). Importantly, our key messages remain unchanged. We appreciate your guidance in helping us further strengthen the clarity and robustness of our work.

“Pre-admission medication prescription however could be an effective measure of this capability, as physicians typically try to avoid altering a patient’s existing medications” - This is interesting. However, if I understood correctly, this is again an a information extraction task from the patient LLM rather than an actual management reasoning and new therapeutic prescription task - which requires substantially complex reasoning. The authors should refer to the AMIE paper linked in bullet point 3 for this.

Author Response: Thank you for pointing this out. We agree that recovering the patient’s home medication list has a large information-extraction component, but in our setting it remains far from trivial: the agent must elicit the drug history conversationally, reconcile synonyms and dosages on the fly, translate everything into structured orders compliant with FHIR Medication Request resources, and do so while it is simultaneously pursuing its diagnostic and therapeutic agenda. Beyond extraction, the agent then has to decide based on the evolving clinical picture which additional drugs are required in the current setting - and which of the patients’ home medications should be paused or continued etc.

Regarding the new therapeutic prescription task: Because local formularies and hospital SOPs often influence drug choice and dosing, we judged that a strict one-to-one comparison of in-hospital medication orders (those specific to the current disease leading to admission) would unfairly penalize clinically equivalent medications. Consequently, we evaluate all treatment related to the acute cause of hospitalisation (pneumonia, urinary tract infection, pancreatitis, etc.) under the guideline-adherence metric: This deems a new in hospital prescription correct when the agent (or the physicians in our study) select a drug that is covered by current best-practice guidance, even if it is not the exact drug that was recorded in MIMIC-IV. This also helps us to mitigate another (temporal) bias: the source admissions in the dataset span 2008 to 2019, so some reference orders pre-date newer evidence. By benchmarking prescriptions against the most current guideline releases, we assess the agent’s orders relative to contemporary standards rather than the (historical) best practices captured in MIMIC-IV.
We have clarified this point in the revised manuscript, including an updated discussion of the AMIE system, covering both the two recent Nature publications and the preprint “*Towards Conversational AI for Disease Management*” as you referenced. While AMIE represents an important advance in longitudinal disease management and expands diagnostic reasoning into treatment planning, our work is complementary in that it not only encompasses these elements but also demonstrates autonomous execution of treatment decisions and clinical tasks inside the EHR. We believe that both directions are synergistic and together offer valuable and mutually reinforcing advances for the field. Thank you again for your helpful suggestion, please see the updated discussion.

“When extending our evaluation to these prescription parameters, we observed consistently high performance for AIDOC: dosages matched in 95.6% of times, timing of administration in 92.5%, frequency in 92.8%, and administration route in 96.3%” - The authors should consider reporting error bars for all these results and analysis.

Author Response: Thank you for this valuable suggestion. We now have added 95% confidence intervals to Figure 3C using a patient-level clustered bootstrap with 10,000 resamples. This treats patients as the unit of independence and preserves potential within-patient correlation among medications. We have updated the Results text accordingly and have added CI-bars to all relevant main and supplementary figures, reporting their upper and lower bound values in the Supplementary Data Tables. We appreciate your attention to this matter.

“In direct head-to-head comparisons against human physicians, AIDOC consistently demonstrated superior performance, correctly identifying and requesting 52.8% of relevant procedures across all eight evaluated diseases compared to only 37.0% for human physicians” - similar to comment in bullet point 11, while good to see, it warrants further analysis as again AIDOC may have lower threshold for

prescribing procedures or human physicians may actually have failed to interact and elicit full history from the patient agent leading to the cascading failures. Its important to add additional analysis and tone down the claims.

Author Response: Thank you very much for raising this important point. As for the diagnostic tests, we now report a Tversky-distance metric for AIDOC and both human cohorts that penalizes over-ordering relative to the chart-derived ground truth (shown in Supplementary Data Figures 5C and 7C), alongside additional statistical validations (please see Supplementary Data Tables). Throughout the manuscript, we have also toned down the language: rather than an “AI-versus-human” framing, we try to more convey the message that, based on our findings, AIDOC performs comparably to physicians on the evaluated tasks and that agents in the long term could serve as a practical decision-support partner within routine clinical workflows.

“Our research extends previous work beyond a simple conversational diagnostic process towards autonomously performing a full diagnostic cascade, including laboratory testing and imaging” - The authors should refer and discuss the latest AMIE papers for disease management and multimodal understanding which lead into these aspects.

Author Response: Thank you for your comment. We have expanded the discussion in the revised manuscript to directly address the two recent AMIE papers in Nature, the new AMIE preprint (https://arxiv.org/abs/2505.04653), and Microsoft’s MAI-DxO (https://arxiv.org/html/2506.22405v1). We appreciate you bringing these important works to our attention. All of these projects represent great advances for the field. In our view, a distinguishing feature of our approach is that agents not only generate diagnostic and treatment plans, but also autonomously execute these plans with (live) feedback, fully integrated within established medical software. At the same time, we recognize the major contributions of other projects including advances in multimodality, repetitive encounter management (as in the latest AMIE versions), and multi-agent systems such as MAI-DxO and we believe that these directions are ultimately synergistic and will converge. Please see the updated discussion section in the manuscript for a more detailed comparison.

“who typically conduct initial evaluations and coordinate specialist consultations in emergency department scenarios, this methodological choice provided a particularly challenging comparative standard, demonstrating that AIDOC can perform robustly even against specialist-level expertise.” - As discussed earlier, given the makeup of the human physicians used as comparators in the study, this is an over claim. The authors need to benchmark against more experienced and specialist clinicians and in a panel to make defensible and robust claims.

Author Response: Thank you very much for this valuable feedback. We have revised our claims and conclusions throughout the manuscript to ensure they are more defensible, including the specific passage you referenced above. Most importantly however, we have expanded our human evaluation with a second cohort of four more experienced and only board-certified physicians, who for instance achieved higher diagnostic accuracy than our previous cohort, but was yet below the performance of AIDOC on most of the metrics.
Thus, while our key message remains unchanged, we have rephrased our conclusions to emphasize that AIDOC performs comparably to physicians on the evaluated tasks and may offer valuable support to clinical teams instead of conveying an "AI is superior to humans" - point-of-view. We hope these changes address your concerns, and we kindly refer you to the updated Results and Discussion sections for further details.

“This way, our work also advances beyond AMIE, a framework that was primarily designed to support diagnostic decision making and reasoning through direct patient interaction ” - Its important to discuss

the limitations particularly of the evaluations of AIDOC. In contrast both HealthBench (as an evaluation of o1) and AMIE are more nuanced and rigorous in terms of the axes and rubrics. Its important to acknowledge that each benchmark tests different facets of medical AI (workflow integration vs. conversational nuance vs. safety rubrics) and position AIDOC's contribution within that context. Similarly there are limited evaluations and discussions of safety and bias of the agent's responses.

Author Response: Thank you very much for these comments. In response we have done the following:

1. We have provided a more nuanced discussion on AMIE (conversational diagnostic / therapeutic reasoning), HealthBench (conversational safety) (which both are fantastic contributions) and AIDOC (agentic diagnostic and disease management and as you have mentioned: workflow integration), including strengths and limitations of each approach. Please see our updated manuscript's Discussion, where we highlight and discuss key differences, also including the recently presented work on MAI-Dx from Microsoft Research (differential diagnosis with multiple LLMs).
2. To address your second concern, we have added a new section on safety and robustness of AIDOC, that includes both new experiments (evaluating its diagnostic performance across 6 different biases (480 independent runs)), its capability in recommending admission vs discharge (reaching a recall of 1.00 in two different experiments with N=80 tests in total) and an additional safety evaluation, where agent outputs were rated by a board certified physician across multiple axes (including kidney dosage adjustments, potential of drug interactions, QT-prolonging medication, unsafe prescriptions and patient-level medication evaluations on several hundred drugs). Also at this step, we have performed a validation of our LLM-as-a-judge approach across all the measured metrics (diagnosis, medication reconciliation, guideline adherence, procedure matching). Collectively, these analyses indicated that AIDOC's diagnostic performance remained stable across all six bias conditions, it did not miss any patients requiring admission, and independent physician review found AIDOC's management to be safe within the evaluated domains.

We hope that this addresses your concerns, please see the updated manuscript.

Referee #2 (Remarks on code availability):

Code is not provided at this time and the github URL is unavailable.

Author Response: Thank you for raising this point. As mentioned in our response to Reviewer 1, we provided the complete codebase and all supporting materials at the time of submission. We regret any issues with accessibility and have communicated with the editorial team to ensure that full access will be granted to you upon resubmission including all codes for running experiments and reproducing any statistics and visualisations from our manuscript. We are also actively working to make our code available upon publication, but we decided to submit it with the manuscript without making it open-access at the time of submission, because it also contains Readme's and relevant information on the selection of target diagnoses etc. Keeping it non-public at submission allowed us to add a second clinician cohort during revision while preserving blinding, since they had no access to the repository and were unaware of the underlying patient cases.

Referee #3 (Remarks to the Author):

In this paper, the authors have demonstrated a method by which an LLM-based AI agent can use patient data and routines from electronic health records to diagnose and order appropriate interventions for a

patient. They measure the AI agent's performance against a benchmark (the actual treatment decisions in the MIMIC-IV dataset) and compare the AI agent's performance to the performance of human physicians on the same task. They also evaluated performance with respect to guideline adherence for the relevant diagnoses.

As far as I know, the work is original in its application to clinical workflows, especially medication and lab ordering. It is great to see an LLM applied to clinical tasks beyond diagnosis. This is an activity that can be productized to help clinicians.

Author Response: Thank you very much for this positive summary of our work. In our revised manuscript we have addressed all the points and concerns you have raised, please see below.

My primary critiques of the paper are as follows:

Limitations of the benchmark dataset:

- The authors used a dataset that consists of inpatient admissions at a tertiary care center / academic medical center (AMC). We should be aware that this is not representative of the general population, for example there are a number of patients in the dataset who underwent Whipple procedure for pancreas cancer, and very few centers in the country perform this volume of Whipple procedures.

Author Response: Thank you. We fully agree with your point that Whipple procedures are concentrated in tertiary centers and are not typical of a general ED case-mix. In our cohort, pancreatic cancer constituted only 4% (23/574) of cases by design; the remainder span common ED symptoms and diagnoses across internal medicine and emergency general surgery (urinary tract infection, pneumonia, pulmonary embolism, appendicitis, diverticulitis, cholecystitis, and acute pancreatitis). These seven diagnoses were selected to mirror the high-volume symptom burden that drives ED demand: Abdominal pain (~13 million ED visits), cough (5.98 million), shortness of breath (5.89 million), and fever (5.83 million), all classical entry symptoms for our target conditions. Also, the selected diagnoses themselves are frequent according to data from the CDC (e.g., urinary tract infection and pneumonia caused ~1.66 million and ~1.2 million ED visits in the United States in 2022; diverticular Disease was accountable for around 336,000 ED visits; pancreatic diseases for ~315,000 in 2022; and gallstone disease (cholelithiasis/cholecystitis) was responsible for around half a million of ED cases (all data from the CDC, 2022; *https://ftp.cdc.gov/pub/Health_Statistics/NCHS/Dataset_Documentation/NHAMCS/doc22-ed-508.pdf*). Finally, while “primary cancer” as a principal ED diagnosis is uncommon (pancreatic cancer is a small subset of cancer-related ED visits), we included it deliberately to test model performance on an infrequent, high-acuity condition. Importantly, it was a small fraction of our dataset (only 4%), so that the bulk of our cohort reflects diagnoses that are very common in ED care. We hope that this addresses your concern and we have clarified this rationale in the revised manuscript.

- The diagnoses for all the patients in the benchmark dataset are known. The authors intentionally removed patients in the MIMIC-IV dataset for whom the diagnosis is ambiguous. However, in real world medicine, the diagnosis is often ambiguous. This omission limits the applicability of these findings to real world medicine.

Author Response: Thank you for raising this point. We would like to emphasize that we intentionally did not remove ambiguous cases, but removed those where relevant information was absent due to technical reasons of the underlying dataset. That said, we did not filter out patients because of diagnostic

disambiguation, but we filtered out encounters whose data footprint in the underlying dataset was too sparse to comply with the relevant diagnostic criteria of each disease. This means, we retained patients who arrived with vague chief complaints; however, what we excluded were stays in which the definitive diagnosis emerged only days later (with patients arriving with a totally different chief complaint) and was therefore unsupportable by the first-day labs and imaging that both AIDOC and the clinicians were restricted to. Likewise, for each condition we required the presence of relevant tests that makes the diagnosis even possible (e.g. a chest image for pneumonia or the measurement of Lipase for pancreatitis); this step removes technical gaps such as outside X-rays that were never recorded in the MIMIC-IV dataset (because it only contains those diagnostic procedures that were carried out in-house), rather than removing clinically uncertain or ambiguous cases. Without that safeguard the benchmark would contain records where neither party could meet published diagnostic criteria. During this process, reviewers were aware of the coded hospital diagnosis and performed a backward-feasibility check ("given only day-0 information, could a clinician reach this diagnosis?"). This allowed us to retain cases with vague chief complaints, ensuring technical completeness. Also, neither to humans nor to AIDOC were the diagnoses available as options upfront, but the diagnostic task was completely open-ended. We hope this clarifies your concerns and we have adjusted the methods section accordingly to make this aspect more clear to the readers. Please see also the new consort diagram (that contains the filtering steps during dataset creation, Extended Data Figure 10) and a new figure including word clouds of the major chief complaint symptoms reflecting exactly those that are typically seen in real-world hospital visits according to CDC statistics (please see the answer to your previous point) in the Extended Data Figures.

Most importantly, while inclusion was anchored to one of eight index diagnoses, patients frequently presented with clinically relevant comorbidities that must inform safe management. Although a full evaluation of multimorbidity lies beyond the present scope, we added a patient-level medication review in which a board-certified physician rated each prescription against the complete clinical profile. We have added one example: in a case with concomitant diabetes and hyperkalemia, AIDOC appropriately initiated additional therapies addressing these conditions in addition to the primary diagnosis. This indicates that the agent's recommendations consider the broader comorbidity burden rather than optimizing solely for the index condition.

We hope we have clarified your concern - thank you very much.

- All patients in the dataset required admission to the hospital from the emergency department. This introduces bias (for example, not all patients with these diagnoses require admission) and raises the question of how the model would perform on patients who do not require admission.

Author Response: Thank you for raising this important point. In fact this was also a concern of Reviewer 2. We fully agree that the presence of one of the eight diagnoses alone does not necessarily mandate hospital admission. A limitation of the MIMIC-IV dataset however is that it lacks detailed clinical information for patients discharged directly from the ED, such as history, physical examination findings, and other contextual data. To address this, we constructed an additional dataset of 80 cases (40 pneumonia, 40 pulmonary embolism) based on MIMIC-IV templates. These cases were developed and reviewed under the supervision of a board-certified physician, with complete histories, examinations, vital signs, laboratory and imaging results, and medication data provided. For each case, a definitive admission versus discharge ground truth was assigned. Pneumonia and pulmonary embolism were selected because validated clinical scoring systems (CURB-65 and sPESI, respectively (which were not provided to the model)) allow objective estimation of safe outpatient management versus need for admission and are frequently used by medical doctors in their daily work. We evaluated the performance of the agent in additional experiments, where the model first needed to establish the correct diagnosis, and then, decide between ED discharge or hospital admission. Notably, all other tools and settings

remained unchanged and the task itself remained fully open-ended. In this experiment, AIDOC achieved perfect recall of 1 for both diagnoses (no missed admissions). The corresponding methodology and results are now detailed in the revised manuscript in the new section on safety and robustness. We are confident that this addresses your concerns.

Definition of ground truth:

- The model, and the physician evaluators, were graded against a ground truth from the MIMIC-IV dataset, which is what actually happened in the AMC. While it's likely the patients in MIMIC-IV received generally good care in the AMC, we don't know whether the ground truth was truly the ideal care. For example, how does the ground truth perform against guidelines?

Author Response: Thank you for raising this important point. We deliberately chose not to compare our in-hospital medication prescriptions directly against the MIMIC-IV ground truth for several reasons. First, the MIMIC-IV dataset includes data as far back as 2008, meaning that some medications, in particular antibiotics, may not represent current clinical standards anymore, making them an unreliable baseline for an evaluation today. In addition, prescribing practices can vary significantly due to local expertise, preferences, and hospital SOPs (which might be variants of clinical guidelines), which further limits the generalizability of such a comparison. We have outlined these considerations in our manuscript. Moreover, there is also an inherent risk of incomplete documentation in EHRs, particularly in emergency settings where urgent medications, such as antibiotics, may not always be recorded accurately. As a result, using this data as a baseline for guideline adherence may be problematic.

To address these issues, we chose to base our primary evaluation of medication guideline adherence on the most recent medical guidelines. This approach helps to mitigate the influence of outdated standards, site-specific practices, and documentation gaps. Our results show that AIDOC has good guideline adherence in comparison to both our physician cohorts (Figure 4 and Extended Data Figure 5). Additionally we have performed an independent manual evaluation of guideline adherence and medication safety by a board-certified physician, the majority of which we show in Figure 5 and we have validated our Guideline Adherence Evaluator through an independent validation showing no source-type specific bias (the Evaluator favoring or disfavoring AI-generated content of physician responses).

Nevertheless, in response to your concern, we also quantified guideline adherence in the MIMIC-IV chart-derived treatments (see below). Concordance was generally high for IV fluids and analgesics, but lower for antibiotics. Please be aware that this comparison compares encounters as early as 2008 with guidelines published in 2020 onwards, these results are highly sensitive to temporal drift. We therefore present the MIMIC-IV analysis for completeness but base our primary benchmark on a direct, contemporaneous comparison of AIDOC and physicians against current guidelines, which we believe is the most appropriate and interpretable standard.

MIMIC-IV “Ground Truth” data Guideline Adherence Evaluation.

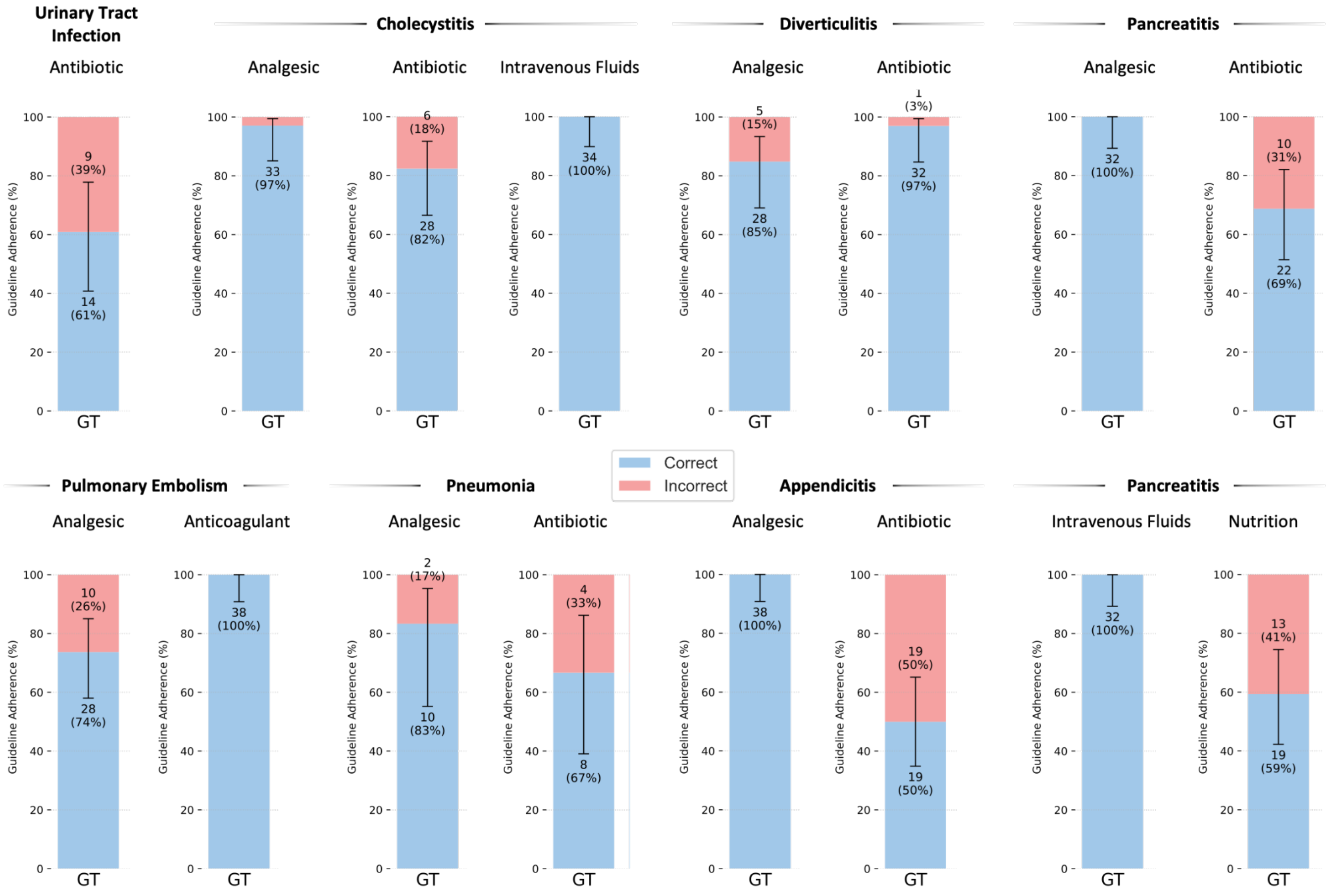

Physician comparison:

- The task that the physicians performed was not representative of actual medicine. I understand the desire to do an apples-to-apples comparison of AI agent performance vs human performance on the same task, but this was a task that was optimized for an AI agent not a human. We should not conclude from this that the AI agent would perform better given real-world human tasks, which are more than just an EHR interface.

Author Response: Thank you very much for your feedback. We fully agree that real-world medical practice consists of far more facets than can be captured in a simulated EHR environment, like direct patient contact, bedside physical examinations, and in-person clinical interaction which are fundamental aspects of human care. Our intention in this study was never to suggest that current AI agents could or should replace these dimensions.

Rather, our study was designed to isolate the EHR-mediated component of clinical workflows, which is where the vast majority of diagnostic, ordering, and documentation actions occur in hospitals, especially in emergency departments where clinicians spend a substantial proportion of their time navigating digital systems (https://pmc.ncbi.nlm.nih.gov/articles/PMC7661623/pdf/11606_2020_Article_6087.pdf).

Importantly, our evaluation was intentionally designed for parity. Both AI and human participants faced the same restrictions: identical tool catalogs (with over 85,000 options), standardized lists for lab and imaging orders like in a point-and-click graphical interface that closely mimics commercial EHRs that physicians use in daily practice. We acknowledge that the Graphical Interface was not used for AIDOC, but the underlying programmatic logic was exactly the same. In fact, we even put stricter constraints on the AI (capping it at 20 conversational turns) while human clinicians had no such limit and could iterate as much as they felt necessary.

Thus, the environment was not artificially optimized for the agent, but rather aimed to replicate the real digital landscape faced by physicians, while holding all else constant. A key novelty of our work herein is that, unlike prior studies which evaluate models only on static recommendations, our study is the first to

require agents to autonomously select and execute all these clinical actions, then respond to the consequences of those actions within a clinical workflow. The agent must iteratively reason, adapt, and update its decisions based on dynamically changing patient data, using the results of previous actions to inform each subsequent step. This goes beyond even the latest iteration of AMIE for disease management, which generates only text-based suggestions. Overall, our approach is very similar to how humans work.

However, we also recognize that EHR interactions are only one part of the clinician’s role. We have carefully revised our manuscript to avoid claiming that an agent would outperform human physicians on tasks that go beyond the evaluation we have performed. Instead, by benchmarking agents and humans head-to-head in a controlled, EHR simulation, we are able to identify the sub-tasks where agents may safely and efficiently support clinicians and we found that they could do so in all areas we have evaluated, especially in documentation-heavy processes like medication reconciliation, guideline adherence checks, and diagnostic ordering. Although our study was an AI versus humans comparison, we have further clarified in the Discussion that we believe that the future of AI in medicine will be fundamentally collaborative, with agents positioned to draft orders, assemble information, and propose recommendations that could remain subject to final human review and approval. The real promise, as our results suggest, lies in freeing clinicians from the most burdensome aspects of documentation and digital workflow, allowing them to redirect their expertise and attention to patient interaction and complex decision-making.
We hope this addresses your concern and have updated the manuscript to more clearly present both the novelty and scope and limitations of our study, as well as our ideas for meaningful AI-human collaboration moving forward. Thank you again for your constructive and insightful comments.

- The physicians recruited for the task have unknown or questionable qualifications. 4 of the 6 are residents (are these trainees in the German context?) and some of these have very short tenure. The methodology does not include any other determination of their quality.

Author Response: Thank you for highlighting the composition of our human comparison group. German emergency departments are staffed very differently from those in many other countries. The vast majority of frontline care is delivered by medical residents who are about two to three years into specialty training; these physicians manage cases independently, while senior consultants are available on call. Moreover, there is currently no board certification in “Emergency Medicine” in Germany. Consultants who supervise emergency care hold specialist recognition in Internal Medicine, Surgery, or, less commonly, Anaesthesiology. Although the creation of a dedicated emergency-medicine board is under discussion, it has not yet been implemented. Against this backdrop, our original cohort of four residents and two subspecialists did in fact represent the standard mix of clinicians who see patients in German emergency rooms.

However we fully agree with you that this might not reflect the situation elsewhere. Therefore, to address your request for a broader benchmark, we have recruited four experienced, board-certified physicians as a separate cohort, in addition to the six doctors who participated originally. We have updated all results and figures in the main text. The expanded analysis confirms our previous finding: AIDOC performs on par with, and in several metrics slightly ahead of, the aggregated human benchmark. We hope this additional context and the enlarged comparator group resolve the concern about representativeness and strengthen the overall study. Thank you once again.

True usefulness of the end-to-end simulation:

- I question why the authors invested effort into developing a conversation simulator and especially the step that requests a physical exam. As a person focused on building AI products with real-world applications, it's important to focus on problems AI can solve well, which means recognizing areas where AI will continue to rely on humans for the foreseeable future. This is more a personal philosophy than a methodology critique, but the AI agent built here would not be productized as an end-to-end solution -- however there are some useful components. The most useful components to me are: medication ordering, lab ordering, and a "diagnosis assist" capability that can help a doctor think through the patient's presentation and make the best plan. As the authors pointed out, doing a reconciliation of pre-admission medications and ordering these is a tedious task for physicians and an area where the AI can excel. I found the procedure ordering capability more questionable, since as a surgeon, this is a major step that I need to have conviction on, and I would be performing these procedures hands-on -- but I would love for an AI agent to help me with the case request!

Author Response: Thank you for this very thoughtful perspective. As also briefly touched in our previous response above, we have revised the Discussion to make clear that we, too, see near-term medical AI agents as virtual assistants operating alongside and under physician supervision, not as stand-alone providers. We now highlight time-consuming administrative tasks, such as reconciling pre-admission medications, assembling lab panels, pre-filling inter-department consultation templates (e.g., automatically notifying the on-call surgery team that a possible surgical emergency has arrived instead of directly requesting a certain surgical procedure X), and drafting guideline-conform orders as the most promising first deployment targets for AIDOC or similar systems. Patient-facing dialogue and the physical examination will, in our view, remain human-led for the foreseeable future, and by using administrative support from agents, physicians might even find more time do to so.
In our benchmark (which is more focused towards research than product development) we nevertheless allowed the agent to gather history and examination findings autonomously so that it faced the same information constraints as a real clinician; most LLM evaluations disclose all facts up front. This design choice lets us test whether such an agent selects sensible next steps while remaining safely sandboxed. Finally, looking beyond academia, patient-facing initiatives such as China's AI "hospital town" (https://med-tech.world/news/china-worlds-first-ai-hospital-milestone-in-healthcare-innovation/; https://arxiv.org/pdf/2405.02957) and early-stage startups like FutureClinic (https://www.futureclinic.com/) show that patient-facing AI platforms are already being trialled. These developments heighten the need for comprehensive benchmarks that include the information gathering task, and we hope that AIDOC can serve as one such testbed. We have adjusted the Discussion to account for our shared perspective.

One thing that is a truly useful direction in the methodology described here is that when an LLM is confined to a discrete set of actions, for example it can only order abc medications or xyz labs, this mitigates the tendency of an LLM to hallucinate and (likely) makes the application of AI in clinical medicine a safer proposition.

Author Response: Thank you for recognizing the safety benefits of limiting the agent to a discrete set of (FHIR-compliant) actions. We fully agree that this design - besides being required to be compliant with EHR infrastructure - also substantially reduces hallucination risk and makes downstream validation far easier. Additionally it also allows for further guardrails, for example, letting the agent auto-populate lab or medication orders while requiring a physician's one-click approval (with the ability to edit all requests prior to confirmation), or adding a "supervisor" agent that double-checks each order (e.g., verifying renal function before accepting a nephrotoxic drug) or even deterministic safety measures such as timeouts for medication orders etc. We believe this collaborative multi-agent, human-in-the-loop workflow can help to maintain full clinical oversight while aiming for high safety. We appreciate your supportive comment.

Lastly, regarding the order of the manuscript, I went to the Methods section before reading the Results. I

would recommend re-ordering these, and moving some of the methods-related statements from Results into Methods.

Author Response: Thank you very much for your thoughtful suggestion. We fully agree that presenting the Methods before the Results is often more intuitive. However, the journal's required structure places the Introduction, Results, and Discussion before the Methods section. To help orient readers, we included a few methods-related details within the Results, aiming to provide clarity about the experimental approach as the findings are presented. We appreciate your feedback and have tried to ensure that all key methodological information is clearly available.

Based on the above critiques, I do believe the abstract and the conclusions over-state some of the findings. I also do not consider this to be a complete "validation" exercise due to issues with ground truth, so I would ask the authors to state their claims about what has been accomplished here a bit more modestly. But overall, I found this paper useful and interesting to read.

Author Response: Thank you very much for your extensive and positive feedback. Following your suggestions, we have rephrased the manuscript accordingly, so that our conclusions always refer to our experiments, without making overstated, generalized claims. Thank you for paying attention to this issue.

Referee #3 (Remarks on code availability):

The prompts are provided, which is appreciated. I did not review them in detail.

Author Response: Thank you for your feedback regarding code and prompt availability. As mentioned in our response to Reviewer # 1, we also provided the complete codebase and supporting materials at the time of submission, but regret that these were not accessible during your review. We have addressed this with the editorial team and will ensure full access to all code and documentation upon resubmission.

Referee #4 (Remarks to the Author):

I co-reviewed this manuscript with one of the reviewers who provided the listed reports.

Author Response: Thank you very much for the time and efforts to review our manuscript. We are confident that we have addressed all your concerns.

Referee #4 (Remarks on code availability):

I have reviewed the attachment. The attached code is however not enough to support reproducibility:

Only prompts are shared in the attachment, and the code used to replicate the experiments are not available.

Author Response: Thank you very much for your thoughtful and constructive feedback. We apologize for the lack of full code accessibility during your review: At the time of submission, we made the entire codebase available alongside the manuscript. This included all scripts for the FHIR environment, patient agent, AIDOC, data preparation, clinical code mapping, evaluation, statistics, and visualizations for all figures, as well as detailed user manuals and an example patient case to allow you to reproduce our experiments. We regret that only the prompts from the manuscript were made available to you and not the full suite of resources. We have coordinated with the editorial team to resolve this issue, and upon

resubmission of the revised manuscript, they will ensure that you have full access to all code and supporting materials.

Referee #5 (Remarks to the Author):
I co-reviewed this manuscript with one of the reviewers who provided the listed reports.

Author Response: Thank you very much for your valuable feedback and for your efforts in reviewing our manuscript. We are confident that we have addressed all of the raised points in our revisions and greatly appreciate your time and contribution to the review process.

Appendix:

Graphical User Interface for Case Selection during Benchmarking Dataset Creation

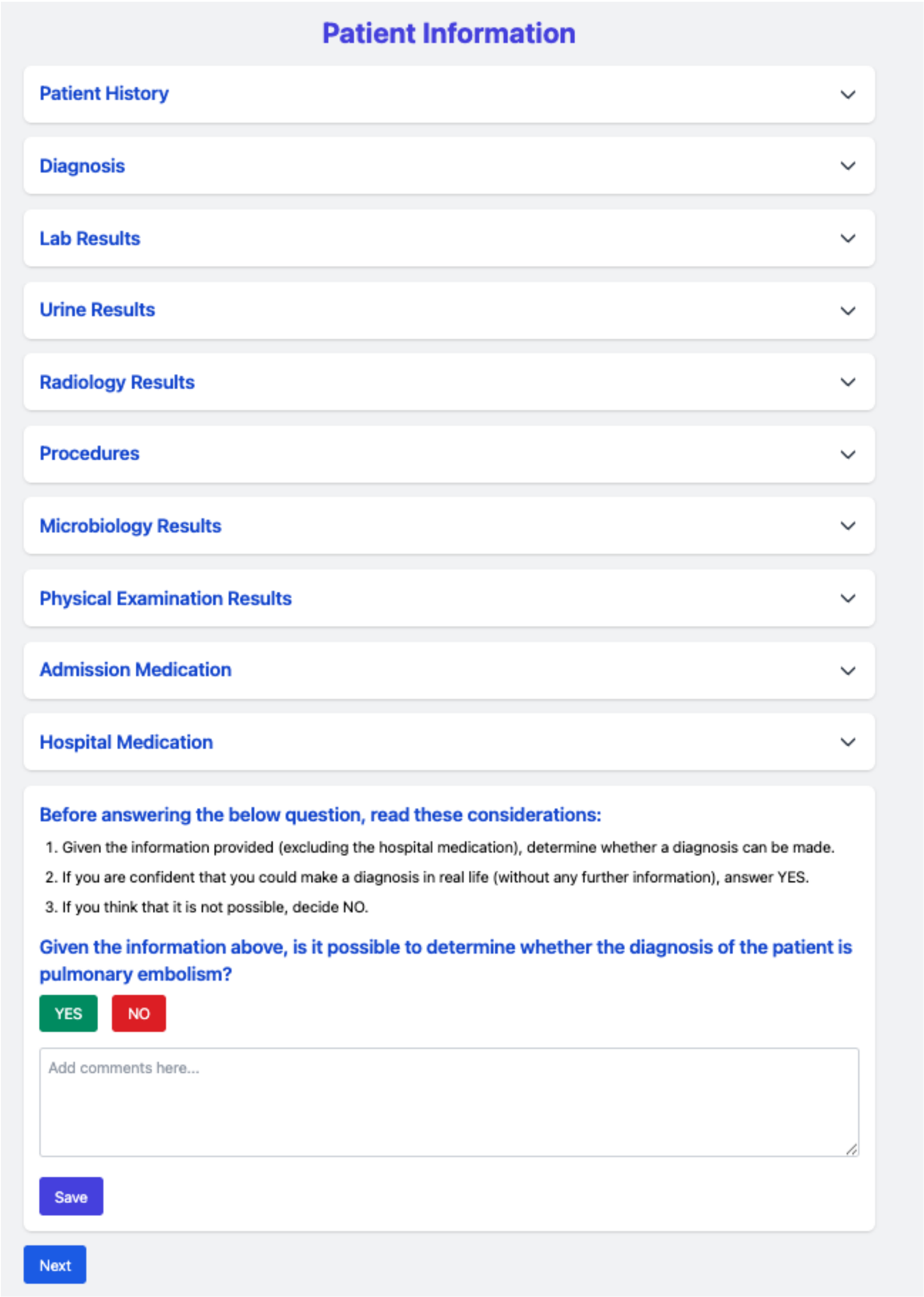

Example Case from HealthBench (https://cdn.openai.com/pdf/bd7a39d5-9e9f-47b3-903c-8b847ca650c7/healthbench_paper.pdf)

Redacted

Rebuttal Letter for "*Towards Autonomous Medical Artificial Intelligence Agents*"

Author Responses to the Reviewers:

Referees' comments:

Referee #1 (Remarks to the Author):

The authors have made substantial improvements to the manuscript, particularly in expanding the physician cohort to ten evaluators, adding comprehensive safety evaluations, and validating their LLM judges. These additions significantly strengthen the work. However, a few concerns remain that would benefit from additional analysis.
Author Response: Thank you very much for your positive feedback and the time and efforts you have taken to review our work. We have addressed all your remaining concerns in our revised manuscript, please see below.

1. Patient Agent Validation and Benchmark Scalability. The authors justify their AI patient simulator over human actors based on the goal of creating a scalable and reproducible benchmark, which is reasonable. However, this design choice requires rigorous validation of the simulator itself. At minimum, the authors should demonstrate that the patient agent (1) **does not leak diagnostic information through improper prompts or adversarial queries**, and (2) r**eturns consistent, accurate information when queried with semantically similar but differently phrased questions**. Without this validation, the claimed advantages of scalability and reproducibility cannot be fully realized. Additionally, the 574-case dataset (311 in human comparisons) remains modest for establishing generalizability, particularly given Referee #3's concern about MIMIC-IV representing a single academic medical center. The authors **could leverage their validated LLM-as-a-judge approach—which showed high agreement with physicians—to evaluate AIDOC on several thousand additional cases without requiring more physician time.** This would address whether AIDOC's performance generalizes across more diverse presentations, especially those outside MIMIC-IV's distribution and potentially unavailable to GPT-4o during training, strengthening claims about patient-specific care capabilities.

Author Response: Thank you for raising these concerns. We agree that a scalable, reproducible benchmark requires careful validation of the *patient agent* itself. Therefore, we performed another manual and AI-supported extensive evaluation of the patient agent, focusing directly on the three main concerns that you raised:

1. Stability under semantically equivalent questioning. We sampled clinician questions from real study conversations (across all three evaluator cohorts (2 physician cohort + the physician agent), generated semantically equivalent rephrasings, and re-queried a *fresh* instance of the patient agent initialized with the identical conversation history up

to that point. Across a total of 622 question-answer pairs spanning all eight diagnoses and all three groups , a physician found the agent's answers to be content-consistent in 99.4% of original vs. rephrased comparisons with high agreement of an independent LLM-as-a-judge with 99.5% agreement to the human physician rater.

2. Faithfulness to the documented history of present illness (HPI): Using the same sampled interactions, we assessed whether both the original and rephrased responses were grounded in the HPI. The patient agent remained HPI-aligned in 99.3% (original answers) and 99.1% (rephrased-answer variants). Again, an independent LLM-as-a-judge produced very similar estimates with high agreement with the physician rater across categories (98.9%, 98.6%).
3. No premature diagnostic information leakage (including adversarial prompting). We audited all 933 conversations (311 encounters x 3 cohorts) for Information Leak, defined as premature disclosure of diagnosis/diagnostic conclusions beyond what a patient would know at ED presentation. We observed 0/933 leaks (CI 0.0–0.4%). We separately tracked prior workup disclosure (clinically plausible information patients already know pre-ED) and found 31/933 (3.3%), concentrated in presentations where prior workup is common (for example, pancreatic cancer) which is a common and realistic setting. To test robustness to hijacking, we applied 11 prompt-injection / social-engineering attack patterns across 80 cases (880 adversarial prompts): again 0/880 premature information leaks, with only prior-workup disclosure observed.

So in brief, we did not observe unwanted information leakage from the patient agent in our experiments.

Also, given the editors' request to place greater emphasis on patient- agent reliability and risk of information leaks, we added a dedicated subsection ("Patient Agent Robustness and Reliability Evaluation") and a new Figure 2 that consolidates and highlights these validation experiments. This figure explicitly positions the patient agent as the backbone of the benchmark, complementing Figure 1 (overall experimental setup) and Figures 3-5 (evaluation of the physician agent and human physicians).

Regarding the suggestion to scale evaluation to several thousand cases, our current sample size (574 cases) already exceeds that reported in closely related work (for example, AMIE[1], n = 159; Hager et al.[2], n = 80 for human- comparison experiments). Nevertheless, to facilitate broader validation, we will release the complete codebase and evaluation pipeline, enabling the community to reproduce and extend our experiments on larger and more diverse datasets. In addition, we are pursuing follow- up work aimed at further assessing generalization beyond the present cohort.

2. Medication Precision and Therapeutic Duplication. The authors argue that recall is the relevant performance target for admission medications because precision would "conflate necessary therapeutic additions with over-ordering." However, recall by definition has no penalty for false positives, and the authors acknowledge that AIDOC exhibits "rare duplicate prescriptions" and "rare instruction errors." Given these documented instances of therapeutic duplication, <u>**precision**</u> becomes an essential complementary metric to quantify over-ordering systematically. Is there a reason the tradeoff is not of concern here?

Author Response: Thank you very much. We agree and therefore have expanded the medication-reconciliation analysis to include micro and macro precision in addition to recall. Precision is reported specifically for pre-admission/home-medication reconciliation, using the MIMIC-IV medication-reconciliation record as reference as this is the clinically appropriate setting for a precision-type metric because the target is a stable documentation task.

To avoid conflating *appropriate* therapeutic additions with false over-ordering, we manually evaluated all medications not present in the MIMIC home-medication list and excluded from the false-positive count (from our agent and our 2 physician cohorts) those representing either disease-directed inpatient therapy (like antibiotics for pneumonia - which we evaluated separately via our guideline adherence evaluation), general inpatient measures including on-demand symptom control (bowel regimens documented “*if needed*”), or appropriate new, acute comorbidity management (like corrections of electrolyte imbalances, oedema). For those drugs classified as acute comorbidity management, we additionally performed a safety evaluation and found no clinical errors (please see below).

Precision was uniformly high across our AI agent and both physician cohorts (close to 100%; please see Extended Data Figure 5C), indicating that mismatches predominantly reflected omissions rather than spurious additions.
We additionally quantified therapeutic duplication across all medication entries (Extended Data Figure 5E) and show that levels of duplication are lower than those reported in real-world investigations on EHR and ED medication lists[3,4]. In deployment, residual duplication risk by an AI agent could be further reduced by deterministic EHR safeguards (ingredient-level duplication warnings and medication-safety checks) with clinician sign-off before orders would be finalized.

We hope that this addresses your concerns. Given the growing set of experiments, and the editorial and reviewer concerns regarding the validity and robustness of the patient agent, we had to add a new Figure and Results section, and have therefore moved the section on admission medication reconciliation, together with a detailed discussion of the performance differences (please see your next point), to the Supplementary Information and Extended Data Figures 4 and 5.

3. Ground Truth Ambiguity and Multiple Acceptable Strategies. A critical finding appears in the medication reconciliation results: even after crediting "continue home medications," the board-certified cohort diverged from MIMIC-IV ground truth in 11.5% of decisions. This substantial divergence rate among expert physicians raises two competing interpretations. First, multiple clinically acceptable management strategies may exist for many cases—the authors themselves note that "local formularies and hospital SOPs often influence drug choice and dosing." If this is true, then treating MIMIC-IV as a singular ground truth may inappropriately penalize defensible alternatives and artificially inflate AIDOC's "superior" performance when it simply matches historical documentation patterns. Second, the simulation may have provided inadequate information for physicians to make optimal decisions, which would undermine claims about the benchmark's fidelity despite physicians successfully completing the tasks. At minimum, the authors should explicitly discuss which interpretation they believe explains this 11.5% divergence and how it affects the validity of their performance comparisons.

Author Response: You raise an important concern, and we agree that the medication- reconciliation comparison can be misinterpreted if it is framed as "*choosing the optimal therapy*" against a single *ground truth*. Our interpretation is different, and we have revised our manuscript accordingly. Here is what we did:

1) What we believe explains the physician–MIMIC mismatch (regarding your two interpretations). In this experiment we evaluated home- medication reconciliation as a structured documentation task (reconstructing and explicitly charting the pre- admission regimen), rather than inpatient therapeutic optimization. In our view, the dominant explanation for the "performance" difference is *workflow* + *documentation trade- offs* from real world experience in physicians, not multiple equally valid inpatient management strategies (We would have seen this in our re-labeling of the "false" positives, where we accounted for synonyms / or equally valid drug substitutions, as described above). We have performed a literature search and several publications and institutions provide evidence on our explanation: At first contact (especially in the ED) medication reconciliation is widely recognized as difficult and time- consuming, and it is often distributed across clinicians, nursing, and pharmacy workflows[5]. The Joint Commission on Accreditation of Healthcare Organizations explicitly notes that obtaining complete medication information at encounters can be difficult and that a "good faith effort" may be sufficient[6]. The WHO similarly emphasizes that medication discrepancies at transitions of care are pervasive and affect nearly all patients across care settings. Empirically, discrepancies at admission are common: in a prospective admissions study, 53.6% of patients had at least one unintended discrepancy and assembling the best- possible medication history required substantial time (median 24 minutes)[7]. In the ED specifically, medication reconciliation competes with acute diagnostics and treatment: a time- and- motion study found physicians spent a median of only 2.2 minutes per hour on medication- reconciliation tasks[8]. These findings support the interpretation that incomplete "early" documentation can reflect realistic prioritization and division of labor rather than lack of clinical competence and reflects real-world experience of work prioritization of the physicians in our experiments.

2) Why we think "insufficient information in the simulation" is unlikely. You correctly note that inadequate information would undermine benchmark fidelity. We consider this unlikely in our setup because the same home- medication information was available to both physicians and the physician agent via the patient- agent dialogue, and we additionally credit "continue home medications" directives to reflect common real- world documentation shortcuts. While we cannot fully rule out that some physicians would obtain additional *collateral* sources in real practice (pharmacy records, prior notes, photos of medication product packages shown by the patient on their smartphones), the simulation was designed so that the relevant home- med list (as was reported by the "real" patient) could be obtained from the patient agent *equally* by both groups, making a systematic information disadvantage an unlikely explanation for the observed documentation gap.

3) How this affects the validity of "performance" comparisons (and what we changed).
Because this task (contrary to the others) is primarily an *administrative documentation bottleneck*, we agree that it should not be presented as *"AI clinically outperforms physicians"*. We therefore revised the manuscript to explicitly state that this evaluation measures documentation completeness, not clinical performance, and that the observed human - AI reference differences likely reflect real-world ED workflow/time trade- offs made by the physicians rather than lower performance in clinical decision making. We also rephrased the interpretation to

emphasize the practical implication: an EHR- integrated agent could draft a structured home- medication list (and propose continue or hold entries) for clinician review and approval, potentially reducing documentation burden and mitigating known discrepancy risks. This aligns with established medication reconciliation quality- improvement guidance that emphasizes multidisciplinary workflows and acknowledges the time intensity of best- possible medication histories[9].

4. System Naming Conflict. "AIDOC" conflicts with AIDOC, an established commercial radiology AI company with FDA-cleared products. This creates potential reader confusion. The authors should rename the system before publication to avoid these issues.
Author Response: Thank you very much for this hint. We have renamed our tool throughout the manuscript and the figures.

These concerns do not diminish the technical contributions but highlight gaps between the stated goals of creating a scalable, reproducible benchmark and the current validation. Addressing at least the minimum recommendations would substantially strengthen the manuscript's validity and impact.
Author Response: Thank you once again very much for your feedback. We hope that our extended evaluations have addressed all of your remaining concerns.

Referee #2 (Remarks to the Author):

Thanks a lot to the authors for carefully reviewing my comments and addressing them. I appreciate the effort involved. They have performed significant additional work in response to my comments.

As a summary, the major areas of improvement are:

1. Improving the human baseline representativeness by adding a new cohort of 4 board-certified physicians (7-11 years experience) and re-running the benchmarks. The new data showing AIDOC still outperforms the board-certified group significantly strengthens the work.

2. Reducing over claiming and toning down language appropriately throughout the text.

3. Adding a new dataset of 80 cases (Pneumonia/PE) specifically to test Admission vs. Discharge decisions. The performance of AIDOC in this setting is impressive.

4.Added statistical analysis and error bars to key figures and providing code.

5. The updated references are clear and capture the latest advancements in the field.

6. Added a new experiment simulating pertubations and bias in the patient agent

Overall, this is strong work and I applaud the authors for it. However, my major concerns or point of view on the work remains largely unchanged from the original submission.

Author Response: Thank you so much for appreciating the work we have done in our last revision and acknowledging the improvements. We have added new experiments and evaluations that should address your remaining concerns - please see below and our updated manuscript.

1. The study relies entirely on a patient agent constructed from retrospective discharge summaries. Discharge summaries are synthesized, coherent narratives written after the fact. A patient agent grounded in this data effectively acts as a perfect historian — it likely presents symptoms more clearly, linearly, and logically than a real patient would. The agent's performance may be inflated by this leakage of coherence from the retrospective summary.

Author Response: Thank you so much for this challenging concern! We appreciate this is an important aspect and we address it in several complementary ways and have clarified this more explicitly in the revised manuscript.

1) What our "patient agent" actually sees (and what it does not see). Our patient agent is *not* given the full discharge summary or the hospital course. It is grounded only in the documented history of present illness (HPI) extracted from the record, with explicit instructions to (i) *not reveal diagnostic conclusions* from future timepoints, and (ii) not invent information beyond the provided HPI. Overall, the dialogue does not provide an upfront vignette; information is revealed only when elicited by the physicians / physician AI agent through questioning (mirroring a "structured history-taking" process rather than a monologue).

2) We directly tested (and did not observe) diagnostic "answer leakage". To ensure the patient agent does not shortcut the task by inadvertently disclosing information, we added a dedicated evaluation of information leakage and robustness. Across all 933 encounters (311 cases x three experimental groups), we observed 0/933 "information leaks" (primary endpoint: premature diagnostic disclosure). These evaluations were done by an automated AI judge and a human physician independently with very high levels of agreement. We additionally stress- tested the patient agent with 11 adversarial prompt- injection / social- engineering patterns across 80 cases (880 prompts) and again observed 0/880 information leaks. These results support that the agent is not "giving away" the solution.

3) We argue that "Perfect historian" is not an objective baseline because real ED histories can vary and are strongly clinician- shaped. We agree that discharge summaries might arguably appear more coherent than raw patient speech. After we performed an extensive literature research trying to find evidence on *how* coherent real patient speech is, we have to conclude that the major problem is that there is <u>*no*</u> *accepted quantitative metric* for how *"clearly, linearly, and logically"* real ED patient narratives are. Importantly, real ED histories are not purely patient- driven: classic ED communication studies show that patients are often interrupted very early (for instance, they speak only around 12 seconds on average before being interrupted and only approximately 20% complete an opening statement[10]. These findings support the broader point that ED histories are co-constructed through clinician questioning rather than delivered as a linear single-shot monologue.
Moreover, prior work on physician-patient interaction including studies using the Roter Interaction Analysis System (RIAS)[11], has investigated what kinds of contributions patient statements make during ED encounters by classifying *utterances* (standalone chunks of statements) into functional categories (for instance biomedical/psychosocial information-giving).

However, this literature is not designed to provide a universal “coherence” benchmark: studies are typically single-center with limited sample sizes, span different clinical problems than ours, and results vary substantially between publications. Therefore, these results are informative qualitative anchors but cannot serve as a definitive quantitative ground truth for how “clear/linear” patient narratives should be.

Nevertheless, to provide a pragmatic internal check, we adopted a RIAS-inspired utterance-level analysis in our own setting: we segmented simulated conversations into utterances using RIAS and retrospectively labeled them as diagnostically contributory versus non-contributory relative to the final diagnosis. This analysis indicates that diagnostically relevant information is not confined to the earliest turns but can occur throughout the dialogue, consistent with the iterative nature of history-taking (e.g., late clarification of risk factors, medication history, or associated symptoms). We emphasize that this analysis has limitations: contributory versus non-contributory labels do not have an external ground truth, depend on the target diagnosis and surrounding setting, and are influenced by the clinician’s questioning strategy. However, from observations we can say, that these conversations do not appear linearly/overly coherent regarding the medical information that is provided by the patient agent. We have added this with a detailed discussion into the Supplementary Information and as Extended Data Figure 16+17.

Moreover, because we plan to open source our full framework, we hope this preliminary analysis will enable the community to more systematically study and benchmark communication patterns in patient–physician agent simulations (including varying degrees of conversational noise and different clinician questioning styles) under controlled, reproducible conditions.

4) Our setup is aligned with (and extends) the strongest available simulation setups. Many recent end- to- end clinical decision-making benchmarks necessarily rely on curated EHR narratives for scalable, controlled evaluation (e.g., the MIMIC- CDM[2] framework presents models with the full HPI directly (and does not contain synthesized patient Q&A from it); AgentClinic[12] also uses simulated patient agents). Our contribution is to go beyond simply using such narratives by adding explicit patient-agent reliability and leakage evaluations and by benchmarking against two human physician cohorts under identical conditions.

As another option to agent simulators, human patient actors like in AMIE could be considered: However, it is important to recognize that patient actors themselves may also introduce certain limitations regarding realism. Importantly, our intention is not to critique AMIE's approach, rather, we thoroughly explored various alternatives to address your concerns and found that standardized patient actors, even in realistic scenarios do not resolve the realism issues you raised. In AMIE for instance, patient actors are *“comprised of a mix of medical students, residents and nurse practitioners”*[1], which arguably could also be “more clearly, linearly, and logically than a real patient” would be. Moreover, the used OSCE formats in particular are explicitly designed to reduce *variability* from real world encounters and also rely on prewritten scenario packs (that contain the original diagnosis[1]) and that in some setups are visible to the actors throughout the conversation[13]. In addition, some work shows standardized patient portrayals are not perfectly accurate and can differ meaningfully from real patients, while being rated less authentic by medical students[14]. Therefore, while actor-based evaluations could be in our opinion a valid alternative, they are also not a perfect antidote to “coherence leakage” concerns; rather, they represent a different realism-to-control trade- off.

5. No unfair advantage in our head- to- head comparisons. Even if one assumes residual "coherence" in the HPI makes the absolute task easier than real-world encounters, this affects the physician agent (MIRA) and human physicians *equally*, because all three cohorts interacted with the same patient agent and the same EHR tools under identical constraints - and we have shown that answers by the patient agent are highly consistent to different ways of interacting with it. Consistent with this, performance remains non- trivial for both humans and MIRA on harder conditions (e.g., pneumonia and urinary tract infections), which argues against the histories functioning as "give- away" solutions.

6. Most importantly: no performance degradation in the bias experiments. One could consider our bias experiments as a kind of approach to perturbating the suspected linearity in the patient agent, because the patient agent was instructed to suspect other diagnoses (e.g., being convinced to be completely healthy or having cancer), or to show different emotions (for example being overly anxious). However, we did not see a performance degradation of MIRA (previously AIDOC).

7. Scope and future work. We believe that the *ultimate* definitive test will be prospective evaluation (or standardized-patient studies) in constrained real workflows. We now emphasize this limitation more explicitly in our manuscript. However this would require long term evaluation under conditions similar to a clinical trial and was therefore unfortunately out of scope for our current work.
However, we note that releasing the full source code of our framework will enable the community with more systematic follow- up experiments that dial history realism/noise up or down (e.g., disfluency, omissions, inconsistent answers) while keeping the EHR action space and safety constraints unchanged.

In summary, our patient-agent design is best viewed as an *EHR- grounded, diagnostic and therapeutic simulation*: controlled for reproducible benchmarking of autonomous EHR agents, explicitly tested to avoid diagnostic leakage, and used identically for MIRA and the physician baselines. From everything we have tested and compared to existing literature, we did not see that the patient agent would speak overly linear/logically as suspected. We have nevertheless added your point to the main manuscript and added extensive discussion on this in the Supplementary Information.

The field of Health AI has moved to more realistic real world simulations like the AMIE study or even better towards real world deployments like OpenAI's Penda Health work.
Author Response: Thank you! We fully agree that both AMIE and OpenAI's work with Penda Health are both two very exciting areas of research and deployment.

We have followed your suggestion (#2 below) and revised our manuscript to more explicitly position our work relative to these lines in both, early in the introduction and the discussion and outlook. In brief, AMIE's two Nature studies focus primarily on conversational diagnostic performance and consultation quality in OSCE-style settings, and more recent work extends AMIE toward a guideline-grounded disease management tool[15]. In contrast, the OpenAI + Penda Health AI system represents a real-world EHR-integrated deployment, but operates as a clinician safety net: it flags potential issues while leaving all clinical decisions and actions to clinicians. We believe that our work is complementary: we study an autonomous agent oper-

ating within a sandboxed EHR environment that translates model outputs into structured clinical actions across full emergency-department care episodes. We view this *EHR action* capability as a missing bridge between high-quality conversational systems and real-world deployment, and we emphasize that prospective, clinician-in-the-loop evaluations remain essential next steps for safety and governance before any autonomous use in practice. For example, complementary to the OpenAI + Penda Health tool, a physician-in-the-loop variant of our agent could function as an "action safety net": it could directly propose (and automatically preselect a small set of additional, useful laboratory tests based on the clinician's current orders (for example to avoid missing an important differential diagnosis) or pre-fill medication dose adjustments (for example, if a reduced renal function was overlooked), flag this to the physician with a warning while waiting for their explicit review and approval before any order is finalized.

In a similar vein, real-world emergency medicine is defined by ambiguity and missing data. Cases which represent that have been excluded in the study.

Author Response: Thank you very much for sharing this concern. We agree with you that real-world emergency medicine is characterized by ambiguity and incomplete information, and we explicitly aimed to retain this property in our benchmark. Importantly, we did not exclude cases merely because they were *messy* or diagnostically challenging, nor did we require complete documentation across all modalities. Instead, we applied a minimal evaluability filter (collected from official medical guidelines) to remove a small subset of encounters in which crucial diagnostic evidence was *technically* absent from the MIMIC-IV EHR extract, for example data that would likely have been available to the treating team in reality but was not captured in the in-house EHR data that MIMIC-IV was derived from.
Including such cases would conflate our agents and also physician's performance with *technical* dataset incompleteness, because neither our agent nor the physician comparators could access the missing information in the sandboxed EHR environment.

To give you an example, exclusions were driven by situations like:
Transferred pneumonia cases with external chest imaging not present in the in-house EHR extract. For pneumonia, chest imaging (CXR or CT) is a common step for diagnosis; in some real ED transfers, clinicians have an outside image and written radiologist report (printed or sent via non-integrated systems), but since MIMIC-IV is built from the destination hospital's EHR, the external imaging report never appears in the structured radiology tables or accessible notes. In our sandbox, this would mean the clinicians and the agent cannot retrieve any chest imaging despite it being clinically available at the time (in reality), thus making the case non-evaluable in our simulation.
This filtering step affected only 26 out of 600 physician-reviewed candidate cases (1 pancreatitis, 1 appendicitis, 3 UTI, 6 pneumonia, 15 cholecystitis) based on an agreement vote by two experienced physicians about the missing data.

We have made these details even more explicit in the Materials and Methods Section (Benchmarking Dataset Curation) in the last paragraph and in Extended Data Figure 12.

Crucially, the retained cases continue to reflect the ambiguity and partial observability that define emergency care. As a result, both the agent and the physician comparators routinely operate with heterogeneous and incomplete workups (variable lab panels, microbiology and

imaging availability) and imperfect documentation, rather than being evaluated on artificially *complete* case vignettes.

At the same time, our evaluation pipeline is designed to remain solid under this *missingness*. Each analysis (that is grounded on MIMIC) is restricted to information that is verifiably present and comparable in MIMIC-IV: metrics are computed only on evaluable ground-truth elements (for example, medication dose/frequency/timing/route are assessed only when explicitly documented), and key endpoints are additionally anchored by head-to-head comparisons against physicians under identical information access rather than relying solely on dataset labels. Finally, we also included a scoring with an independent, blinded board-certified physician review of safety-critical outputs (including medication safety screening and prescription correctness using full clinical context), ensuring that conclusions about clinical appropriateness do not hinge on any single structured field being present in the dataset.

Finally, one thing that we noted from your comment is that our sandbox evaluates decision-making based on information available within the EHR record, while in routine ED practice, clinicians may additionally incorporate external artifacts (like scanned referrals, photos, handwritten notes, or outside imaging). This does not affect the fairness of our head-to-head comparison, since both the agent and physicians had identical access in the sandbox and this was only relevant for a very small fraction of cases and solved via the History Lookup Tool in Pancreatic cancer. However, we have included this point now as a future extension of research in the Discussion Section. We hope that this now fully addresses your concerns.

2. The data reveals that AIDOC orders significantly more diagnostic tests than humans (51.1% of available tests vs. ~28-35% for physicians). While the authors frame this as comprehensiveness, in a real-world health system, this behavior represents scenarios which leads to ballooning costs, incidental findings and harm from unnecessary workups. The paper does not sufficiently penalize the agent for this efficiency failure. An agent that achieves higher accuracy simply by ordering more tests is not necessarily intelligent — it is just expensive. I would have expected more experiments towards this direction including attempts to rigorously analyze, explain and improve the behavior.
Author Response: We agree that something like *resource stewardship* is a core requirement for any EHR-integrated agent, and that diagnostic accuracy alone is not sufficient if it is achieved by indiscriminate testing. We would like to clarify what the reported “51.1% vs. 28-35%” comparison captures in our setup, why it does *not* imply runaway real- world utilization, and what we changed / added to address your concern more directly.

1) *What “51.1% of available tests” means in our benchmark (and why it can overstate real utilization differences).*

   Our test-selection metric counts distinct individual blood analytes documented in MIMIC-IV within the first 24 hours (around 36 parameters per case) and asks what fraction of those *individual analytes* each evaluator requested. Importantly, we intentionally did not provide pre-assembled lab panels (CBC/metabolic/coagulation panels, etc.) to either humans or the agent; instead, clinicians had to select from around 250 LOINC-coded analytes one-by-one. This design makes the action space explicit and

prevents "panel clicks" from trivially inflating overlap. Under this granular representation, the absolute difference corresponds to a median of +7 additional analytes per case (Human − MIRA median paired miss difference = +7; IQR +2 to +11 (please see Supplementary Data Table *"Sensitivity analysis: Wilcoxon signed-rank results for paired miss counts on non-binary metrics (MIRA and board-certified physicians)"*), which is closer to "ordering a slightly broader baseline panel" than to an expansive, costly workup.

2) *The agent is not ordering "everything" - and is below real-world (dataset) utilization.* If "available tests" is defined by what was actually obtained in routine practice in MIMIC-IV, then the reference corresponds to 100%. MIRA requests around 51% of that set, meaning it needs less lab values than *historical real-world records* in the dataset rather than exceeding it. So while MIRA is more expansive than physicians *in our granular interface*, it is still not behaving like a "test everything" panel-policy relative to the real-world baseline we anchor to.
3) *The over-selection is concentrated in low-marginal-cost labs, not in the highest-cost / highest-harm modalities.*
We agree with you that the biggest real-world risks of over-testing are driven by expensive, high-incidental-finding modalities (especially imaging) and cascades from low-yield workups. In our study, the "more testing" for MIRA effect is primarily observed for blood analytes. By contrast, for microbiology we did not observe differences, and for radiology the agent did *not* show over-ordering relative to physicians (if anything, discordant-pair analyses skew toward physicians requesting more imaging). Thus, the main cost driver (imaging-driven cascades) is *not* where MIRA is more expansive, which substantially weakens the interpretation that the performance gain is simply buying accuracy with expensive tests. Interestingly Tversky distance (penalizing over-ordering) was closer for MIRA and MIMIC, while recall (how many radiology tests in MIMIC-IV were covered in our experimental groups?) was balanced (slightly skewed towards physicians), meaning that MIRA compared to our clinician achieved same/better diagnostic accuracy with no over-ordering. We have revised the manuscript text to make this penalty and its interpretation more explicit, and we toned down the framing of higher lab coverage as "comprehensiveness" to better reflect the stewardship trade-off.
4) *Why broader lab coverage can be clinically safety-relevant in our setting.* A non-trivial fraction of the additional analytes requested by MIRA (and physicians) are directly tied to *safe downstream actions* (e.g., renal function / electrolytes that govern dosing, QT-risk checks, contraindications, and avoiding unsafe medication combinations). This connects to the strong medication-safety results we report (renal dosing, interactions, allergies, QT/opioid risk). In other words, part of the "extra lab" behavior is plausibly a safety-driven information-gathering strategy rather than indiscriminate searching.

What we changed / added in response to your suggestion on efficiency. We agree that the right next step is to evaluate and optimize agents under explicit resource constraints rather than treating test selection as a secondary outcome. In the revised manuscript we therefore (i) clarify the panel-versus-analyte issue above, (ii) explicitly highlight that the observed difference is primarily in blood analytes and not radiology, and (iii) expand the Discussion to outline

concrete mechanisms for improving efficiency, including a cost-aware supervisory "economic steward" agent (inspired by MAI-DxO) that tracks cumulative costs in real time and can veto low-yield orders or propose lower-cost, guideline-concordant substitutes. In addition, we now emphasize as an additional control step that early real-world deployments could keep all orders in a pending state with clinician sign-off.

3. For an autonomous agent, the safety testing involving ~56 manual cases is limited.
Author Response: We agree that safety evaluation should extend beyond a single manual review subset, and we have therefore further strengthened and clarified the safety evidence.
First, the blinded board-certified physician safety audit remains an important component: he reviewed n=56 patient cases and assessed 468 individual medication orders (home medications at admission plus new inpatient orders) across six predefined high-risk domains (high-severity drug–drug interactions, renal dosing compatibility, allergy mismatches, QT-risk prescribing, unsafe opioid prescribing, and therapeutic duplication). In this manual review, no high-severity drug–drug interactions, renal dosing incompatibilities, allergy mismatches, QT-risk prescribing, or unsafe opioid prescribing were observed; the few flagged issues were limited to duplication/documentation-clarity concerns rather than severe prescribing errors.
In addition, we now explicitly report safety-relevant medication analyses performed at scale across the full head-to-head cohort (n=311 ED encounters for all groups). Pre-admission medication reconciliation showed very high precision (99.6%) for MIRA, indicating that the agent rarely documented medications absent from the reference record, which we view as an important practical safety property. Moreover, we expanded our assessment beyond home-medication documentation to include medications initiated for acute comorbid issues or symptom control during the ED encounter. Across the full cohort, we did not identify severe prescribing errors in these "non-home-med" additions in any group. Where medications were later judged to be non-essential, these were best characterized as conservative or potentially superfluous choices rather than harmful ones. The most common pattern involved empiric antibiotics started in a small number of pulmonary embolism presentations with imaging reports suggestive of pulmonary infarction and concurrent clinical signs of infection (e.g., cough, fever, or inflammatory laboratory signals). In these cases, the decisions were clinically defensible given the information available at presentation and represent cautious management under uncertainty (empirical antibiotics), not clear-cut unsafe prescribing.
We also quantified therapeutic duplication in structured medication documentation and found it to be below reported real-world duplication rates (approximately half of the duplicate-prescription rate reported on printed pharmacy medication lists), supporting that the agent does not increase duplication risk relative to known reconciliation artifacts or to the board-certified cohort in our study.
Finally, the manuscript contains additional safety/robustness stress tests beyond medication ordering: an admission-versus-discharge experiment (n=80 cases) in which recall for admission was 1 in both cohorts, and perturbation robustness experiments comprising 480 paired evaluations (6 bias scenarios * 80 cases), showing stable diagnostic performance under the tested perturbations.
Overall, we hope these additions address your concern by showing that safety testing is substantially broader than the initial n=56 audit and now includes cohort-scale medication safety analyses across all encounters, complemented by manual review of hundreds of prescriptions, plus disposition and robustness evaluations.

While these would require major changes, there are also some short term simpler improvements,

1. Adding a a brief expanded discussion on how an economic steward sub-agent could be integrated into the AIDOC framework would strengthen the future work section. This can take inspiration from MAI-DxO. While the diagnostic accuracy is high, the costs and efficiency are a concern.
Author Response: Thank you very much. We fully agree that explicit consideration of costs and efficiency is important. In response, we have expanded the Discussion to outline how an economic steward sub-agent, inspired by the cost-aware orchestration in MAI-DxO, could be integrated into our framework. In brief, we describe a dedicated sub-agent within a multi-agent system that monitors cumulative resource use (laboratory tests, imaging, procedures) in real time, cross-references guidelines, institutional SOPs and, where relevant, reimbursement catalogues, and flags or constraints low-value diagnostic choices while preserving guideline-concordant care. We also note that similar supervisory sub-agents (e.g., for ethical or regulatory considerations) could be layered on top of AIDOC's core, and have added this briefly given the overall word-count constraints in our article.

2. Better positioning the work in the spectrum between MAI-DXO, AMIE and OpenAI Penda Health in the intro.
Author Response: Thank you for this suggestion. In the Introduction, we have now explicitly positioned our work in relation to AMIE, MAI-DxO, and OpenAI's clinical copilot with Penda Health. Specifically, we describe AMIE as a conversational diagnostic system, MAI-DxO as a multi-agent diagnostic orchestrator, and the Penda Health copilot as a non-autonomous safety-net assistant in primary-care workflows, and we clarify that our contribution is a largely autonomous agent integrated into a EHR sandbox that manages full emergency-department care episodes. These changes appear in the second paragraph of the Introduction (starting "Several recent studies have explored AI agents in healthcare…"). We hope this fully addresses your concerns.

3. More expanded safety testing
Author Response: Thank you. We have addressed your concern with additional evaluations and explanations of our safety pipeline, please see above.

I wish the authors the best of luck with the work and applaud them on a fanstastic paper
Author Response: Thank you very much for your positive feedback and the time and efforts you have taken to review our manuscript. We believe that your comments have helped us to strengthen our results substantially and hope that our latest version addresses all your concerns.

Referee #2 (Remarks on code availability):

I briefly skimmed the code and it seems to be in order but I have not reviewed in detail.
Author Response: Thank you very much. We will make an updated version of the code available to the research community under MIT license.

Referee #4 (Remarks to the Author):

I co-reviewed this manuscript with one of the reviewers who provided the listed reports.
Author Response: Thank you very much for your time and effort and all your concerns and comments, that we believe have helped us improve our manuscript.

Referee #5 (Remarks to the Author):

I co-reviewed this manuscript with one of the reviewers who provided the listed reports.
Author Response: Thank you very much for your thoughtful feedback on our manuscript. We are confident that we have addressed all your remaining concerns in our latest version.

References for this “Response” Letter only:

1. Tu, T. *et al.* Towards conversational diagnostic artificial intelligence. *Nature* **642**, 442–450 (2025).
2. Hager, P. *et al.* Evaluation and mitigation of the limitations of large language models in clinical decision-making. *Nature Medicine* **30**, 2613–2622 (2024).
3. Linsky, A. & Simon, S. R. Medication discrepancies in integrated electronic health records. *BMJ quality & safety* **22**, (2013).
4. Andersen, T. S. *et al.* Medicines Reconciliation in the Emergency Department: Important Prescribing Discrepancies between the Shared Medication Record and Patients’ Actual Use of Medication. *Pharmaceuticals (Basel, Switzerland)* **15**, (2022).
5. https://www.hospitalmedicine.org/globalassets/clinical-topics/clinical-pdf/shm_medication_reconciliation_guide.pdf.
6. https://digitalassets.jointcommission.org/api/public/content/9be383450fc941df806b76c5fbdd9ae6?v=3c600c3a.
7. Cornish, P. L. *et al.* Unintended Medication Discrepancies at the Time of Hospital Admission. *Arch Intern Med* **165**, 424–429 (2005).
8. Johnsgård, T. *et al.* How much time do emergency department physicians spend on medication-related tasks? A time- and-motion study. *BMC emergency medicine* **24**, (2024).
9. https://www.hospitalmedicine.org/globalassets/clinical-topics/clinical-pdf/shm_medication_reconciliation_guide.pdf.
10. Rhodes, K. V. *et al.* Resuscitating the physician-patient relationship: emergency department communication in an academic medical center. *Annals of emergency medicine* **44**, (2004).
11. McCarthy, D. M. *et al.* Understanding patient-provider conversations: what are we talking about? *Academic emergency medicine : official journal of the Society for Academic Emergency Medicine* **20**, (2013).

12. https://arxiv.org/pdf/2405.07960.
13. https://arxiv.org/pdf/2505.04653v1.
14. Bokken, L. *et al.* Instructiveness of real patients and simulated patients in undergraduate medical education: a randomized experiment. *Academic medicine : journal of the Association of American Medical Colleges* **85**, (2010).
15. https://arxiv.org/pdf/2503.06074.

Rebuttal Letter for “*Towards Autonomous Medical Artificial Intelligence Agents*”

Author Responses to the Reviewers:

Referee #1 (Remarks to the Author):

Overall Assessment:

The authors have undertaken substantial additional work that significantly strengthens the manuscript. The patient agent validation experiments are thorough and well-designed, the expanded medication reconciliation analysis appropriately addresses precision concerns, and the thoughtful discussion of ground truth ambiguity is well-supported by literature. The system renaming resolves the naming conflict. I commend the authors for their responsiveness and the rigor of these additions.

Training Data Transparency:

In my previous review, I suggested scaling evaluation to several thousand cases to better assess generalization. The authors have declined this expansion, noting that their current sample size already exceeds related work. This is a reasonable position.

However, this decision makes transparent disclosure of a potential limitation more important: MIMIC-IV is a publicly available dataset (2008-2019) that could plausibly have been included in GPT-4o's training data. While OpenAI does not disclose training sources, readers should be made aware of this possibility when interpreting the performance results.

Suggested addition:

The authors should add a brief acknowledgment in the limitations section that MIMIC-IV is a publicly available dataset that may have been included in GPT-4o's training data, and that training data contamination cannot be ruled out. While this limitation affects benchmark validity broadly (not just this study), readers should be aware that the reported performance metrics may represent an upper bound rather than true generalization to unseen cases.

Author Response: Thank you very much for raising this important point. We agree that transparency regarding potential training data overlap is essential for proper interpretation of benchmark results. Although MIMIC-IV is publicly available, access requires credentialing, mandatory training, and a formal data use agreement, which substantially restrict dissemination and make inadvertent inclusion in foundation model pretraining corpora unlikely, even though complete exclusion cannot be guaranteed. To clarify this for readers, we have added the following note to the limitations section:

… Second, because MIMIC-IV is a widely used dataset that is publicly available to credentialed researchers, it could have been included in the training corpus of GPT-4o or other LLMs, although this seems unlikely because access requires prior registration. However, as training sources are not publicly disclosed, overlap cannot be fully ruled out; therefore, the reported performance on MIMIC-IV-derived cases could be cautiously interpreted as a possible upper bound and may overestimate generalization to truly unseen clinical cases (a limitation shared by many public-benchmark evaluations).

Thank you for the time and efforts you have done reviewing our manuscript over multiple rounds and the constructive feedback from you that has helped us strengthen our research. We hope we have addressed all of your points.

Referee #2 (Remarks to the Author):

The authors have provided a comprehensive and scientifically sound rebuttal. Thanks again for the dilligent work.

1. They addressed the primary concern (the perfect historian bias) with extensive new validation experiments (n=933 audits, adversarial testing) that suggest the agent is robust, even if the simulacrum limitation cannot be fully removed without a prospective trial.

2. The argument on the efficiency critique is addressed by showing the over-ordering is restricted to low-harm blood tests, not radiology.

3. They significantly expanded the safety evaluation to the full cohort, moving beyond the initial small sample.

4. The statistical treatment is appropriate. The use of paired comparisons (McNemar's test) for diagnostic accuracy and Wilcoxon signed-rank tests for resource utilization is correct, with proper adjustments for multiple comparisons (Benjamini-Hochberg).

Author Response: Thank you very much for appreciating our work and changes. Below we have addressed all of your remaining concerns.

The manuscript is now strong. I have only minor suggestions for the final version:

1. The discussion regarding the higher lab order rate (51% vs ~30%) is well-handled, but could be strengthened by explicitly linking the proposed economic steward sub-agent to specific high-cost examples from the data (e.g., explicitly contrasting the low marginal cost of extra blood labs vs. the high cost/harm of unnecessary imaging).

Author Response: Thank you for pointing this out. As suggested, we have added the following sentences to the discussion that include the suggested examples of low marginal cost blood tests versus high cost / potentially harmful imaging and directly link this to the suggested economic stewardship sub-agent:

> *… A related implementation consideration is resource stewardship. In these head-to-head evaluations, any incremental testing by MIRA was concentrated in low-marginal-cost blood analytes (for example, electrolytes and inflammatory markers), with no systematic increase in higher-cost cross-sectional imaging and radiation over-exposure relative to physicians. … This sub-agent would sit on top of the current tool-calling policy, double check test requests and suggest lower-cost but guideline-concordant alternatives when available with thresholds and guidance that place stricter scrutiny on additional high-cost imaging than on incremental low-marginal-cost blood tests.*

Due to limits in the main manuscript's word count, we have also extended this discussion in broader details in the Supplementary Information. Please refer to *Technical Extensions and Future Directions*.

2. Ensure the renaming from AIDOC to MIRA is thoroughly applied across all supplementary figures and tables to avoid reader confusion.

Author Response: Thank you for this important point. We have revised all submission materials to make sure that there is no naming conflict, and MIRA is used thoroughly across the manuscript.

While the limitation of using retrospective data remains, the authors have done everything possible in silico to validate their approach. The paper represents a substantial benchmark for the field.

Further demands for real patient validation would likely constitute a new study entirely.

Author Response: Thank you so much for the time and efforts you have taken and the substantial feedback throughout the review process that have helped us strengthen our manuscript. We fully agree that the next step in the evaluations of clinical AI agents need to be carefully designed (sandboxed) prospective evaluations in hospitals, which we emphasize in the discussion.

Referee #2 (Remarks on code availability):

I have skimmed over the code but not in detail.

Author Response: Thank you very much for your helpful feedback on our manuscript.

Referee #4 (Remarks to the Author):

I co-reviewed this manuscript with one of the reviewers who provided the listed reports.

Author Response: Thank you very much for your time and efforts and thoughtful feedback on our work.

Referee #4 (Remarks on code availability):

I have reviewed the code and there is no obvious mistake.

Author Response: Thank you very much. We will make the code available to researchers with publication of this manuscript.

Response Letter for "*Towards Autonomous Medical Artificial Intelligence Agents*"

Author Responses to the Editors:

Response to 514841_3_attach_24_9688.pdf

„Please see the extended comments document for a list of figures and figure legends that require additional information."

Author Response: *Thank you. We have addressed all your remaining concerns from 514841_3_attach_25_19903.docx and answered all your points in there.*

„Please ensure the following software/packages are mentioned in the manuscript as well, since they have been listed in the reporting summary. For example: about-time.

Please provide the GitHub web-link for the custom codes developed in the study, here in the reporting summary as provided in the manuscript.

Please ensure that all information on availability of codes should be provided in the code availability section of the manuscript."

Author Response: *Thank you. We have revised the manuscript so that all information on code access is provided in the Code availability section, including the GitHub link to the custom code used in this study (https://github.com/Dyke-F/MIRA). We help the user by distinguishing between core data-analysis software used for statistical analysis and visualization, and auxiliary runtime or installation dependencies that support execution of the codebase which are not themselves data-analysis tools. Packages such as about-time fall into this latter category, so instead of listing all such utilities individually in the manuscript or in the "Data analysis" field of the reporting summary, we now point to the repository's dependency files, `src/pyproject.toml` and `src/uv.lock`, which together provide the complete reproducible software environment, including the exact package versions used.*

"Please provide a complete data availability statement here in the reporting summary, as provided in the manuscript.

Please ensure that only the information related to databases/datasets generated and/or used in the study are mentioned in the data availability section of the manuscript. Information regarding code availability should be provided directly in the code availability section of the manuscript."

Author Response: *Thank you. We have adapted the „Data and Code Availability Statements“ accordingly.*

“Please provide a rationale for why these sample sizes are sufficient.”

“Please describe how covariates were controlled or if this was not applicable to the study, elaborate as to why.”

Author Response: *Thank you. We have revised the Reporting Summary to clarify both points. Specifically, we now state that the final sample size (n = 574) was not determined by a formal a priori calculation because this was a retrospective benchmark based on a fixed cohort, and that all eligible cases meeting prespecified selection and data-completeness criteria were included. We also clarify that formal covariate adjustment was not applicable for the full-cohort analyses and that, in the head-to-head evaluations, covariates were controlled by design because MIRA and physicians assessed the same patient cases under identical information conditions, with analyses restricted to paired cases assessed by both groups.*

Author Responses to the Reviewers:

Referee #1 (Remarks to the Author):

No further comments. I thank the authors for their responses over multiple rounds, which has led to a much strengthened manuscript.

Author Response: *We sincerely would like to thank you for your thoughtful and constructive feedback throughout the review process. All the points you have raised have been very helpful to us in strengthening our work.*

Referee #2 (Remarks to the Author):

The authors have been very responsive throughout the peer review process and have addressed my remaining minor comments in this final revision.

Specifically, the addition of the explicit contrasting examples in the Discussion highlighting the low marginal cost of incremental blood labs versus the high cost and potential harm of unnecessary cross-sectional imaging helps contextualizes the proposed economic steward sub-agent. This provides a much clearer framework for how resource stewardship should be evaluated in future iterations of medical AI agents.

Furthermore, I appreciate the transparent acknowledgment regarding the potential for GPT-4o training data overlap with the MIMIC-IV dataset (as suggested by Reviewer 1).

While the reliance on retrospective data from a single academic center remains an inherent limitation of this work, the authors have done everything possible in silico to validate their approach, bound their claims and transparently discuss these caveats.

The resulting study establishes a much-needed, reproducible benchmark for evaluating autonomous EHR-integrated clinical AI.

I commend the authors on an excellent and significant contribution to the field. I have no further requests.

Referee #2 (Remarks on code availability):

I briefly skimmed and dont see any major issues

Author Response: *We are very grateful for your thorough and constructive feedback with our work across all rounds of revision. Your suggestions have meaningfully improved the clarity and rigor of our research.*

Referee #4 (Remarks to the Author):

I co-reviewed this manuscript with one of the reviewers who provided the listed reports.

Author Response: *We thank you for your time and valuable contributions throughout the review process.*
